# Natural formulas and the nature of formulas: Exploring potential therapeutic targets based on traditional Chinese herbal formulas

**DOI:** 10.1371/journal.pone.0171628

**Published:** 2017-02-09

**Authors:** Qianru Zhang, Hua Yu, Jin Qi, Daisheng Tang, Xiaojia Chen, Jian-bo Wan, Peng Li, Hao Hu, Yi-tao Wang, Yuanjia Hu

**Affiliations:** 1 State Key Laboratory of Quality Research in Chinese Medicine, Institute of Chinese Medical Sciences, University of Macau, Macau, the People’s Republic of China; 2 Pharmacy School, Zunyi Medical College, Zunyi, Guizhou, the People’s Republic of China; 3 Department of Complex Prescription of Traditional Chinese Medicine, China Pharmaceutical University, Nanjing, the People’s Republic of China; 4 Beijing Jiaotong University, Beijing, the People’s Republic of China; Semmelweis University, HUNGARY

## Abstract

By comparing the target proteins (TPs) of classic traditional Chinese medicine (TCM) herbal formulas and modern drugs used for treating coronary artery disease (CAD), this study aimed to identify potential therapeutic TPs for treating CAD. Based on the theory of TCM, the Xuefu-Zhuyu decoction (XZD) and Gualou-Xiebai-Banxia decoction (GXBD), both of which are classic herbal formulas, were selected for treating CAD. Data on the chemical ingredients and corresponding TPs of the herbs in these two formulas and data on modern drugs approved for treating CAD and related TPs were retrieved from professional TCM and bioinformatics databases. Based on the associations between the drugs or ingredients and their TPs, the TP networks of XZD, GXBD, and modern drugs approved for treating CAD were constructed separately and then integrated to create a complex master network in which the vertices represent the TPs and the edges, the ingredients or drugs that are linked to the TPs. The reliability of this master network was validated through statistical tests. The common TPs of the two herbal formulas have a higher possibility of being targeted by modern drugs in comparison with the formula-specific TPs. A total of 114 common XZD and GXBD TPs that are not yet the target of modern drugs used for treating CAD should be experimentally investigated as potential therapeutic targets for treating CAD. Among these TPs, the top 10 are NOS3, PTPN1, GABRA1, PRKACA, CDK2, MAOB, ESR1, ADH1C, ADH1B, and AKR1B1. The results of this study provide a valuable reference for further experimental investigations of therapeutic targets for CAD. The established method shows promise for searching for potential therapeutic TPs based on herbal formulas. It is crucial for this work to select beneficial therapeutic targets of TCM, typical TCM syndromes, and corresponding classic formulas.

## Introduction

Traditional Chinese medicine (TCM) is rooted in thousands of years of history and is one of the forms of alternative medicine endorsed by the World Health Organization [1]. In the last two decades, an increasing number of people worldwide have used TCM, especially for managing chronic diseases [2–4]. Unlike Western medicine, which tends to see disease in terms of the body part that presents symptoms [5–7], TCM emphasizes a holistic and systemic approach that treats the organism and views disease not as an independently pathological progress but as an imbalance of the body induced by multiple external or internal factors between opposite physiological functions symbolically described as ying/yang, deficiency/excess, cold/heat, *etc*. [8, 9].

Chinese herbal medicine (CM) is another important modality of TCM for restoring the body’s balance and preventing or treating illness. In practice, multiple-herb formulas (TCM formulas), instead of a single herb, are more commonly used to achieve optimal therapeutic efficacy [10]. The interactions between the components of different CMs are thought to produce certain competitive and/or synergistic effects on multiple target proteins (TPs), thus improving the pharmacological activities and/or reducing the adverse clinical reactions caused by some individual herbs [11, 12]. As the therapeutic TPs of TCM formulas are abundant but have been investigated on a limited basis, identifying potential therapeutic TPs based on CM formulas and further elucidating their beneficial effect on the treatment of diseases is important [13].

However, studies on TCM are always controversial in terms of their abstract theory, unclear basis, complex interactions between various ingredients and complex interactive biological systems, and inadequate quality control. With the limited rigorous scientific evidence of its effectiveness, TCM can be difficult for researchers to study because its treatments are often complex and are based on ideas very different from those of modern Western medicine.

Network pharmacology integrates information from bioinformatics, systems biology, and polypharmacology and provides a platform for integrating multiple components and interactions underlying cell, organ, and organism processes in health and disease [14]. This novel method challenges the traditional paradigm of single target drug discovery and explores multiple interactions among “genes, drugs, [and] diseases” from a global perspective [6]. A holistic system mapped by network pharmacology highlights the synthetic effects of multiple drugs on biological networks and relevant diseases [15, 16]. Moreover, network pharmacology offers a new strategy for promoting a significant transformation in strategies for therapies and novel drug discovery [13, 17].

Coinciding with the holistic and systemic characteristics of TCM, network pharmacology is expected to bridge the gap between TCM and modern medicine [18, 19]. The established network will not only help improve our understanding of the chemical-activity relationship of CMs but also facilitate the identification of their main active ingredients and therapeutic TPs [20]. A network-based approach has been widely used to discover drug candidates from herbal products [21], predict potential therapeutic TPs of CMs or herbal prescriptions [22, 23], understand the biological significance of TCM syndromes in the differentiation of disease [24], and illuminate the mechanisms of the global functional regulation of CMs [25, 26].

To explore the use of the network-based approach to identify potential therapeutic TPs from TCM formulas, this study focuses on coronary artery disease (CAD), as CAD is now the leading cause of mortality worldwide [27], and the biological pathways and therapeutic TPs for treating CAD have been widely investigated and partially identified [28]. Based on TCM diagnostic methods, CAD can be clinically differentiated as various syndromes, such as blood stasis, phlegm turbidity, qi stagnation, cold coagulation, yin deficiency, yang deficiency, and qi deficiency [29]. Among these syndromes, blood stasis and phlegm turbidity are the two major CAD syndromes [30, 31]. From a practical standpoint, numerous TCM formulas with different compositions have been demonstrated as being safe and effective for treating CAD [24]. Therefore, we hypothesized that the chemicals in these formulas might focus on similar and crucial TPs that play important roles at the molecular level.

In TCM clinical practice, physicians usually follow the “one classic formula for one typical syndrome” principle. In this case, the Xuefu-Zhuyu decoction (XZD) and Gualou-Xiebai-Banxia decoction (GXBD) are two classic formulas used for treating blood stasis and phlegm turbidity, respectively [21, 32]. Based on a nationwide expert survey on the application of TCM in different clinical classifications of CAD, XZD and GXBD were listed as the two most widely used formulas in TCM clinical therapy for treating angina pectoris and acute myocardial infarction [33]. Meanwhile, experimental evidence of their safety and efficacy has been widely reported in existing literature [34–41]. For instance, a nuclear magnetic resonance (NMR)-based metabolomics study demonstrated that XZD could effectively ameliorate the symptoms of hyperlipidemia on a global scale and regulate the metabolic state to a near-normal level in a time-dependent pattern [36]. A pharmacological research showed that GXBD could effectively prevent the elevation of segment ST and myocardial damage in rats with acute myocardial ischemia with confidence level of 95% [40]. A meta-analysis further validated the clinical efficacy of GXBD for treating unstable angina [41]. Thus, by using the two herbal formulas as an example, the study constructs biologically meaningful networks to elucidate the target associations based on the components and further identifies potential TPs for treating CAD.

## Material and methods

### Data

XZD and GXBD are two typical herbal formulas used in the TCM treatment of CAD. XZD comprises 11 CMs: Radix Bupleuri, Radix Angelicae Sinensis, Radix Rehmanniae, Radix Paeoniae Rubra, Flos Carthami, Semen Persicae, Fructus Aurantii, Radix et Rhizoma Glycyrrhizae, Rhizoma Chuanxiong, Radix Achyranthis Bidentatae, and Radix Platycodonis [32, 35]. GXBD comprises three CMs: Fructus Trichosanthis, Bulbus Allii Macrostemonis, and Rhizome Pinelliae [32]. The chemical constituents of these 14 CMs were retrieved from a traditional Chinese medicine system pharmacology database (TCMSP) and a traditional Chinese medicine integrated database (TCMID) [42, 43]; the CMs’ corresponding TPs were retrieved from TCMSP. In addition, 240 TPs of drugs approved for use for treating CAD by the US Food and Drug Administration (FDA) [44, 45] were collected from Drugbank and the Kyoto Encyclopedia of Genes and Genomes (KEGG), two professional bioinformatics databases [46, 47]. The similarity of XZD and GXBD was assessed at the molecular level using the Jaccard index (JI), which measures the similarity of finite sample sets and is calculated as the size of the intersection divided by the size of the union of the sample sets. The value of JI is between 0 and 1, and a higher value of JI implies greater similarity [48].

### Target network construction

A TCM formula comprises different CMs and contains various chemical ingredients. Some of these ingredients can act with different functional proteins *in vivo*, thus triggering a synergistic response [49, 50]. With the information obtained from the databases, a bipartite network (drug-target [DT] network) can be constructed based on the interactions between the ingredients and the relevant protein targets. Subsequently, the DT network can be transformed into two biologically relevant network projections (drug network and TP network) through matrix algebra [51]. The nodes in the drug network represent drugs or ingredients, and two nodes are connected to each other if they share at least one TP; the nodes in the TP network are proteins, and two proteins are connected if they are both targeted by at least one common drug or ingredient [15].

The TP network graph *G* = (*V*, *E*) is a combination of *V* and *E*, where *V* is a set of vertices that represent the TPs and *E* is a set of edges, which means at least one chemical ingredient links *v*_*i*_ to *v*_*j*_ [15]. The TP networks of XZD and GXBD were visualized and analyzed using the software package Gephi [52].

### Centrality analysis

According to the principle of graph theory, the significance of vertices can be measured and expressed using centrality. Centrality indicators identify the most important vertices within the graph [53–55]. In this study, three centrality measurements (degree, betweenness, and closeness) were adopted to assess different aspects of the positions of the TPs in the TP network, where vertices represent proteins and edges, drugs/ingredients. Degree centrality shows the number of drugs/ingredients associated with a TP. Betweenness centrality measures how often a protein as an intermediary appears on the shortest path between two proteins. An intermediary with high betweenness functions as a “gatekeeper” to control the flow of interactions in the network [56]; in other words, this protein plays a critical role in intermediating other proteins in terms of the investigated drugs/ingredients in this study. However, a high-betweenness protein need not necessarily be one with a high degree centrality. The closeness centrality of a protein is the total geodesic distance between a protein and all other proteins; it can be defined as how close a protein is to all others. A lower closeness value indicates that it is a more central protein [55, 57]. Three centrality indicators were calculated using the software package Gephi [52, 58].

### Statistical analysis

On the basis of the above data, target network, and centrality analysis, a network-based approach can be employed to elucidate complex associations between targets and to estimate potential targets after passing statistical validation. For quality control, a random simulation should be performed to see whether the results are significant. In the constructed network of targets, various statistical tests will be conducted to examine the associations between variables, especially variables generated from independent data sources, for instance, target modules from different formulae, centrality indicators, and the novelty relative to existing drug targets. According to the types of variables, this study will adopt various appropriate statistical testing approaches, for example, chi-squared test, t-test, one-way analysis of variance (ANOVA), and Pearson correlation test [59, 60].

## Results

### Comparison of two TCM formulas

In this study, 787 components of XZD and 179 components of GXBD were generated from the TCMSP and TCMID databases (S1 and S2 Tables). The types of chemicals in XZD include terpenoids (26.9%), flavonoids (15.0%), phenylpropanoids (9.3%), alkaloids (4.8%), sterides (4.3%), phenolic acids (2.7%), saccharides (2.0%), and quinones (0.9%). The types of chemicals in XZD include volatile oil (38.0%), alkaloids (16.2%), sterides (11.7%), phenylpropanoids (11.2%), and flavonoids (6.1%). Based on the comparison of the similarity of the chemicals between the two formulas, the low JI value of 6.62% implies that the chemical compositions of the formulas are significantly different (S3 Table). Some of the components of XZD and GXBD have been pharmacologically validated as being effective for treating cerebrovascular and cardiovascular diseases through various therapeutic targets [61–68].

As summarized in Table 1, 787 ingredients in XZD and 179 ingredients in GXBD were found to be associated with 214 and 178 therapeutic TPs, respectively (S4 and S5 Tables). About the similarity reflected by the JI, only a few ingredients between XZD and GXBD were mutual with a low value of 6.62%, whereas the therapeutic TPs for the two formulas highly overlapped with a much higher value of 71.93% in total and 80.65% in the drugs’ TPs for treating CAD. The results suggested that XZD and GXBD may act on similar protein targets at the biomolecular level to generate common therapeutic effects, including treating CAD, whereas the formulas’ chemical compositions were substantially different.

**Table 1:** Similarity analysis of XZD and GXBD.

	No. of CMs	No. of retrieved ingredients	No. of therapeutic target proteins		
			Targets of FDA-approved drugs for CAD	Others	Total
XZD	11	787	62	152	214
GXBD	3	179	50	128	178
JI	0	0.0662	0.8065	0.6867	0.7193

### Target protein network

**Fig 1:**
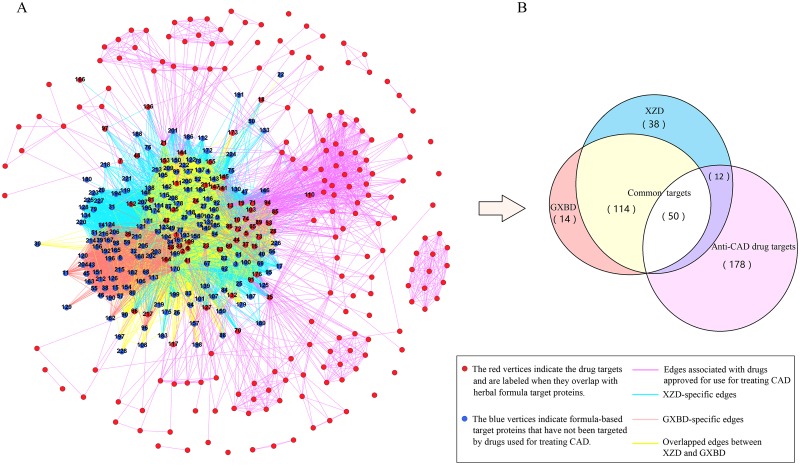
Master network of modern drugs used for treating CAD. (A) Formula-based target network in the context of drug targets. The vertex represents the target protein. The blue vertices indicate formula-based target proteins that have not been targeted by drugs used for treating CAD, and the red vertices indicate the drug targets and are labeled when they overlap with herbal formula target proteins. A colored edge indicates a drug or compound linked to two target proteins (blue: XZD-specific edges; orange: GXBD-specific edges; yellow: overlapped edges between XZD and GXBD; and purple: edges associated with drugs approved for use for treating CAD). (B) The distribution of different target proteins in the network (yellow: common XZD and GXBD target proteins that have not been targeted by modern drugs used for treating CAD; white: common XZD and GXBD target proteins targeted by modern drugs used for treating CAD; orange: GXBD-specific target proteins; blue: XZD-specific target proteins that have not been targeted by drugs used for treating CAD; dark purple: XZD-specific proteins targeted by drugs used for treating CAD; and light purple: target proteins specific to drugs used for treating CAD). The numbers in parentheses represent the number of target proteins in each specific set.

The XZD and GXBD TP networks were constructed separately and then integrated. As illustrated in Fig 1A, the TP networks of XZD, GXBD, and drugs used for treating CAD (S6 Table) were constructed separately with 7664 edges with 214 vertices, 6374 edges with 178 vertices, and 1418 edges with 240 vertices, respectively, and then integrated to create a complex master network. The blue vertices indicate formula-based TPs that have not been targeted by drugs used for treating CAD, whereas the red vertices are the drug targets and are labeled when they overlap with herbal formula TPs. The blue, orange, yellow, and purple edges denote XZD-specific ingredients, GXBD-specific ingredients, ingredients with common XZD and GXBD TPs, and drugs used for treating CAD, respectively. The distribution of the TPs in the network is summarized and illustrated in Fig 1B. The colored areas in Fig 1B represent the 114 common XZD and GXBD TPs that have not been targeted by modern drugs used for treating CAD, 50 common XZD and GXBD TPs targeted by modern drugs used for treating CAD, 14 GXBD-specific TPs, 38 XZD-specific TPs that have not been targeted by drugs used for treating CAD, 12 XZD-specific TPs targeted by drugs used for treating CAD, and 178 TPs specific to drugs used for treating CAD. To uncover the potential CAD-related therapeutic TPs of XZD and GXBD, 228 TPs of the herbal formulas were analyzed.

### Network analysis

Fig 1 shows the ingredients (edges) and corresponding TPs (vertices) of the two herbal formulas and the modern drugs approved for treating CAD. By comparing the similarities and differences of the TPs in this master network between the two representative herbal formulas and modern drugs used for treating CAD, this study expects to find potential TPs for treating CAD. To ensure the reliability of the screening *in silico* of the therapeutic TPs, the master network must first be validated statistically.

As shown in Fig 1B, a total of 228 therapeutic TPs of XZD and GXBD are divided into five modules (yellow, white, orange, blue, and dark purple parts) according to their interactions with the TCM formulas and drugs. The centrality indicators identify the important vertices within the network. A dummy variable, the TP of a drug used for treating CAD (0: no; 1: yes), is used to reflect whether a protein is targeted by modern drugs approved for treating CAD.

According to the types of variables, various statistical tests were conducted to examine the associations between the variables (i.e., network modules, three centralities, and drug target for treating CAD). The chi-square test analyzed the association between the dummy variable “drug target proteins” and the categorical variable “modules”; the independent samples *t*-test, that between “drug target proteins” and “centralities”; one-way ANOVA, that between “modules” and “centralities”; and Pearson correlation tests, those between the three “centralities” indicators. As a result, all of the above variables show significant associations at 95% confidence level. These statistical results further imply that the master network is not random but reflects, to some extent, chemical or biological significance. The details are discussed below.

First, in terms of the aim of this study, whether the GXBD-specific target proteins, XZD-specific target proteins, and common XZD and GXBD target proteins are significantly different from the TPs of modern drugs used for treating CAD should be validated. In other words, the percentages of the TPs of modern drugs used for treating CAD should be tested among the three samples generated from the combinations of the five modules. The differences in the percentages of the TPs of modern drugs used for treating CAD among specific and common parts of two formulas were statistically significant at the 0.05 level with a chi-square value of 6.386 and *p* value of 0.041. A contingency table for the chi-squared test is shown in Table 2.

**Table 2:** Contingency table for chi-squared test.

		Target proteins of modern drugs		Total
		No	Yes	
Target proteins in different modules	GXBD-specific	14 (10.2)	0 (3.8)	14 (14.0)
	XZD-specific	38 (36.4)	12 (13.6)	50 (50.0)
	Overlapping	114 (119.4)	50 (44.6)	164 (164.0)
Total		62 (62.0)	166 (166.0)	228 (228.0)

Note: (1) Cell values denote observed counts, and numbers in parentheses indicate expected counts; (2) 1 cell (16.7%) has expected count less than 5 and the minimum expected count is 3.8.

Furthermore, the TPs of the FDA-approved drugs for treating CAD in the master network played more crucial roles than the others with *t* values (*p* values) of independent samples tests of degree, closeness, and betweeness between drug targets and non-drug targets being -2.783 (0.007), 3.501 (0.001), and -5.514 (<0.001), respectively. A one-way ANOVA also indicated that the degree was significantly different among the five target modules with *F* value (*p* value) of 24.889 (<0.001), and another two one-way ANOVA tests for the closeness and betweeness among modules had values of 29.679 (<0.001) and 23.742 (<0.001), respectively. Finally, the associations between the three “centralities,” that is, degree-closeness, degree-betweenness, and closeness-betweenness, are statistically significant with Pearson correlation coefficients (*p* value) of -0.937 (<0.001), 0.533 (<0.001), and -0.486 (<0.001), respectively.

After the above quality control of the master network, the network analysis results are discussed as below. In the complex master network shown in Fig 1B, the common XZD and GXBD TPs (S7 Table) have a higher possibility (43.8%) of being targeted by modern drugs in comparison with XZD-specific TPs (24%) and GXBD-specific TPs (0%). Details of the observed and expected counts are shown in Table 2. The 114 common XZD and GXBD TPs that have not been targeted by modern drugs used for treating CAD should be further investigated as potential therapeutic targets for CAD. Furthermore, centrality measures can identify the importance of specific nodes in the whole network. Based on the definitions of three centrality indicators (degree, betweenness, and closeness) on the part of material and methods, different centralities reflect different importance of nodes in a network from different angles. Especially, in terms of the correlation relationships with different strengths among the three centralities in this study, it is necessary to combine multiple centrality indicators to identify important target proteins.

With this approach, the top 10 mutual targets worthy of further investigation in the context of new drug discovery are generated and summarized in Table 3. Although different types of centrality indicators are adopted, interestingly, the top 10 targets are basically consistent (i.e., NOS3, PTPN1, GABRA1, PRKACA, CDK2, MAOB, ESR1, ADH1C, ADH1B and AKR1B1). They are discussed below.

**Table 3:** Top 10 target proteins according to three centrality indicators.

Proteins	Degree	Proteins	Betweenness	Proteins	Closeness
NOS3	192	NOS3	1585.3080	NOS3	1.5324
PTPN1	181	CDK2	983.2459	PTPN1	1.5892
GABRA1	176	PTPN1	836.1325	CDK2	1.5919
PRKACA	173	ESR1	810.6359	GABRA1	1.6189
CDK2	168	ADH1C	709.8504	MAOB	1.6216
MAOB	162	MAOB	636.5054	PRKACA	1.6324
ESR1	159	ADH1B	614.3183	ESR1	1.6486
ADH1C	151	GABRA1	607.0142	ADH1C	1.6622
AKR1B1	148	PRKACA	590.1397	ADH1B	1.6865
TNF	145	ALOX5	413.5665	AKR1B1	1.6946

Note: The centrality indicators identify the important vertices within the network. Higher degree centrality and betweenness centrality indicate greater importance, whereas lower closeness centrality indicates greater importance.

Meanwhile, the common proteins which have been validated as targets of approved anti-CAD drugs, i.e., the 50 mutual targets covered by the two formulas and anti-CAD drugs in Fig 1B, are also useful to add to the current understanding of the disease mechanism and to develop new therapeutic agents based on existing targets. They include many well-known targets of CAD, for example, PTGS2, NOS2, and F2. All common targets in Fig 1B are listed in S7 Table.

## Discussion

The drug network can be seen as a measure to explore the synergy between drugs because drugs targeting the same target are connected in the network. However, the interpretation of the TP network seems difficult. Targets intervened by the same compound (not targets of intrinsic connections in biological functions) are linked together. On the one hand, it may be implied that herbal formulas generate a multicomponent and multitarget therapeutic mechanism under the precondition of the safety and efficacy of formulas. Thus, the TP network can be applied to explore potential “new” therapeutic targets, as discussed in results. On the other hand, there exists another possibility if the precondition about the TCM formulas is insufficient, namely, the TP network may imply side effects in that targets functioning in different biological functions are always interfered together by the formulas. Thus, it is still necessary to examine the biological meaning of the targets found in the network analysis sufficiently, although we comprehensively reviewed XZD and GXBD in terms of clinical utilization in TCM therapy for treating CAD and established an experimental basis before conducting this study.

Generally, the association of top mutual TPs with CAD and relevant biological significance have been widely discussed in existing literature. For instance, NOS3 (endothelial nitric oxide synthase) is the nitric oxide synthase isoform responsible for maintaining systemic blood pressure, vascular remodeling and angiogenesis, and vascular smooth muscle relaxation through directly regulating NO production [69–71]. PTPN1 (tyrosine-protein phosphatase non-receptor type 1) is implicated as contributing to the negative regulation of insulin signaling and a key regulator of cardiovascular effects by reducing vascular adrenergic reactivity [72, 73]. PRKACA (cAMP dependent kinase) can maintain circulating platelets in a resting state by phosphorylating proteins in numerous platelet-inhibitory pathways [74]. CDK2 (Cyclin-dependent kinase 2) plays an important role in altering the phosphorylation profile of retinoblastoma tumor suppressor protein (Rb) in coronary artery smooth muscle cells (SMCs) as well as the proliferative response of these cells to mitogenic stimulation [75]. Moreover, MAO-B (amine oxidase B) inhibitors have been shown to be potentially beneficial for treating cardiovascular pathologies [76]. ESR1 (estrogen receptor) could directly affect cardiovascular tissues via regulating the expression of inducible nitric oxide synthase (NOS2A) in vascular smooth muscle cells (VSMC) [77]. ADH1C and ADH1B (alcohol dehydrogenase 1C and 1B) play important roles in modulating fibrinogen and increasing insulin sensitivity to alter the risk of CAD in persons with a history of long-term alcohol consumption [78]. AKR1B1 (aldose reductase) protects against heart ischemic injury by preventing ER stress induced by excessive accumulation of aldehyde-modified proteins [79]. All of these indicate that the top mutual targets perform various beneficial functions for treating cardiovascular diseases at the molecular level. The associations and biological significance of these targets with CAD provide potential opportunities for the further discovery of new drugs for treating CAD.

As stated in the introduction, TCM follows the principle of “one classic formula for one typical syndrome,” and XZD and GXBD are intended for two different syndromes in the TCM domain. TCM is well known and considered attractive for its synergistic effects that are observable at the physiological level. On the other hand, modern drugs follow the reductionism approach, which identifies the single most potent compound for one objective. In TCM and modern medicine theory, the definition of syndromes is quite different. The modern CAD concept covers the TCM blood stasis and phlegm turbidity concepts. Such differences might explain the high frequency of modern CAD drugs targeting overlapping targets.

However, there is no reason to consider the formulae-specific targets unimportant. Actually, this is an area not yet well explored by the modern reductionism drug discovery research. Although XZD and GXBD are two classic formulas for treating CAD, they are used differently in TCM clinical practice for two different syndromes, i.e., blood stasis and phlegm turbidity, respectively. The scientific mechanism of TCM syndromes is not yet clear; however, blood stasis and phlegm turbidity provide a valuable basis for studying CAD subtypes, especially under the background of the emerging medical model of precision medicine for customizing healthcare. Thus, formula-specific targets are still worthy of further experimental investigation based on formula-specific clinical applications of TCM and precision medicine, with the aim of exploring therapeutic targets for CAD.

As illustrated in Fig 1B, 50 overlapping TPs between two formulas and 12 XZD-specific ones have been regarded as TPs of modern drugs used for treating CAD. The 38 XZD-specific TPs that are not targeted by drugs used for treating CAD might be used for identifying potential therapeutic targets for CAD in the context of the specific clinical applications of XZD. Meanwhile, 14 GXBD-specific TPs were identified as being irrelevant to the current TPs of drugs used for treating CAD and could potentially be used for treating promising therapeutic targets for CAD based on GXBD-specific clinical applications. With these studies in mind, the different bioactivities for each formula could be enumerated, and the unique therapeutic targets for CAD in each formula could be identified.

Several limitations of this study should be noted. First, as a *silico* study, this work is still weak owing to the lack of experimental validation. In particular, the top 10 mutual targets proteins are only “potential” therapeutic targets for CAD and need to be validated by specific pharmacological investigations. The results of this study only provide a reference for further experimental investigations of therapeutic targets for CAD. Second, more powerful herbal and bioinformatics databases, for example, the Herbal Ingredients Targets Database (HIT) and the Therapeutic Target Database (TTD), should be included in future studies for better-quality data. In addition, the TCM ingredients/targets/functions provided in currently accessible repositories may not be comprehensive. Potential bias may be caused by the type of limited source data. Sensitivity analysis and negative control should be performed in future studies to assess the robustness of research results and conclusions against source data quality.

## Conclusions

The therapeutic effects of herbal formulas in disease management have been demonstrated by clinical practice over thousands of years. Numerous TPs of chemical ingredients combined in herbal formulas have been identified by modern pharmacological studies on TCM, although the overall mechanism of TCM has not been elucidated. By comparing the similarities and differences in TPs between herbal formulas and modern pharmaceutical agents, potential TPs for further experimental investigation can be identified. This study examined two herbal formulas used for treating CAD as an example for exploring a new methodology based on finding therapeutic TPs.

The beneficial therapeutic areas of TCM should be clarified in the context of modern medicine. Classic formulas corresponding to typical syndromes in these therapeutic areas should be selected. Based on the approaches used in this study, a series of potential TPs of the herbal formulas are promising for future experimental study.

## Supporting information

S1 Table Chemical compounds in XZD herbal medicines. (DOCX)

S2 Table Chemical compounds in GXBD herbal medicines. (DOCX)

S3 Table Common XZD and GXBD chemical compounds. (DOCX)

S4 Table Target proteins of XZD herbal medicines. (DOCX)

S5 Table Target proteins of GXBD herbal medicines. (DOCX)

S6 Table Target proteins of anti-CAD drugs. (DOCX)

S7 Table Common proteins of XZD and GXBD. (DOCX)

## References

[1] World Health Organization. WHO Traditional Medicine Strategy 2002–2005. [Updated 18 Jun 2002; cited 10 Mar 2015]. http://whqlibdoc.who.int/hq/2002/WHO_EDM_TRM_2002.1.pdf?ua=1.

[2] Coyle M, Shergis JL, Liu S, Wu L, Zhang AL, Guo X, et al Safety of Chinese herbal medicine for chronic obstructive pulmonary disease. Evid Based Complement Alternat Med. 2015; 380678 10.1155/2015/380678 25883670 PMC4391162

[3] Wang YY, Li XX, Liu JP, Luo H, Ma LX, Alraek T. Traditional Chinese medicine for chronic fatigue syndrome: a systematic review of randomized clinical trials. Complement Ther Med. 2014; 22:826–833. 10.1016/j.ctim.2014.06.004 25146086

[4] Qi FH, Wang ZX, Cai PP, Zhao L, Gao JJ, Kokudo N, et al Traditional Chinese medicine and related active compounds: a review of their role on hepatitis B virus infection. Drug Discov Ther. 2013; 7(6):212–224. 24423652 10.5582/ddt.2013.v7.6.212

[5] Cheung F. TCM: made in China. Nature. 2011; 480(7378):S82–83. 10.1038/480S82a 22190085

[6] Hopkins AL. Network pharmacology. Nature biotechnology. 2007; 25:1110–1111. 10.1038/nbt1007-1110 17921993

[7] Chan K. Chinese Medicinal materials and their interface with western medical concepts. J Ethnopharmacol. 2005; 96(1):1–18. 15588645 10.1016/j.jep.2004.09.019

[8] Lozano F. Basic Theories of Traditional Chinese Medicine Acupuncture for Pain Management. Springer, 2014: 13–43.

[9] Jiang WY. Therapeutic wisdom in traditional Chinese medicine: A perspective from modern science. Trends Pharmacol Sci. 2005; 26(11):558–563. 10.1016/j.tips.2005.09.006 16185775

[10] Pan SY, Chen SB, Dong HG, Yu ZL, Dong JC, Long ZX, et al New perspectives on Chinese herbal medicine (zhong-yao) research and development. Evid Based Complement Alternat Med. 2011; 2011:403709 10.1093/ecam/neq056 21785622 PMC3135515

[11] Xu YT. Modern scientific connotation on formula compatibility in Chinese materia medica. Chin Tradit Herbal Drugs. 2015; 46:465–469.

[12] Guo H, Li W, Wang X, Fan G. Advance of research on toxic attenuation by compatibility of traditional Chinese medicine prescriptions. Zhongguo Zhong Yao Za Zhi. 2012; 37(1):120–123. 22741475

[13] Pei L, Bao Y, Liu S, Zheng J, Chen X. Material basis of Chinese herbal formulae explored by combining pharmacokinetics with network pharmacology. PLoS One. 2013; 8:e57414 10.1371/journal.pone.0057414 23468985 PMC3585395

[14] Hopkins AL. Network pharmacology: the next paradigm in drug discovery. Nat Chem Biol. 2008; 4(11):682–690. 10.1038/nchembio.118 18936753

[15] Yildirim MA, Goh KI, Cusick ME, Barabasi AL, Vidal Marc. Drug-target network. Nat Biotechnol. 2007; 25(10):1119–1126. 10.1038/nbt1338 17921997

[16] Levinson AD. Cancer therapy reform. Science. 2010; 328(5975):137 10.1126/science.1189749 20378778

[17] Barabási AL, Gulbahce N, Loscalzo J. Network medicine: a network-based approach to human disease. Nat Rev Genet. 2011; 12(1):56–68. 10.1038/nrg2918 21164525 PMC3140052

[18] Zhang GB, Li QY, Chen QL, Su SB. Network pharmacology: a new approach for Chinese herbal medicine research. Evid Based Complement Alternat Med. 2013; 2013:621423 10.1155/2013/621423 23762149 PMC3671675

[19] Li S, Zhang B. Traditional Chinese medicine network pharmacology: theory, methodology and application. Chin J Nat Med. 2013; 11(2):110–120. 10.1016/S1875-5364(13)60037-0 23787177

[20] Hao da C, Xiao PG. Network pharmacology: a Rosetta stone for traditional Chinese medicine. Drug Dev Res. 2014; 75:299–312. 10.1002/ddr.21214 25160070

[21] Ding F, Zhang Q, Ung CO, Wang Y, Han Y, Hu Y, et al An analysis of chemical ingredients network of Chinese herbal formulae for the treatment of coronary heart disease. PLoS One. 2015; 10:e0116441 10.1371/journal.pone.0116441 25658855 PMC4319923

[22] Li S, Zhang B, Zhang N. Network target for screening synergistic drug combinations with application to traditional Chinese medicine. BMC Syst Biol. 2011; Suppl 1:S10. 10.1186/1752-0509-5-S1-S10 PMC3121110 21689469

[23] Zhou W, Wang Y. A network-based analysis of the types of coronary artery disease from traditional Chinese medicine perspective: potential for therapeutics and drug discovery. J Ethnopharmacol. 2014; 151(1):66–77. 10.1016/j.jep.2013.11.007 24269247

[24] Li R, Ma T, Gu J, Liang X, Li S. Imbalanced network biomarkers for traditional Chinese medicine syndrome in gastritis patients. Sci Rep. 2013; 2013:1543. 10.1038/srep01543 PMC3607832 23529020

[25] Zhang B, Wang X, Li S. An integrative platform of TCM network pharmacology and its application on a herbal formula, Qing-Luo-Yin. Evid Based Complement Alternat Med. 2013; 2013:456747 10.1155/2013/456747 23653662 PMC3638581

[26] Li H, Zhao L, Zhang B, Jiang L, Wang X, Guo Y, et al A network pharmacology approach to determine active compounds and action mechanisms of Ge-Gen-Qin-Lian decoction for treatment of type 2 diabetes. Evid Based Complement and Alternat Med. 2014; 2014:495840. 24527048 10.1155/2014/495840 PMC3914348

[27] World Health Organization. The top 10 causes of death. [Updated May 2014; cited 10 Mar 2015]. http://www.who.int/mediacentre/factsheets/fs310/en/

[28] Rader DJ. New therapies for coronary artery disease: genetics provides a blueprint. Sci Transl Med. 2014; 6:239ps4 10.1126/scitranslmed.3008535 24898745

[29] Ren Y, Zhang M, Chen K, You S, Li J, Guo L, et al Clinical and epidemiological investigation of TCM syndromes of patients with coronary heart disease in China. Evid Based Complement Alternat Med. 2012; 2012:714517 10.1155/2012/714517 22536290 PMC3318902

[30] Gao ZY, Xu H, Shi DZ, Wen C, Liu BY. Analysis on outcome of 5284 patients with coronary artery disease: the role of integrative medicine. J Ethnopharmacol. 2012; 141(2):578–583. 10.1016/j.jep.2011.08.071 21924336

[31] Mao JY, Niu ZC, Zhang BL. Literature analysis of studies on the TCM syndromes of coronary heart disease in the recent 40 years. J Tradit Chin Med. 2011; 52(11):958–961.

[32] Maciocia Giovanni. The practice of Chinese medicine: the treatment of disease with acupuncture and Chinese herbs (2nd). Churchill Livingstone press; 1994.

[33] Bi Y, Mao J, Wang X. Expert survey on application of traditional Chinese medicine in different clinical classifications of coronary heart disease. World Sci Tech (Modern Tradit Chin Med Materia Medica). 2013; 15(05): 804–806.

[34] Huang Q, Qiao X, Xu X. Potential synergism and inhibitors to multiple target enzymes of Xuefu Zhuyu decoction in cardiac disease therapeutics: a computational approach. Bioorg Med Chem Lett. 2007; 17(6):1779–1783. 10.1016/j.bmcl.2006.12.078 17236764

[35] Lee JJ, Hsu WH, Yen TL, Chang NC, Luo YJ, et al Traditional Chinese medicine, Xue-Fu-Zhu-Yu decoction, potentiates tissue plasminogen activator against thromboembolic stroke in rats. J Ethnopharmol. 2011, 134(3):824–830. 10.1016/j.jep.2011.01.033 21315142

[36] Song X, Wang J, Wang P, Tian N, Yang M, Kong L. 1H NMR-based metabolomics approach to evaluate the effect of Xue-Fu-Zhu-Yu decoction on hyperlipidemia rats induced by high-fat diet. J Pharm Biomed Anal. 2013; 78–79:202–210. 10.1016/j.jpba.2013.02.014 23501440

[37] Song J, Chen WY, Wu LY, Zheng LP, Lin W, Gao D, et al A microarray analysis of angiogenesis modulation effect of Xuefu Zhuyu decoction on endothelial cells. Chin J Integr Med. 2012; 18(7):502–506. 10.1007/s11655-012-1143-6 22772912 PMC3698960

[38] Zhang HM, Tang DL, Tong L, Sun MJ, Sui Y, Zhu HY, et al Gualou Xiebai Banxia Decoction Inhibits NF-kappa B-dependent Inflammation in Myocardial Ischemia- reperfusion Injury in Rats. J Tradit Chin Med. 2011; 31(4):338–343. 22462242 10.1016/s0254-6272(12)60015-6

[39] Guo SW, Wang GH, Shi KH, Liu JQ. Study on reduction of pulmonary hypertension of Gualou Xiebai Banxia decoction. Chin J Integr Med.1997; 17:23–25.

[40] Li XH, Zhang BT, Yu R. Protective effect of Gualou Xiebai Banxia decoction on acute myocardial ischemia in rats. J Trad Chin Med Univ Hunan. 2009; 29(1):19–22.

[41] Zhuang Y. Systematic review of associated prescriptions of Gualou and Xiebai on unstable angina pectoris. J SD Univ TCM. 2014 38(4):316–319.

[42] Ru J, Li P, Wang J, Zhou W, Li B, Huang C, et al TCMSP: a database of systems pharmacology for drug discovery from herbal medicines. J. Cheminformatics, 2014, 6(1):13. 10.1186/1758-2946-6-13 PMC4001360 24735618

[43] Xue R, Fang Z, Zhang M, Yi Z, Wen C, Shi T. TCMID: Traditional Chinese Medicine integrative database for herb molecular mechanism analysis. Nucleic Acids Res. 2013; 41(Database issue):D1089–1095. 10.1093/nar/gks1100 23203875 PMC3531123

[44] Fihn SD, Gardin JM, Abrams J, Berra K, Blanenship JC, Dallas AP, et al 2012 ACCF/AHA/ACP/AATS/PCNA/SCAI/STS Guideline for the diagnosis and management of patients with stable ischemic heart disease: a report of the American College of Cardiology Foundation/American Heart Association Task Force on Practice Guidelines, and the American College of Physicians, American Association for Thoracic Surgery, Preventive Cardiovascular Nurses Association, Society for Cardiovascular Angiography and Interventions, and Society of Thoracic Surgeons. J Am Coll Cardiol. 2012; 60(24):e44–e164. 10.1016/j.jacc.2012.07.013 23182125

[45] Roger VL, Go AS, Lloyd-Jones DM, Benjamin EJ, Berry JD, Borden WB, et al Executive summary: heart disease and stroke statistics—2012 update: a report from the American Heart Association. Circulation. 2012; 125(1):188–197. 10.1161/CIR.0b013e3182456d46 22215894

[46] Wishart DS, Knox C, Guo AC, Cheng D, Shrivastava S, Tzur D, et al DrugBank: a knowledgebase for drugs, drug actions and drug targets. Nucleic Acids Res. 2008; 36(Database issue):D901–906. 10.1093/nar/gkm958 18048412 PMC2238889

[47] Kanehisa M, Goto S, Sato Y, Furumichi M, Tanabe M. KEGG for integration and interpretation of large-scale molecular data sets. Nucleic Acids Res. 2012; 40(Database issue):D109–114. 10.1093/nar/gkr988 22080510 PMC3245020

[48] Levandowsky M, Winter D. Distance between sets. Nature. 1971; 234(5323):34–35.

[49] Fliri AF, Loging WT, Volkmann RA. Cause-effect relationships in medicine: a protein network perspective. Trends Pharmacol Sci. 2010; 31(11):547–555. 10.1016/j.tips.2010.07.005 20810173

[50] Araujo RP, Doran C, Liotta LA, Petricoin EF. Network-targeted combination therapy: a new concept in cancer treatment. Drug Discov Today Ther Strateg. 2004; 1(4):425–433.

[51] Yang M, Chen JL, Xu LW, Ji G. Navigating traditional Chinese medicine network pharmacology and computational tools. Evid Based Complement Alternat Med. 2013; 2013:731969 10.1155/2013/731969 23983798 PMC3747450

[52] Bastian M, Heymann S, Jacomy M. Gephi: an open source software for exploring and manipulating networks. ICWSM. 2009; 8:361–362

[53] Hu Y, Scherngell T, Man SN, Wang Y. Is the United States still dominant in the global pharmaceutical innovation network? PLoS One. 2013; 8:e77247 10.1371/journal.pone.0077247 24223710 PMC3818371

[54] Lin JR. Social network analysis: theory, method and application. 1. Beijing: Beijing Normal University Press; 2009.

[55] Newman M. The structure of scientific collaboration networks. Proc Natl Acad Sci USA. 2004; 101:5200–5205. 14745042 10.1073/pnas.0307545100 PMC387296

[56] Newman M. A measure of betweenness centrality based on random walks. Social networks. 2005; 27(1): 39–54.

[57] Newman M. Who is the best connected scientist? A study of scientific coauthorship networks: complex networks. Berlin: Springer; 2004.

[58] Ulrik B. A faster algorithm for betweenness centrality. J Math Sociol. 2001; 25(2):163–177.

[59] Ott RL, Longnecker M. An introduction to statistical methods and data analysis. 7th ed. Boston: Cengage learning; 2015.

[60] Manimaran P, Hegde SR, Mande SC. Prediction of conditional gene essentiality through graph theoretical analysis of genome-wide functional linkages. Mol Biosyst. 2009; 5(12): 1936–42. 10.1039/B905264j 19763329

[61] Nie PH, Zhang L, Zhang WH, Rong WF, Zhi JM. The effects of hydroxysafflor yellow A on blood pressure and cardiac function. J Ethnopharmacol. 2012; 139(3):746–750. 10.1016/j.jep.2011.11.054 22197825

[62] Sun X, Wei X, Qu S, Zhao Y, Zhang X. Hydroxysafflor Yellow A suppresses thrombin generation and inflammatory responses following focal cerebral ischemia-reperfusion in rats. Bioorg Med Chem Lett. 2010; 20(14):4120–4124. 10.1016/j.bmcl.2010.05.076 20542424

[63] Yang JY, Chen HH, Wu J, Gong SX, Chen CQ, et al Advances in studies on pharmacological functions of ligustilide and their mechanisms. Chin Herb Med. 2012; 4 (1):26–32.

[64] Takahashi T, Takasuka N, Iigo M, Baba M, Nishino H, Tsuda H, et al Isoliquiritigenin, a flavonoid from licorice, reduces prostaglandin E2 and nitric oxide, causes apoptosis, and suppresses aberrant crypt foci development. Cancer Sci. 2004; 95(5):448–453. 15132774 10.1111/j.1349-7006.2004.tb03230.x PMC11158064

[65] Zhou H, Yang X, Wang NL, Zhang YO, Cai GP. Macrostemonoside A promotes visfatin expression in 3T3-L1 cells. Biol Pharm Bull. 2007; 30(2):279–283. 17268065 10.1248/bpb.30.279

[66] Xie W, Zhang Y, Wang N, Zhou H, Du L, Ma X, et al Novel effects of macrostemonoside A, a compound from Allium Macrostemon Bung, on hyperglycemia, hyperlipidemia, and visceral obesity in high-fat diet-fed C57BL/6 mice. Eur J Pharmacol. 2008; 599(1–3):159–165. 10.1016/j.ejphar.2008.09.042 18930725

[67] Anusree SS, Nisha VM, Priyanka A, Raghu KG. Insulin resistance by TNF-α is associated with mitochondrial dysfunction in 3T3-L1 adipocytes and is ameliorated by punicic acid, a PPARγ agonist. Mol Cell Endocrinol. 2015; 413:120–128. 10.1016/j.mce.2015.06.018 26116231

[68] Borges FR, Silva MD, Córdova MM, Schambach TR, Pizzolatti MG, Santos AR. Anti- inflammatory action of hydroalcoholic extract, dichloromethane fraction and steroid α-spinasterol from Polygala Sabulosa in LPS-induced peritonitis in mice. J Ethnopharmacol. 2014; 151(1):144–150. 10.1016/j.jep.2013.10.009 24161429

[69] Fulton D, Gratton JP, McCabe TJ, Fontana J, Fujio Y, Walsh K, et al Regulation of endothelium-derived nitric oxide production by the protein kinase Akt. Nature. 1999; 399(6736):597–601. 10.1038/21218 10376602 PMC3637917

[70] Zimmermann K, Opitz N, Dedio J, Renne C, Muller-Esteri W, Oess S. NOSTRIN: a protein modulating nitric oxide release and subcellular distribution of endothelial nitric oxide synthase. Proc Natl Acad Sci USA. 2002; 99(26):17167–1772. 10.1073/pnas.252345399 12446846 PMC139423

[71] Dedio J, König P, Wohlfart P, Kummer W, Müller-Esterl W. NOSIP, a novel modulator of endothelial nitric oxide synthase activity. FASEB J. 2001; 15(1):79–89. 10.1096/fj.00-0078com 11149895

[72] Elchebly M, Payette P, Michaliszyn E, Cromlish W, Collins S, Loy AL, et al Increased insulin sensitivity and obesity resistance in mice lacking the protein tyrosine phosphatase-1B gene. Science. 1999; 283(5407):1544–1548. 10066179 10.1126/science.283.5407.1544

[73] Belin de Chantemèle EJ, Muta K, Mintz J, Tremblay ML, Marrero MB, Fulton DJ, et al Protein tyrosine phosphatase 1B, a major regulator of leptin-mediated control of cardiovascular function. Circulation. 2009; 120(9):753–763. 10.1161/CIRCULATIONAHA.109.853077 19687357 PMC2736363

[74] Gambaryan S, Kobsar A, Rukoyatkina N, Herterich S, Geiger J, Smolenski A, et al Thrombin and collagen induce a feedback inhibitory signaling pathway in platelets involving dissociation of the catalytic subunit of protein kinase A from an NFkappaB-IkappaB complex. J Biol Chem. 2010; 285(24):18352–18363. 10.1074/jbc.M109.077602 20356841 PMC2881761

[75] Lange M, Fujikawa T, Koulova A, Kang S, Griffin MJ, Lassaletta AD, et al Arterial territory-specific phosphorylated retinoblastoma protein species and CDK2 promote differences in the vascular smooth muscle cell response to mitogens. Cell Cycle. 2014;13(2):315–323. 10.4161/cc.27056 24240190 PMC3906247

[76] Kaludercic N, Mialet-Perez J, Paolocci N, Parini A, Di Lisa F. Monoamine oxidases as sources of oxidants in the heart. J Mol Cell Cardiol. 2014; 73:34–42. 10.1016/j.yjmcc.2013.12.032 24412580 PMC4048760

[77] Tsutsumi S, Zhang X, Takata K, Takahashi K, Karas RH, Kurachi H, et al Differential regulation of the inducible nitric oxide synthase gene by estrogen receptors 1 and 2. J Endocrinol. 2008; 199(2):267–273. 10.1677/JOE-07-0292 18753331 PMC2773690

[78] Lawlor DA, Nordestgaard BG, Benn M, Zuccolo L, Tybjaerg-Hansen A, Davey Smith G. Exploring causal associations between alcohol and coronary heart disease risk factors: findings from a Mendelian randomization study in the Copenhagen General Population Study. Eur Heart J. 2013; 34(32):2519–2528. 10.1093/eurheartj/eht081 23492672

[79] Keith RJ, Haberzettl P, Vladykovskaya E, Hill BG, Kaiserova K, Srivastava S, et al Aldose reductase decreases endoplasmic reticulum stress in ischemic hearts. Chem Biol Interact. 2009; 178(1–3):242–249. 10.1016/j.cbi.2008.10.055 19041636 PMC3178409

