# Supplementary material for: Natural formulas and the nature of formulas: Exploring potential therapeutic targets based on traditional Chinese herbal formulas
Source: PLoS One. 2017 Feb 9;12(2):e0171628. doi: 10.1371/journal.pone.0171628 (PMC5300118; doi:10.1371/journal.pone.0171628)
Supplement: S1 Table — (DOCX) [file pone.0171628.s001.docx]

S1 Table. Chemical compounds in herbal medicines of XZD

| MOL_ID | Chemical Compounds | M.F. | M.W. | CAS | Pubchem_CID | Herbal Medicines |
| --- | --- | --- | --- | --- | --- | --- |
| MOL000003 | D-mannitol | C6H14O6 | 182.2 | 133-43-7 | 6251 | Radix Rehmanniae |
| MOL000006 | luteolin | C15H10O6 | 286.25 | 491-70-3 | 5280445 | Flos Carthami, Radix Platycodonis |
| MOL000008 | apigenin | C15H10O5 | 270.25 | 461015-54-3 | 5280443 | Flos Carthami |
| MOL000012 | Arachic acid | C20H40O2 | 312.6 | 506-30-9 | 10467 | Radix et Rhizoma Glycyrrhizae, Flos Carthami, Radix Rehmanniae |
| MOL000018 | (+/-)-Isoborneol | C10H18O | 154.28 | 124-76-5 | 6321405 | Radix Bupleuri |
| MOL000019 | D-Camphene | C10H16 | 136.26 | 5794/3/6 | 92221 | Radix Bupleuri, Rhizoma Chuanxiong |
| MOL000023 | Hemo-sol | C10H16 | 136.26 | 5989-27-5 | 440917 | Radix Bupleuri, Radix Angelicae Sinensis |
| MOL000024 | alpha-humulene | C15H24 | 204.39 | 19132-75-3 | 5281520 | Rhizoma Chuanxiong, Flos Carthami, |
| MOL000027 | alpha-Curcumene | C15H22 | 202.37 | 4176-17-4 | 442360 | Radix Bupleuri, Rhizoma Chuanxiong |
| MOL000029 | beta-Humulene | C15H24 | 204.39 | 116-04-1 | 5318102 | Radix Bupleuri |
| MOL000032 | beta-Eudesmol | C15H26O | 222.41 | 473-15-4 | 91457 | Radix Bupleuri |
| MOL000034 | 2-[(1R,3S,4S)-3-isopropenyl-4-methyl-4-vinylcyclohexyl] propan-2-ol | C15H26O | 222.41 | 639-99-6 | 92138 | Rhizoma Chuanxiong |
| MOL000035 | beta-Selinene | C15H24 | 204.39 | 17066-67-0 | 442393 | Rhizoma Chuanxiong, Radix Angelicae Sinensis |
| MOL000036 | beta-caryophyllene | C15H24 | 204.39 | 8007-38-3 | 5281515 | Radix Bupleuri, Flos Carthami, |
| MOL000040 | Scopoletol | C10H8O4 | 192.18 | 92-61-5 | 5280460 | Radix Bupleuri, Radix Angelicae Sinensis, Radix et Rhizoma Glycyrrhizae |
| MOL000055 | L-Lysine | C6H14N2O2 | 146.22 | 56-87-1 | 5962 | Flos Carthami |
| MOL000057 | Isobutyl phthalate | C16H22O4 | 278.38 | 84-69-5 | 6782 | Radix Bupleuri, Radix et Rhizoma Glycyrrhizae, Radix Achyranthis Bidentatae |
| MOL000067 | L-(+)-Valine | C5H11NO2 | 117.17 | 16872-32-5 | 6971018 | Radix Platycodonis |
| MOL000068 | L-Ile | C6H13NO2 | 131.2 | 73-32-5 | 7043901 | Radix Platycodonis |
| MOL000069 | palmitic acid | C16H32O2 | 256.48 | 67701-02-4 | 985 | Radix Bupleuri, Radix Paeoniae Rubra, Rhizoma Chuanxiong, Radix Angelicae Sinensis, Flos Carthami, Radix Platycodonis, Radix Achyranthis Bidentatae |
| MOL000084 | beta-daucosterol | C35H60O6 | 576.95 | Not Available | 5742590 | Radix Achyranthis Bidentatae |
| MOL000085 | beta-daucosterol_qt | Not Available | 414.79 | Not Available | Not Available | Radix Achyranthis Bidentatae |
| MOL000098 | quercetin | C15H10O7 | 302.25 | 73123-10-1 | 5280343 | Radix Bupleuri, Radix et Rhizoma Glycyrrhizae, Flos Carthami, Radix Achyranthis Bidentatae |
| MOL000103 | 4-oxoniobenzoate | C7H6O3 | 138.13 | 99-96-7 | 3702506 | Rhizoma Chuanxiong |
| MOL000105 | protocatechuic acid | C7H6O4 | 154.13 | 99-50-3 | 72 | Radix et Rhizoma Glycyrrhizae |
| MOL000114 | vanillic acid | C8H8O4 | 168.16 | 121-34-6 | 8468 | Radix Paeoniae Rubra, Rhizoma Chuanxiong |
| MOL000116 | Nonanal | C9H18O | 142.27 | 75718-12-6 | 31289 | Rhizoma Chuanxiong, Radix Angelicae Sinensis, Flos Carthami |
| MOL000117 | Cymol | C10H14 | 134.24 | 4939-75-7 | 7463 | Radix Bupleuri, Rhizoma Chuanxiong, Radix Angelicae Sinensis |
| MOL000118 | (L)-alpha-Terpineol | C10H18O | 154.28 | 10482-56-1 | 443162 | Radix Bupleuri, Rhizoma Chuanxiong, Radix et Rhizoma Glycyrrhizae |
| MOL000120 | Trans-2-Decenal | C10H18O | 154.28 | 3913-81-3 | 5283345 | Radix Bupleuri |
| MOL000121 | Decanal | C10H20O | 156.3 | 112-31-2 | 8175 | Radix Angelicae Sinensis , Flos Carthami |
| MOL000122 | 1,8-cineole | C10H18O | 154.28 | 470-82-6 | 2758 | Radix Bupleuri, Rhizoma Chuanxiong |
| MOL000123 | geraniol | C10H18O | 154.28 | 624-15-7 | 637566 | Radix Bupleuri |
| MOL000125 | (-)-alpha-Pinene | C10H16 | 136.26 | 7785-26-4 | 440968 | Rhizoma Chuanxiong, Radix Angelicae Sinensis |
| MOL000126 | (-)-nopinene | C10H16 | 136.26 | 18172-67-3 | 440967 | Radix Bupleuri, Rhizoma Chuanxiong Fructus Aurantii, |
| MOL000128 | Nerylacetate | C12H20O2 | 196.32 | 33843-18-4 | 1549026 | Radix Bupleuri |
| MOL000131 | Linoleic acid | C18H32O2 | 280.5 | 2197-37-7 | 5280450 | Radix Bupleuri, Radix Paeoniae Rubra, Rhizoma Chuanxiong, Flos Carthami, Semen Persicae, Radix Rehmanniae |
| MOL000162 | beta-Chamigrene | C15H24 | 204.39 | 18431-82-8 | 442353 | Radix Angelicae Sinensis |
| MOL000165 | 2-[(2S,5S,6S)-6,10-dimethylspiro[4.5]dec-9-en-2-yl] propan-2-ol | C15H26O | 222.41 | 1460-73-7 | 10353528 | Rhizoma Chuanxiong |
| MOL000169 | alpha-Guaiene | C15H24 | 204.39 | 654486 | 5317844 | Radix Bupleuri |
| MOL000172 | Furol | C5H4O2 | 96.09 | 1998/1/1 | 7362 | Rhizoma Chuanxiong, Radix Achyranthis Bidentatae |
| MOL000173 | wogonin | C16H12O5 | 284.28 | 632-85-9 | 5281703 | Radix Achyranthis Bidentatae |
| MOL000193 | (Z)-caryophyllene | C15H24 | 204.39 | 87-44-5 | 6429301 | Radix Bupleuri |
| MOL000196 | L-Bornyl acetate | C12H20O2 | 196.32 | 6626-35-3 | 93009 | Radix Bupleuri, Rhizoma Chuanxiong |
| MOL000197 | Myrcene | C10H16 | 136.26 | 123-35-3 | 31253 | Radix Bupleuri, Rhizoma Chuanxiong, Radix Angelicae Sinensis Fructus Aurantii |
| MOL000198 | (R)-linalool | C10H18O | 154.28 | 126-91-0 | 443158 | Radix Bupleuri, Rhizoma Chuanxiong |
| MOL000199 | Safrol | C10H10O2 | 162.2 | 94-59-7 | 5144 | Radix Angelicae Sinensis |
| MOL000200 | (S)-(+)-alpha-Phellandrene | C10H16 | 136.26 | 2243-33-6 | 443160 | Rhizoma Chuanxiong |
| MOL000202 | Moslene | C10H16 | 136.26 | 99-85-4 | 7461 | Rhizoma Chuanxiong, Radix Angelicae Sinensis |
| MOL000204 | -cis-.beta.-Elemene diastereomer | C15H24 | 204.39 | 515-13-9 | 6431152 | Rhizoma Chuanxiong |
| MOL000206 | isoeugenol | C10H12O2 | 164.22 | 5932-68-3 | 853433 | Radix Angelicae Sinensis |
| MOL000207 | Methyleugenol | C11H14O2 | 178.25 | 6380-24-1 | 7127 | Radix Bupleuri, Rhizoma Chuanxiong |
| MOL000208 | ()-Aromadendrene | C15H24 | 204.39 | 489-39-4 | 11095734 | Rhizoma Chuanxiong |
| MOL000211 | Mairin | C30H48O3 | 456.78 | 472-15-1 | 64971 | Radix et Rhizoma Glycyrrhizae |
| MOL000219 | BOX | C7H6O2 | 121.12 | 65-85-0 | 20144841 | Radix Paeoniae Rubra |
| MOL000222 | terephthaldehyde | C16H18O9 | 354.34 | 623-27-8 | 7067335 | Flos Carthami |
| MOL000223 | caffeic acid | C9H8O4 | 180.17 | 71693-97-5 | 689043 | Rhizoma Chuanxiong, Flos Carthami, |
| MOL000232 | (+)-alpha-Terpineol | C10H18O | 154.28 | 7785-53-7 | 442501 | Radix Angelicae Sinensis |
| MOL000233 | delta-Terpineol | C10H18O | 154.28 | 7299-42-5 | 81722 | Radix Bupleuri |
| MOL000234 | L-Limonen | C10H16 | 136.26 | 5989-54-8 | 439250 | Radix Bupleuri, Rhizoma Chuanxiong Fructus Aurantii |
| MOL000239 | Jaranol | C17H14O6 | 314.31 | 3301-49-3 | 5318869 | Radix et Rhizoma Glycyrrhizae |
| MOL000244 | ()-Borneol | C10H18O | 154.28 | 95-92-1 | 6552009 | Radix Bupleuri |
| MOL000247 | (Z,Z)-farnesol | C15H26O | 222.41 | 4602-84-0 | 1549107 | Radix Bupleuri |
| MOL000249 | Methylcinnamate | C10H10O2 | 162.2 | 1754-62-7 | 637520 | Flos Carthami |
| MOL000254 | eugenol | C10H12O2 | 164.22 | 97-53-0 | 3314 | Radix Bupleuri |
| MOL000259 | o-Thymol | C10H14O | 150.24 | 499-75-2 | 10364 | Radix Bupleuri, Radix Angelicae Sinensis |
| MOL000263 | oleanolic acid | C30H48O3 | 456.78 | 508-02-1 | 10494 | Radix et Rhizoma Glycyrrhizae, Radix Achyranthis Bidentatae |
| MOL000264 | Tereben | C10H16 | 136.26 | 69073-38-7 | 11463 | Rhizoma Chuanxiong |
| MOL000268 | (1S,5S)-1-isopropyl-4-methylenebicyclo[3.1.0] hexane | C10H16 | 136.26 | 87-41-5 | 11051711 | Rhizoma Chuanxiong |
| MOL000270 | CHEBI:7 | C10H16 | 136.26 | 498-15-7 | 443156 | Radix Bupleuri, Rhizoma Chuanxiong, Radix Angelicae Sinensis |
| MOL000271 | l-carvone | C10H14O | 150.24 | 6485-40-1 | 439570 | Radix Bupleuri |
| MOL000284 | L-uridine | C9H12N2O6 | 244.23 | 26287-69-4 | 40428662 | Radix Rehmanniae |
| MOL000286 | β-amyrin acetate | C32H52O2 | 468.84 | 1616-93-9 | 51398127 | Flos Carthami |
| MOL000295 | Stigmasterin | C29H48O | 412.691 | 83-48-7 | 5280794 | Semen Persicae |
| MOL000296 | hederagenin | C30H48O4 | 472.7 | 465-99-6 | 73299 | Semen Persicae |
| MOL000302 | Undekansaeure | C11H22O2 | 186.33 | 112-37-8 | 8180 | Radix Bupleuri, Rhizoma Chuanxiong |
| MOL000303 | caprylic acid | C8H16O2 | 144.24 | 68937-74-6 | 379 | Radix Bupleuri, Radix Achyranthis Bidentatae, |
| MOL000305 | lauric acid | C12H24O2 | 200.36 | 8045-27-0 | 3893 | Radix Bupleuri, Flos Carthami, Radix Rehmanniae |
| MOL000340 | Hydrocinnamic acid | C9H10O2 | 150.19 | 501-52-0 | 107 | Flos Carthami |
| MOL000346 | succinic acid | C4H6O4 | 118.1 | 110-15-6 | 21952380 | Radix Angelicae Sinensis , Flos Carthami, Radix Achyranthis Bidentatae, Radix Rehmanniae |
| MOL000347 | Syrigin | C17H24O9 | 372.41 | 118-34-3 | 5316860 | Flos Carthami |
| MOL000348 | 4-[(Z)-3-hydroxyprop-1-enyl]-2,6-dimethoxyphenol | C11H14O4 | 210.25 | 118-34-3 | 10130521 | Flos Carthami |
| MOL000354 | isorhamnetin | C16H12O7 | 316.28 | 480-19-3 | 5281654 | Radix Bupleuri, Radix et Rhizoma Glycyrrhizae |
| MOL000356 | lupeol | C30H50O | 426.8 | 545-47-1 | 259846 | Flos Carthami |
| MOL000357 | Sitogluside | C35H60O6 | 576.95 | 474-58-8 | 5742590 | Radix Paeoniae Rubra, Rhizoma Chuanxiong, Radix Angelicae Sinensis, Flos Carthami, Radix Achyranthis Bidentatae, Radix Rehmanniae |
| MOL000358 | beta-sitosterol | C29H50O | 414.79 | 83-46-5 | 222284 | Radix Paeoniae Rubra, Radix Angelicae Sinensis, Flos Carthami, Radix Achyranthis Bidentatae, Semen Persicae, Fructus Aurantii |
| MOL000359 | sitosterol | C29H50O | 414.79 | 149-91-7 | 12303645 | Radix Paeoniae Rubra, Rhizoma Chuanxiong, Radix et Rhizoma Glycyrrhizae, Radix Rehmanniae |
| MOL000360 | Ferulic acid | C10H10O4 | 194.2 | 537-98-4 | 445858 | Radix Angelicae Sinensis, Flos Carthami, Radix Rehmanniae |
| MOL000361 | Amyrin | C30H50O | 426.8 | 559-70-6 | 73145 | Radix Paeoniae Rubra |
| MOL000388 | gamma-aminobutyric acid | C4H9NO2 | 103.14 | 28805-76-7 | 6992099 | Radix Rehmanniae |
| MOL000389 | FERULIC ACID (CIS) | C10H10O4 | 194.2 | 1014-83-1 | 1548883 | Radix Angelicae Sinensis |
| MOL000391 | Ononin | C22H22O9 | 430.44 | 486-62-4 | 442813 | Radix et Rhizoma Glycyrrhizae |
| MOL000392 | formononetin | C16H12O4 | 268.28 | 485-72-3 | 5280378 | Radix et Rhizoma Glycyrrhizae |
| MOL000396 | (+)-Syringaresinol | C22H26O8 | 418.48 | 21453-69-0 | 443023 | Flos Carthami |
| MOL000397 | cis-p-Coumarate | C9H8O3 | 164.17 | 4501-31-9 | 1549106 | Semen Persicae |
| MOL000399 | Docosanoate | C22H44O2 | 340.66 | 16529-65-0 | 8215 | Radix Rehmanniae |
| MOL000414 | Cis-caffeate | C9H8O4 | 180.17 | 331-39-5 | 1549111 | Radix Rehmanniae |
| MOL000415 | rutin | C27H30O16 | 610.57 | 153-18-4 | 5280805 | Radix Bupleuri, Radix et Rhizoma Glycyrrhizae, Flos Carthami, Radix Achyranthis Bidentatae |
| MOL000417 | Calycosin | C16H12O5 | 284.28 | 20575-57-9 | 5280448 | Radix et Rhizoma Glycyrrhizae |
| MOL000421 | nicotinic acid | C6H5NO2 | 123.12 | 123574-58-3 | 938 | Radix Angelicae Sinensis, Radix Platycodonis |
| MOL000422 | kaempferol | C15H10O6 | 286.25 | 520-18-3 | 5280863 | Radix Bupleuri, Radix et Rhizoma Glycyrrhizae, Flos Carthami, Radix Achyranthis Bidentatae, |
| MOL000430 | betaine | C5H12NO2+ | 117.17 | 107-43-7 | 248 | Radix Achyranthis Bidentatae |
| MOL000431 | coumarin | C9H6O2 | 146.15 | 91-64-5 | 323 | Radix Bupleuri |
| MOL000432 | linolenic acid | C18H30O2 | 278.48 | 60-33-3 | 5280934 | Flos Carthami |
| MOL000433 | Folic acid | C19H19N7O6 | 441.45 | 33609-88-0 | 6037 | Rhizoma Chuanxiong |
| MOL000437 | Hirsutrin | C21H20O12 | 464.41 | 21637-25-2 | 5280804 | Radix Bupleuri, Radix et Rhizoma Glycyrrhizae |
| MOL000449 | Stigmasterol | C29H48O | 412.77 | 83-48-7 | 5280794 | Radix Bupleuri, Radix Paeoniae Rubra, Radix Angelicae Sinensis, Flos Carthami, Radix Achyranthis Bidentatae, Radix Rehmanniae |
| MOL000459 | CHEBI:39932 | C8H16O | 128.24 | 3391-86-4 | 6992244 | Radix Bupleuri |
| MOL000463 | Epi-Friedelanol | C30H52O | 428.82 | 16844-71-6 | 119242 | Radix Paeoniae Rubra |
| MOL000467 | Castanin | C17H14O5 | 298.31 | 550-79-8 | 5281704 | Radix et Rhizoma Glycyrrhizae |
| MOL000475 | Trans-anethole | C10H12O | 148.22 | 4180-23-8 | 637563 | Radix et Rhizoma Glycyrrhizae |
| MOL000478 | Eucarvone | C10H14O | 150.24 | 503-93-5 | 136330 | Radix Angelicae Sinensis |
| MOL000479 | Farnesene | C15H24 | 204.39 | 18452-58-9 | 5281517 | Radix Bupleuri, Radix Angelicae Sinensis, Flos Carthami |
| MOL000486 | Prunetin | C16H12O5 | 284.28 | 552-59-0 | 5281804 | Radix et Rhizoma Glycyrrhizae |
| MOL000489 | (1S,4aR,8aR)-1-isopropyl-7-methyl-4-methylene-2,3,4a,5,6,8a-hexahydro-1H-naphthalene | C15H24 | 204.39 | 1460-97-5 | 6432404 | Radix Angelicae Sinensis |
| MOL000490 | petunidin | C16H14O7 | 317.29 | 1429-30-7 | 73386 | Radix Bupleuri |
| MOL000492 | (+)-catechin | C15H14O6 | 290.29 | 154-23-4 | 9064 | Radix Paeoniae Rubra |
| MOL000493 | campesterol | C28H48O | 400.76 | 474-62-4 | 12358798 | Semen Persicae, Radix Rehmanniae |
| MOL000497 | licochalcone a | C21H22O4 | 338.43 | 58749-22-7 | 5318998 | Radix et Rhizoma Glycyrrhizae |
| MOL000500 | Vestitol | C16H16O4 | 272.32 | 20879-05-4 | 177149 | Radix et Rhizoma Glycyrrhizae |
| MOL000508 | Friedelin | C30H50O | 426.8 | 559-74-0 | 91472 | Radix Paeoniae Rubra |
| MOL000511 | ursolic acid | C30H48O3 | 456.78 | 77-52-1 | 64945 | Radix et Rhizoma Glycyrrhizae |
| MOL000513 | 3,4,5-trihydroxybenzoic acid | C7H6O5 | 170.13 | 149-91-7 | 370 | Radix Paeoniae Rubra |
| MOL000514 | Nonacosane | C29H60 | 408.89 | 630-03-5 | 12409 | Flos Carthami |
| MOL000561 | Astragalin | C21H20O11 | 448.41 | 480-10-4 | 5282102 | Radix et Rhizoma Glycyrrhizae, Flos Carthami, Radix Achyranthis Bidentatae |
| MOL000579 | hydroquinone | C6H6O2 | 110.12 | 57534-13-1 | 785 | Radix Paeoniae Rubra |
| MOL000608 | ()-Terpinen-4-ol | C10H18O | 154.28 | 2438-10-0 | 2724161 | Rhizoma Chuanxiong, Flos Carthami |
| MOL000612 | (-)-alpha-cedrene | Not Available | 204.39 | 69-61-4 | Not Available | Flos Carthami |
| MOL000613 | 8-isopropylidene-1,5-dimethylcyclodeca-1,5-diene | C15H24 | 204.39 | 15423-57-1 | 6370843 | Fructus Aurantii |
| MOL000635 | vanillin | C8H8O3 | 152.16 | 121-33-5 | 1183 | Radix Bupleuri, Rhizoma Chuanxiong, Radix Angelicae Sinensis |
| MOL000666 | hexanal | C6H12O | 100.18 | 66-25-1 | 6184 | Radix Bupleuri, Rhizoma Chuanxiong, Radix Achyranthis Bidentatae, |
| MOL000668 | PENTYLFURAN | C9H14O | 138.23 | 64079-01-2 | 19602 | Radix Bupleuri, Rhizoma Chuanxiong, Radix et Rhizoma Glycyrrhizae |
| MOL000671 | ()-Menthol | C10H20O | 156.3 | 89-78-1 | 165675 | Radix et Rhizoma Glycyrrhizae |
| MOL000675 | oleic acid | C18H34O2 | 282.52 | 17156-84-2 | 445639 | Radix Bupleuri, Rhizoma Chuanxiong, Flos Carthami |
| MOL000676 | Dibutyl phthalate | C16H22O4 | 278.38 | 84-74-2 | 3026 | Radix Bupleuri, Radix et Rhizoma Glycyrrhizae, Radix Achyranthis Bidentatae |
| MOL000698 | (R)-(-)-alpha-Phellandrene | C10H16 | 136.26 | 4221-98-1 | 442482 | Rhizoma Chuanxiong |
| MOL000703 | 2-heptanone | C7H14O | 114.21 | 29299-43-2 | 8051 | Radix et Rhizoma Glycyrrhizae, Radix Achyranthis Bidentatae |
| MOL000708 | Benzaldehyde | C7H6O | 106.13 | 100-52-7 | 240 | Radix Bupleuri, Radix Achyranthis Bidentatae, |
| MOL000712 | o-Cymol | C10H14 | 134.24 | 1329-98-2 | 10703 | Rhizoma Chuanxiong |
| MOL000713 | (E)-oct-3-en-2-one | C8H14O | 126.22 | 1669-44-9 | 5363229 | Radix Achyranthis Bidentatae |
| MOL000714 | Hyacinthin | C8H8O | 120.16 | 122-78-1 | 998 | Flos Carthami |
| MOL000715 | l-Menthone | C10H18O | 154.28 | 21060-23-1 | 26447 | Radix Bupleuri |
| MOL000716 | trans-2-nonenal | C9H16O | 140.25 | 18829-56-6 | 5283335 | Radix Bupleuri |
| MOL000723 | trans-2,4-decadienal | C10H16O | 152.26 | 30551-18-9 | 5283349 | Radix Bupleuri, Flos Carthami |
| MOL000724 | Geranylacetone | C13H22O | 194.35 | 689-67-8 | 1549778 | Radix Bupleuri |
| MOL000748 | 5-Hydroxymethyl-2-furaldehyde | C6H6O3 | 126.12 | 67-47-0 | 237332 | Flos Carthami, Radix Achyranthis Bidentatae, Radix Rehmanniae |
| MOL000775 | Ethyl ethanoate | C4H8O2 | 88.12 | 141-78-6 | 8857 | Flos Carthami, Radix Achyranthis Bidentatae, |
| MOL000776 | Ethanol | C2H6O | 46.08 | 121182-78-3 | 702 | Radix Achyranthis Bidentatae |
| MOL000785 | palmatine | C21H22NO4+ | 352.44 | 3486-67-7 | 19009 | Radix Achyranthis Bidentatae |
| MOL000841 | raffinose | C18H32O16 | 504.5 | 17629-30-0 | 439242 | Radix Rehmanniae |
| MOL000842 | sucrose | C12H22O11 | 342.34 | 25702-74-3 | 5988 | Rhizoma Chuanxiong, Radix Rehmanniae |
| MOL000860 | stearic acid | C18H36O2 | 284.54 | 609343-71-7 | 5281 | Radix Bupleuri, Rhizoma Chuanxiong, Flos Carthami, Radix Platycodonis, Radix Achyranthis Bidentatae |
| MOL000865 | hexadecane | C16H34 | 226.5 | 544-76-3 | 11006 | Radix Achyranthis Bidentatae, |
| MOL000873 | Pimelic ketone | C6H10O | 98.16 | 11119-77-0 | 7967 | Radix Bupleuri |
| MOL000874 | paeonol | C9H10O3 | 166.19 | 552-41-0 | 11092 | Radix Paeoniae Rubra |
| MOL000878 | Farnesylacetone | C18H30O | 262.48 | 762-29-8 | 1711945 | Radix Bupleuri |
| MOL000879 | methyl palmitate | C17H341O2 | 270.51 | 112-39-0 | 8181 | Rhizoma Chuanxiong, Radix Achyranthis Bidentatae |
| MOL000886 | tetradecane | C14H30 | 198.44 | 90622-46-1 | 12389 | Radix Bupleuri, Rhizoma Chuanxiong, Radix Achyranthis Bidentatae |
| MOL000908 | beta-elemene | C15H24 | 204.39 | 33880-83-0 | 6918391 | Rhizoma Chuanxiong |
| MOL000911 | Terpilene | C10H16 | 136.26 | 99-86-5 | 7462 | Rhizoma Chuanxiong |
| MOL000922 | (R)-p-Menth-1-en-4-ol | C10H18O | 154.28 | 20126-76-5 | 5325830 | Radix Bupleuri, Rhizoma Chuanxiong |
| MOL000924 | 2-Undecanone | C11H22O | 170.33 | 112-12-9 | 8163 | Radix Bupleuri |
| MOL000937 | 58870_FLUKA | C15H24 | 204.39 | 95910-36-4 | 15431199 | Rhizoma Chuanxiong |
| MOL000953 | Cholesterin | C27H46O | 386.73 | 80356-14-5 | 5997 | Flos Carthami |
| MOL000971 | Ethylpalmitate | C18H36O2 | 284.54 | 628-97-7 | 12366 | Rhizoma Chuanxiong |
| MOL000974 | cuminal | C10H12O | 148.22 | 122-03-2 | 326 | Radix Bupleuri, Radix Angelicae Sinensis |
| MOL001002 | ellagic acid | C14H6O8 | 302.2 | 476-66-4 | 5281855 | Radix Paeoniae Rubra |
| MOL001006 | Chondrillasterol | C29H48O | 412.77 | 481-18-5 | 5283663 | Radix Achyranthis Bidentatae |
| MOL001055 | 5-isopropyl-2-methylbicyclo[3.1.0]hex-2-ene | C10H16 | 136.26 | 1406-51-5 | 637518 | Rhizoma Chuanxiong |
| MOL001097 | o-xylene | C8H10 | 106.18 | 95-47-6 | 7237 | Radix et Rhizoma Glycyrrhizae, Flos Carthami |
| MOL001098 | m-xylene | C8H10 | 106.18 | 108-38-3 | 7929 | Radix et Rhizoma Glycyrrhizae |
| MOL001099 | p-xylene | C8H10 | 106.18 | 106-42-3 | 7809 | Radix et Rhizoma Glycyrrhizae, Flos Carthami |
| MOL001109 | β-thujene | C10H16 | 136.26 | 28634-89-1 | 520384 | Radix Bupleuri |
| MOL001121 | (-)-Myrtenol | C10H16O | 152.26 | 19894-97-4 | 88301 | Radix Bupleuri |
| MOL001129 | l-Verbenone | C10H14O | 150.24 | 1196-01-6 | 92874 | Flos Carthami |
| MOL001179 | Alloaromadendrene | C15H24 | 204.39 | 464-43-7 | 10899740 | Radix Bupleuri |
| MOL001201 | (1R,5R,7S)-4,7-dimethyl-7-(4-methylpent-3-enyl) bicyclo[3.1.1]hept-3-ene | C15H24 | 204.39 | 79-92-5 | 13889654 | Rhizoma Chuanxiong |
| MOL001210 | (4S)-4-isopropylcyclohexene-1-carbaldehyde | C10H16O | 152.26 | 21391-98-0 | 11842593 | Rhizoma Chuanxiong |
| MOL001223 | (S)-2,2,3-Trimethylcyclopent-3-ene-1-acetaldehyde | C10H16O | 152.26 | 23727-15-3 | 90969 | Rhizoma Chuanxiong |
| MOL001224 | Tridecylene | C13H26 | 182.39 | 2437-56-1 | 17095 | Radix Angelicae Sinensis |
| MOL001273 | (+)-Verbenone | C10H14O | 150.24 | 80-57-9 | 65724 | Radix Bupleuri, Radix Angelicae Sinensis |
| MOL001283 | (3S,6E)-Nerolidol | C15H26O | 222.41 | 1119-38-6 | 5281525 | Radix Bupleuri |
| MOL001306 | o-Acetyl-p-cresol | C9H10O2 | 150.19 | 1450-72-2 | 15068 | Rhizoma Chuanxiong, Radix Angelicae Sinensis |
| MOL001315 | campesterol-3-O-β-D-glucopyranoside | C34H58O6 | 562.92 | 32214-82-7 | Not Available | Semen Persicae |
| MOL001316 | campesterol-3-O-β-D-glucopyranoside_qt | C28H48O | 400.76 | Not Available | 57511408 | Semen Persicae |
| MOL001320 | Amygdalin | C20H27NO11 | 457.48 | 29883-15-6 | 656516 | Semen Persicae |
| MOL001321 | (R)-mandelonitrile | C8H7NO | 133.16 | 10020-96-9 | 9548674 | Semen Persicae |
| MOL001323 | Citrostadienol | C30H50O | 426.8 | 474-40-8 | 9548595 | Semen Persicae |
| MOL001327 | 2,3-didehydro GA69 | Not Available | 330.41 | Not Available | Not Available | Semen Persicae |
| MOL001328 | 2,3-didehydro GA70 | Not Available | 330.41 | Not Available | Not Available | Semen Persicae |
| MOL001329 | 2,3-didehydro GA77 | Not Available | 346.41 | Not Available | Not Available | Semen Persicae |
| MOL001330 | 2,3-didehydro GA9 | Not Available | 314.41 | Not Available | Not Available | Semen Persicae |
| MOL001331 | Amygdalinic acid | C20H28O13 | 476.48 | 66427-92-7 | 6455326 | Semen Persicae |
| MOL001332 | (R)-(-)-Mandelic acid | C8H8O3 | 152.16 | 611-71-2 | 11914 | Semen Persicae |
| MOL001333 | 7-dehydroavenasterol | C29H48O | 412.77 | 23290-26-8 | 12795736 | Semen Persicae |
| MOL001335 | Benzyl alcohol | C7H8O | 108.15 | 1336-27-2 | 244 | Rhizoma Chuanxiong, Semen Persicae |
| MOL001338 | GA118 | Not Available | 348.43 | Not Available | Not Available | Semen Persicae |
| MOL001340 | Gibberellin A120 | C19H22O4 | 314.41 | Not Available | 25245018 | Semen Persicae |
| MOL001342 | GA121-isolactone | Not Available | 330.41 | Not Available | Not Available | Semen Persicae |
| MOL001344 | GA122-isolactone | Not Available | 330.41 | Not Available | Not Available | Semen Persicae |
| MOL001345 | Methyl-alpha-D-fructofuranoside | C7H14O6 | 194.21 | 15219-93-9 | 6325664 | Semen Persicae |
| MOL001347 | GA16 | Not Available | 348.43 | Not Available | Not Available | Semen Persicae |
| MOL001349 | 4a-formyl-7alpha-hydroxy-1-methyl-8-methylidene-4aalpha,4bbeta-gibbane-1alpha, 10beta-dicarboxylic acid | Not Available | 362.46 | 6980-44-5 | Not Available | Semen Persicae |
| MOL001351 | Gibberellin A44 | Not Available | 346.46 | 36434-15-8 | Not Available | Semen Persicae |
| MOL001352 | GA54 | Not Available | 348.43 | Not Available | Not Available | Semen Persicae |
| MOL001353 | GA60 | Not Available | 348.43 | Not Available | Not Available | Semen Persicae |
| MOL001355 | GA63 | Not Available | 348.43 | Not Available | Not Available | Semen Persicae |
| MOL001357 | GA69 | Not Available | 332.43 | Not Available | Not Available | Semen Persicae |
| MOL001358 | gibberellin 7 | Not Available | 330.41 | Not Available | Not Available | Semen Persicae |
| MOL001359 | GA70 | Not Available | 332.43 | Not Available | Not Available | Semen Persicae |
| MOL001360 | GA77 | Not Available | 348.43 | Not Available | Not Available | Semen Persicae |
| MOL001361 | GA87 | Not Available | 362.41 | Not Available | Not Available | Semen Persicae |
| MOL001362 | GA95 | Not Available | 330.41 | Not Available | Not Available | Semen Persicae |
| MOL001363 | GA97 | Not Available | 364.48 | Not Available | Not Available | Semen Persicae |
| MOL001366 | MNN | C8H7NO | 133.16 | 99-18-3 | 439767 | Semen Persicae |
| MOL001368 | 3-O-p-coumaroylquinic acid | C16H18O8 | 338.34 | 32451-86-8 | 9945785 | Semen Persicae |
| MOL001369 | Grandidentatin | C21H28O9 | 424.49 | 15732-48-6 | 52041673 | Semen Persicae |
| MOL001370 | [2-[(2S,3R,4S,5S,6R)-3,4,5-trihydroxy-6-(hydroxymethyl)oxan-2-yl]oxyphenyl]methyl (E)-3-(3,4-dihydroxyphenyl)prop-2-enoate | C22H24O10 | 448.46 | 99-17-2 | 23872119 | Semen Persicae |
| MOL001372 | Salireposide | C20H22O9 | 406.42 | 16955-55-8 | 117440 | Semen Persicae |
| MOL001373 | Salireposide_qt | C14H12O4 | 244.26 | 16955-55-8 | 12300041 | Semen Persicae |
| MOL001386 | Methyl laurate | C13H26O2 | 214.39 | 111-82-0 | 8139 | Radix Bupleuri |
| MOL001387 | (7aS)-4,4,7a-trimethyl-6,7-dihydro-5H-benzofuran-2-one | C11H16O2 | 180.27 | 81800-41-1 | 157995 | Flos Carthami |
| MOL001388 | (+)-Ledol | C15H26O | 222.41 | 577-27-5 | 92812 | Radix Angelicae Sinensis |
| MOL001390 | Globulol | C15H26O | 222.41 | 489-41-8 | 12304985 | Rhizoma Chuanxiong |
| MOL001393 | myristic acid | C14H28O2 | 228.42 | 45184-05-2 | 11005 | Radix Bupleuri, Flos Carthami, Radix Achyranthis Bidentatae |
| MOL001396 | Pentadecylic acid | C15H30O2 | 242.45 | 1002-84-2 | 13849 | Radix Bupleuri, Flos Carthami, Radix Rehmanniae |
| MOL001398 | Methyllinolenate | C19H32O2 | 292.51 | 301-00-8 | 5319706 | Flos Carthami |
| MOL001402 | Octacosane | C28H58 | 394.86 | 630-02-4 | 12408 | Flos Carthami |
| MOL001417 | Trans-2-Octenal | C8H14O | 126.22 | 25447-69-2 | 5283324 | Radix Bupleuri |
| MOL001436 | leonuride | C15H24O9 | 348.39 | 50906-66-6 | 46227138 | Radix Rehmanniae |
| MOL001442 | phytol | C20H40O | 296.6 | 5016-81-9 | 5280435 | Flos Carthami |
| MOL001454 | berberine | C20H18NO4+ | 336.39 | 2086-83-1 | 2353 | Radix Achyranthis Bidentatae |
| MOL001458 | coptisine | Not Available | 320.34 | 3486-66-6 | Not Available | Radix Achyranthis Bidentatae |
| MOL001484 | Inermine | C16H12O5 | 284.28 | 2035-15-6 | 91510 | Radix et Rhizoma Glycyrrhizae |
| MOL001487 | FITONE | C18H36O | 268.54 | 502-69-2 | 1810796 | Radix Bupleuri |
| MOL001494 | Mandenol | C20H36O2 | 308.56 | 544-35-4 | 5282184 | Rhizoma Chuanxiong |
| MOL001501 | Daturic acid | C17H34O2 | 270.51 | 67701-03-5 | 10465 | Flos Carthami, Radix Rehmanniae |
| MOL001543 | Vicenin-2 | C27H30O15 | 594.57 | 23666-13-9 | 442664 | Radix et Rhizoma Glycyrrhizae |
| MOL001551 | Trochol | C30H50O2 | 442.8 | 18211-63-7 | 72326 | Radix Platycodonis |
| MOL001578 | Hypnon | C8H8O | 120.16 | 98-86-2 | 7410 | Radix Angelicae Sinensis |
| MOL001579 | germacrene D | C15H24 | 208.43 | 23986-74-5 | Not Available | Rhizoma Chuanxiong |
| MOL001599 | α-cubebol | Not Available | 208.38 | 81-34-5 | Not Available | Radix et Rhizoma Glycyrrhizae |
| MOL001600 | copaene | C15H24 | 204.39 | 3856-25-5 | 92042749 | Radix Bupleuri |
| MOL001640 | Decanoic acid | C10H20O2 | 172.3 | 334-48-5 | 2969 | Radix Angelicae Sinensis , Flos Carthami |
| MOL001641 | Methyl linoleate | C19H34O2 | 294.53 | 112-63-0 | 5284421 | Rhizoma Chuanxiong |
| MOL001645 | Linoleyl acetate | C20H36O2 | 308.56 | 5999-95-1 | 5319042 | Radix Bupleuri |
| MOL001655 | oleanolic acid-3-O-β-D-glucuronopyranoside_qt | C30H48O3 | 456.78 | Not Available | 25728252 | Radix Achyranthis Bidentatae |
| MOL001689 | acacetin | C16H12O5 | 284.28 | 480-44-4 | 5280442 | Radix Platycodonis |
| MOL001691 | vitamin c | C6H8O6 | 176.14 | 50-81-7 | 54670067 | Radix Platycodonis |
| MOL001695 | Quercimeritrin (6CI,7CI,8CI) | C21H20O12 | 464.41 | 491-50-9 | 5282160 | Flos Carthami |
| MOL001696 | Morusin | C25H24O6 | 420.49 | 62596-29-6 | 5281671 | Radix et Rhizoma Glycyrrhizae |
| MOL001729 | Crysophanol | C15H10O4 | 254.25 | 481-74-3 | 10208 | Rhizoma Chuanxiong, Radix Achyranthis Bidentatae |
| MOL001731 | Dextrose | C6H12O6 | 180.18 | 492-62-6 | 79025 | Radix Bupleuri |
| MOL001737 | Indole-3-carboxylic acid | C9H7NO2 | 161.17 | 771-50-6 | 69867 | Radix et Rhizoma Glycyrrhizae |
| MOL001739 | Palmitoleic acid | C16H30O2 | 254.46 | 373-49-9 | 445638 | Flos Carthami, Radix Rehmanniae |
| MOL001744 | uracil | C4H4N2O2 | 112.1 | 66-22-8 | 1174 | Rhizoma Chuanxiong |
| MOL001747 | Tetracosane | C24H50 | 338.74 | 646-31-1 | 12592 | Radix Achyranthis Bidentatae |
| MOL001752 | 4-vinylguaiacol | C9H10O2 | 150.19 | 7786-61-0 | 332 | Rhizoma Chuanxiong |
| MOL001768 | Sumiki's acid | C6H6O4 | 142.12 | 6338-41-6 | 80642 | Radix Rehmanniae |
| MOL001771 | Clionasterol | C29H50O | 414.79 | 201-481-1 | 457801 | Flos Carthami |
| MOL001787 | Adenosine | C10H13N5O4 | 267.28 | 30143-02-3 | 60961 | Rhizoma Chuanxiong, Flos Carthami |
| MOL001788 | adenine | C5H5N5 | 135.15 | 73-24-5 | 190 | Rhizoma Chuanxiong, Radix Angelicae Sinensis, Flos Carthami |
| MOL001789 | isoliquiritigenin | C15H12O4 | 256.27 | 961-29-5 | 638278 | Radix Bupleuri, Radix et Rhizoma Glycyrrhizae |
| MOL001792 | liquiritigenin | C15H12O4 | 256.27 | 578-86-9 | 114829 | Radix et Rhizoma Glycyrrhizae |
| MOL001801 | salicylic acid | C7H6O3 | 138.13 | 7681/6/3 | 338 | Radix Paeoniae Rubra, Flos Carthami, |
| MOL001836 | n-butyl-β-D-fructopyronoside | C10H20O6 | 236.3 | Not Available | 50914217 | Radix Achyranthis Bidentatae |
| MOL001850 | Izoforon | C9H14O | 138.23 | 78-59-1 | 6544 | Radix et Rhizoma Glycyrrhizae |
| MOL001901 | 24-Methylenecycloartanol | C31H52O | 440.83 | 1449-09-8 | 94204 | Semen Persicae |
| MOL001906 | Methylgallate | C8H8O5 | 184.16 | 99-24-1 | 7428 | Radix Paeoniae Rubra |
| MOL001907 | Progallin A | C9H10O5 | 198.19 | 52441-13-1 | 13250 | Radix Paeoniae Rubra |
| MOL001918 | Paeoniflorgenone | C17H18O6 | 318.35 | 84877-44-1 | Not Available | Radix Paeoniae Rubra |
| MOL001924 | paeoniflorin | C23H28O11 | 480.51 | 23180-57-6 | Not Available | Radix Paeoniae Rubra |
| MOL001955 | Chlorogenate | C16H18O9 | 354.34 | 202650-88-2 | 1794427 | Flos Carthami, Semen Persicae |
| MOL001972 | Pulegone | C10H16O | 152.26 | 89-82-7 | 442495 | Radix Bupleuri |
| MOL001999 | scoparone | C11H10O4 | 206.21 | 120-08-1 | 8417 | Radix Bupleuri |
| MOL002002 | cis-Carveol | C10H16O | 152.26 | 1197-06-4 | 330573 | Radix Bupleuri |
| MOL002003 | (-)-Caryophyllene oxide | C15H24O | 220.39 | 1139-30-6 | Not Available | Flos Carthami |
| MOL002008 | myricetin | C15H10O8 | 318.25 | 529-44-2 | 5281672 | Flos Carthami |
| MOL002028 | (+)-beta-Phellandrene | C10H16 | 136.26 | 555-10-2 | 442484 | Rhizoma Chuanxiong |
| MOL002029 | Cuparene | C15H22 | 202.37 | 16982-00-6 | 86895 | Radix Bupleuri, Radix Angelicae Sinensis |
| MOL002033 | cis-Thujopsene | C15H24 | 204.39 | 470-40-6 | 442402 | Radix Angelicae Sinensis |
| MOL002042 | thymol | C10H14O | 150.24 | 89-83-8 | 6989 | Radix Bupleuri, Rhizoma Chuanxiong |
| MOL002046 | hexanoic acid | C6H12O2 | 116.18 | 142-62-1 | 8892 | Radix Bupleuri, Flos Carthami, Radix Achyranthis Bidentatae |
| MOL002085 | alpha-Cubebene | C15H24 | 204.39 | 17699-14-8 | 42608159 | Radix Bupleuri, Rhizoma Chuanxiong |
| MOL002095 | Diethyl phthalate | C12H14O4 | 222.26 | 68988-18-1 | 6781 | Radix Bupleuri |
| MOL002096 | (+)-ALPHA-FUNEBRENE | C15H24 | 204.39 | 50894-66-1 | Not Available | Rhizoma Chuanxiong |
| MOL002097 | gem-Dimethylcyclopentane | C7H14 | 98.21 | 1638-26-2 | 15421 | Rhizoma Chuanxiong |
| MOL002098 | 3-Butylidene-7-hydroxyphthalide | C12H12O2 | 204.24 | 93236-67-0 | 5281559 | Rhizoma Chuanxiong, Radix Angelicae Sinensis |
| MOL002099 | Senkyunolide-K | C12H12O3 | 208.28 | 114569-33-4 | 13965751 | Rhizoma Chuanxiong |
| MOL002102 | Levistolid A | C24H28O4 | 380.52 | 88182-33-6 | 70698035 | Rhizoma Chuanxiong, Radix Angelicae Sinensis |
| MOL002103 | Senkyunolide-P | C24H30O4 | 382.54 | 142864-23-1 | 91731751 | Rhizoma Chuanxiong |
| MOL002107 | Valerophenone | C11H14O | 162.25 | 1009-14-9 | 66093 | Radix Bupleuri, Rhizoma Chuanxiong |
| MOL002108 | (1S,5S)-7,7-dimethyl-2-methylenebicyclo[3.1.1] hept-3-ene | C10H14 | 134.24 | 4080-46-0 | 21631119 | Rhizoma Chuanxiong |
| MOL002109 | Z-6,8',7,3'-diligustilide | Not Available | 380.52 | Not Available | Not Available | Rhizoma Chuanxiong |
| MOL002111 | N-butylidenephthalide | C12H12O2 | 188.24 | 76681-73-7 | 5352899 | Radix Bupleuri, Rhizoma Chuanxiong, Radix Angelicae Sinensis |
| MOL002112 | alpha-Selinene | C16H26 | 218.42 | 473-13-2 | Not Available | Rhizoma Chuanxiong |
| MOL002114 | augustic-acid | C30H48O4 | 472.78 | 26707-60-8 | 15560128 | Rhizoma Chuanxiong |
| MOL002115 | m-Ethyltoluene | C9H12 | 120.21 | 620-14-4 | 12100 | Rhizoma Chuanxiong |
| MOL002116 | (1R,4S,5R)-4-isopropenyl-1,8-dimethylspiro[4.5]dec-8-ene | C15H24 | 204.39 | 28477-64-7 | 20055537 | Radix Bupleuri, Rhizoma Chuanxiong |
| MOL002117 | β-sesquiphellandrene | C15H24 | 204.39 | 20307-83-9 | 12315492 | Rhizoma Chuanxiong |
| MOL002119 | Artemisia triene | C10H16 | 136.26 | 29548-02-5 | 5320377 | Rhizoma Chuanxiong |
| MOL002120 | betea-CUBEBENE | C15H24 | 204.39 | 13744-15-5 | 6432083 | Rhizoma Chuanxiong |
| MOL002121 | (1S,4E,8E,10R)-4,8,11,11-tetramethylbicyclo [8.1.0]undeca-4,8-diene | C15H24 | 204.39 | 24703-35-3 | 11820258 | Rhizoma Chuanxiong |
| MOL002122 | (Z)-Ligustilide | C12H12O2 | 188.24 | 551-08-6 | 642376 | Rhizoma Chuanxiong |
| MOL002123 | Chuanxiongol | C13H14O3 | 218.27 | 87421-30-5 | 5315862 | Rhizoma Chuanxiong |
| MOL002124 | beta-asarone | C12H16O3 | 208.28 | 5273-86-9 | 5281758 | Rhizoma Chuanxiong |
| MOL002125 | cis-Piperitol | C10H18O | 154.28 | 16721-38-3 | 85567 | Rhizoma Chuanxiong |
| MOL002126 | (1S,4R,5R)-1-isopropyl-4-methyl-4-bicyclo [3.1.0] hexanol | C10H18O | 154.28 | 546-79-2 | 6326181 | Rhizoma Chuanxiong |
| MOL002127 | Cnidilide | C12H18O2 | 194.3 | 3674/3/1 | 160710 | Rhizoma Chuanxiong |
| MOL002128 | 1,3,8-p-Menthatriene | C10H14 | 134.24 | 18368-95-1 | 176983 | Rhizoma Chuanxiong |
| MOL002129 | (Z)-Cyclododecene | C12H22 | 166.34 | 1501-82-2 | 637538 | Rhizoma Chuanxiong |
| MOL002130 | cyclohexane,1,1,2,3-tetramethyl- | C10H20 | 140.3 | 6783-92-2 | 57532263 | Rhizoma Chuanxiong |
| MOL002133 | 1,5,5-trimethyl-6-methylenecyclohexene | C10H16 | 136.26 | 514-95-4 | 578237 | Rhizoma Chuanxiong |
| MOL002134 | Isobutyrophenone | C10H12O | 148.22 | 611-70-1 | 69144 | Rhizoma Chuanxiong |
| MOL002135 | Myricanone | C21H24O5 | 356.45 | 32492-74-3 | 161748 | Rhizoma Chuanxiong |
| MOL002136 | Isocnidilide | C12H18O2 | 194.3 | 4567-33-3 | 12315453 | Rhizoma Chuanxiong |
| MOL002138 | p-Cymen-8-ol | C10H14O | 150.24 | 1197-01-9 | 14529 | Radix Bupleuri, Rhizoma Chuanxiong |
| MOL002140 | Perlolyrine | C16H12N2O2 | 264.3 | 29700-20-7 | 160179 | Rhizoma Chuanxiong |
| MOL002141 | Pregnenolone | C21H32O2 | 316.53 | 145-13-1 | 8955 | Radix Bupleuri, Rhizoma Chuanxiong |
| MOL002142 | sedanoic acid | C12H18O3 | 210.3 | 6697-07-0 | 12367058 | Rhizoma Chuanxiong |
| MOL002143 | senkyunolide C | C12H12O3 | 204.24 | 91652-78-7 | 642374 | Rhizoma Chuanxiong, Radix Angelicae Sinensis |
| MOL002144 | senkyunolide D | C12H14O4 | 222.26 | 94530-82-2 | 11264524 | Rhizoma Chuanxiong, Radix Angelicae Sinensis |
| MOL002145 | senkyunolide E | C12H12O3 | 204.24 | 94530-83-3 | 11830530 | Rhizoma Chuanxiong, Radix Angelicae Sinensis |
| MOL002146 | senkyunolide F | C12H14O3 | 206.26 | 94530-84-4 | 11241196 | Rhizoma Chuanxiong |
| MOL002150 | 1-Acetyl-beta-carboline | C13H10N2O | 210.25 | 50892-83-6 | 638667 | Rhizoma Chuanxiong |
| MOL002152 | sinapic acid | C11H12O5 | 224.23 | 530-59-6 | 637775 | Rhizoma Chuanxiong |
| MOL002153 | Spathulenol | C15H24O | 220.39 | 6750-60-3 | 92231 | Rhizoma Chuanxiong |
| MOL002154 | trans-2-Nonen-1-ol | C9H18O | 142.27 | 22104-79-6 | 5364941 | Rhizoma Chuanxiong |
| MOL002156 | Trimethylamine | C3H9N | 59.13 | 75-50-3 | 1146 | Rhizoma Chuanxiong |
| MOL002157 | wallichilide | C25H32O5 | 412.57 | 93236-64-7 | 10873344 | Rhizoma Chuanxiong |
| MOL002160 | 1-terpineol | C10H18O | 154.28 | 586-82-3 | 12443330 | Rhizoma Chuanxiong |
| MOL002161 | 1-beta-ethylacrylate-7-aldehyde-beta-carboline | Not Available | 294.33 | Not Available | Not Available | Rhizoma Chuanxiong |
| MOL002162 | 2-Propionylfuran | C7H8O2 | 124.15 | 3194-15-8 | 76662 | Rhizoma Chuanxiong |
| MOL002163 | Propiophenone | C9H10O | 134.19 | 93-55-0 | 7148 | Rhizoma Chuanxiong |
| MOL002165 | methyl 2-pentanoylbenzoate | C13H16O3 | 220.29 | Not Available | 11085323 | Rhizoma Chuanxiong |
| MOL002166 | Isoheptanes | C7H16 | 100.23 | 591-76-4 | 11582 | Rhizoma Chuanxiong, Radix et Rhizoma Glycyrrhizae |
| MOL002167 | WLN: T5OJ BVO1 | C6H6O3 | 126.12 | 611-13-2 | 11902 | Rhizoma Chuanxiong |
| MOL002168 | 2-Methyl-1-phenylpropene | C10H12 | 132.22 | 768-49-0 | 13030 | Rhizoma Chuanxiong |
| MOL002169 | 2-methyl-5-(1-methylene)-1,3-cyclohexadiene | Not Available | 106.18 | Not Available | Not Available | Rhizoma Chuanxiong |
| MOL002170 | (4S,6S)-cis-Carveol | C10H16O | 152.26 | Not Available | 443177 | Rhizoma Chuanxiong |
| MOL002172 | (5S,6R)-5,6-dimethyltetrahydropyran-2-one | C7H12O2 | 128.19 | 24405-16-1 | 11029861 | Rhizoma Chuanxiong |
| MOL002174 | 3,4-epoxy-2,2,7,7-tetramethyl-octane | Not Available | 184.36 | Not Available | Not Available | Rhizoma Chuanxiong |
| MOL002176 | Methyl 3-furoate | C6H6O3 | 126.12 | 1334-76-5 | 14918 | Rhizoma Chuanxiong |
| MOL002177 | trans-Piperitol | C10H18O | 154.28 | 16721-39-4 | 85568 | Rhizoma Chuanxiong |
| MOL002178 | 4,7-Dihydroxy-3-butylphthalide | Not Available | 222.26 | Not Available | Not Available | Rhizoma Chuanxiong |
| MOL002181 | 4-hydroxy-3-butylphthalide | Not Available | 206.26 | Not Available | Not Available | Rhizoma Chuanxiong |
| MOL002184 | (6R)-6-butylcyclohepta-1,4-diene | C11H18 | 150.29 | 33156-91-1 | 11804943 | Rhizoma Chuanxiong, Radix Angelicae Sinensis |
| MOL002185 | 7-oxabicyclo-2.2.1-heptane,1-methyl-4-[1-methylethyl]- | C10H18O | 154.28 | 470-67-7 | 10106 | Rhizoma Chuanxiong |
| MOL002186 | Aromadendrene oxide 2 | C15H24O | 220.39 | 85710-39-0 | 16211192 | Rhizoma Chuanxiong |
| MOL002187 | Amylbenzene | C11H16 | 148.27 | 27458-20-4 | 10864 | Radix Bupleuri, Rhizoma Chuanxiong |
| MOL002188 | Dimethyl D-malate | C6H10O5 | 162.16 | 70681-41-3 | 11062697 | Rhizoma Chuanxiong |
| MOL002189 | dl-3n-butylphthalide | C12H14O2 | 190.26 | 125412-70-6 | 9990075 | Rhizoma Chuanxiong |
| MOL002190 | Cedrene | C15H24 | 204.39 | 469-61-4 | Not Available | Rhizoma Chuanxiong |
| MOL002191 | carotol | C15H26O | 222.41 | 465-28-1 | 442347 | Rhizoma Chuanxiong |
| MOL002192 | Coniferyl fcrulate | C20H20O6 | 356.4 | 63644-62-2 | 6441913 | Rhizoma Chuanxiong |
| MOL002193 | Cerulignol | C10H14O2 | 166.24 | 2785-87-7 | 17739 | Rhizoma Chuanxiong |
| MOL002198 | Heptan | C7H16 | 100.23 | 142-82-5 | 8900 | Rhizoma Chuanxiong, Radix et Rhizoma Glycyrrhizae |
| MOL002199 | L-valyl-L-valinc-achydride | Not Available | 214.35 | 350702-73-7 | Not Available | Rhizoma Chuanxiong |
| MOL002200 | Levistolide-A | C24H28O4 | 380.52 | 88182-33-6 | 70698035 | Rhizoma Chuanxiong |
| MOL002201 | cis-ligustilide | C12H14O2 | 190.26 | 4431-01-0 | 5877292 | Radix Bupleuri, Rhizoma Chuanxiong, Radix Angelicae Sinensis |
| MOL002202 | tetramethylpyrazine | C8H12N2 | 136.22 | 87396-75-6 | 14296 | Rhizoma Chuanxiong |
| MOL002203 | Exceparl M-OL | C19H36O2 | 296.55 | 139152-82-2 | 5364509 | Rhizoma Chuanxiong |
| MOL002206 | (2-amylphenyl)methanol | C12H18O | 178.3 | Not Available | 10058085 | Rhizoma Chuanxiong |
| MOL002207 | Neocnidilide | C12H18O2 | 194.3 | 4567-33-3 | 3083857 | Rhizoma Chuanxiong |
| MOL002208 | Senkyunolide A | C12H16O2 | 192.28 | 63038-10-8 | 3085257 | Rhizoma Chuanxiong |
| MOL002209 | Senkyunolide G | C12H16O3 | 208.28 | 94530-85-5 | 5321250 | Rhizoma Chuanxiong |
| MOL002311 | Glycyrol | C21H18O6 | 366.39 | 23013-84-5 | 5320083 | Radix et Rhizoma Glycyrrhizae |
| MOL002321 | Pidolic acid | C5H7NO3 | 129.13 | 16891-48-8 | 7405 | Radix Rehmanniae |
| MOL002335 | beta-Gurjunene | C15H24 | 204.39 | 73464-47-8 | 6450812 | Radix Bupleuri |
| MOL002341 | Hesperetin | C16H14O6 | 302.3 | 520-33-2 | 72281 | Fructus Aurantii |
| MOL002347 | (R)-Allantoin | C4H6N4O3 | 158.14 | 97-59-6 | 439713 | Radix Achyranthis Bidentatae |
| MOL002365 | (s)-carvone | C10H14O | 150.24 | 53763-73-8 | 16724 | Radix Bupleuri |
| MOL002375 | (-)-isomenthone | C10H18O | 154.28 | 491-07-6 | 6432469 | Radix Bupleuri |
| MOL002379 | Valeraldehyde | C5H10O | 86.15 | 110-62-3 | 8063 | Radix Bupleuri, Radix Achyranthis Bidentatae, |
| MOL002480 | Methylbutenol | C5H10O | 86.15 | 115-18-4 | 8257 | Radix Angelicae Sinensis |
| MOL002547 | 21987_FLUKA | C10H16 | 136.26 | 554-61-0 | 16211586 | Radix et Rhizoma Glycyrrhizae |
| MOL002565 | Medicarpin | C16H14O4 | 270.3 | 32383-76-9 | 336327 | Radix et Rhizoma Glycyrrhizae |
| MOL002579 | capsaicin | C18H27NO3 | 305.46 | 404-86-4 | 1548943 | Radix Bupleuri |
| MOL002643 | delta 7-stigmastenol | C29H50O | 414.79 | 6869-99-4 | 3080632 | Radix Achyranthis Bidentatae |
| MOL002676 | Aplotaxene | C17H28 | 232.45 | 10482-53-8 | 5352710 | Flos Carthami |
| MOL002678 | EB | C8H10 | 106.18 | 70955-17-8 | 7500 | Radix et Rhizoma Glycyrrhizae, Flos Carthami |
| MOL002679 | Ethone | C7H16O3 | 148.23 | 108055-42-1 | 31214 | Flos Carthami |
| MOL002683 | Ligla | C18H30O2 | 278.48 | 506-26-3 | 5280933 | Flos Carthami |
| MOL002684 | gamma-Tocotrienol | C28H42O2 | 410.7 | 14101-61-2 | 5282349 | Flos Carthami |
| MOL002686 | Glyoxylic acid | C2H2O3 | 74.04 | 298-12-4 | 760 | Flos Carthami |
| MOL002689 | 3,4,5-Trimethoxytoluene | C10H14O3 | 182.24 | 6443-69-2 | 80922 | Flos Carthami |
| MOL002691 | ISOVALERIC ACID | C5H10O2 | 102.15 | 92634-50-9 | 10430 | Flos Carthami |
| MOL002692 | Amoenin A3 | C21H20O11 | 448.41 | Not Available | 5742687 | Flos Carthami |
| MOL002693 | nicotiflorin | C27H30O15 | 594.57 | 17650-84-9 | 5318767 | Radix et Rhizoma Glycyrrhizae, Flos Carthami |
| MOL002694 | Kinobeon A | C20H20O6 | 356.4 | 155239-87-5 | 10237057 | Flos Carthami |
| MOL002695 | lignan | C25H30O8 | 458.55 | 6549-68-4 | 261166 | Flos Carthami |
| MOL002696 | lirioresinol-A | C22H26O8 | 404.45 | 21453-71-4 | 12309694 | Flos Carthami |
| MOL002697 | junipene | C15H24 | 204.39 | 475-20-7 | 10137 | Flos Carthami |
| MOL002699 | Cetene | C16H32 | 224.48 | 26952-14-7 | 12395 | Flos Carthami |
| MOL002701 | neocarthamin | C21H22O11 | 450.43 | Not Available | 46173973 | Flos Carthami |
| MOL002704 | D-Phenylalanine | C9H11NO2 | 165.21 | 673-06-3 | 6919011 | Flos Carthami |
| MOL002708 | precarthamin | Not Available | 464.51 | Not Available | Not Available | Flos Carthami |
| MOL002710 | Pyrethrin II | C22H28O5 | 372.5 | 121-29-9 | 5281555 | Flos Carthami |
| MOL002711 | 1-Penten-3-ol | C5H10O | 86.15 | 93222-01-6 | 6994331 | Flos Carthami |
| MOL002712 | 6-Hydroxykaempferol | C15H10O7 | 302.25 | 4324-55-4 | 5281638 | Flos Carthami |
| MOL002714 | baicalein | C15H10O5 | 270.25 | 491-67-8 | 5281605 | Radix Paeoniae Rubra, Flos Carthami, Radix Achyranthis Bidentatae |
| MOL002717 | qt_carthamone | Not Available | 286.25 | Not Available | Not Available | Flos Carthami |
| MOL002721 | quercetagetin | C15H10O8 | 318.25 | 90-18-6 | 5281680 | Flos Carthami |
| MOL002722 | Vomifoliol | C13H20O3 | 224.33 | 23526-45-6 | 5280462 | Flos Carthami |
| MOL002724 | qt_safflomin-C | Not Available | 452.44 | Not Available | Not Available | Flos Carthami |
| MOL002725 | qt_safflow-yellow-A | Not Available | 448.41 | Not Available | Not Available | Flos Carthami |
| MOL002728 | sesquiterpene | C30H34O13 | 626.57 | Not Available | 6473767 | Flos Carthami |
| MOL002730 | Vitamin- G | C17H20N4O6 | 376.41 | 83-88-5 | 493570 | Flos Carthami |
| MOL002731 | (4S)-4-hydroxy-3,5,5-trimethyl-4-[(E,3R)-3-[(2R, 3R,4S,5S,6R)-3,4,5-trihydroxy-6-(hydroxymethyl) tetrahydropyran-2-yl]oxybut-1-enyl]cyclohex-2-en-1-one | C19H30O8 | 386.49 | 54835-70-0 | 9930064 | Flos Carthami |
| MOL002737 | scutellarein | C15H10O6 | 286.25 | 529-53-3 | 5281697 | Flos Carthami |
| MOL002739 | tagetiin | C21H20O13 | 480.41 | 60671-81-0 | 11968966 | Flos Carthami |
| MOL002740 | thymopentin | C30H49N9O9 | 679.88 | 69558-55-0 | 451417 | Flos Carthami |
| MOL002743 | (R)-(-)-2-Hexanol | C6H14O | 102.2 | 26549-24-6 | 6993810 | Flos Carthami |
| MOL002744 | (R)-2-methylbutyric acid | C5H10O2 | 102.15 | 116-53-0 | 6950479 | Flos Carthami |
| MOL002745 | (r)-3-hexanol | C6H14O | 102.2 | 623-37-0 | 6994293 | Flos Carthami |
| MOL002746 | Cincofarm | C11H12N2O3 | 220.25 | 4350/9/8 | 6971044 | Flos Carthami |
| MOL002747 | (2R)-5-methylhexan-2-ol | C7H16O | 116.23 | 627-59-8 | 6999721 | Flos Carthami |
| MOL002751 | 6-hydroxykaempferol-3-O-beta-D-glucoside | Not Available | 464.41 | Not Available | Not Available | Flos Carthami |
| MOL002753 | 6-hydroxykaempferol-3-O-glucoside | C21H20O12 | 464.41 | Not Available | 25017112 | Flos Carthami |
| MOL002754 | 6-hydroxykaempferol-3,6-di-O-beta-D-glucoside | Not Available | 626.57 | Not Available | Not Available | Flos Carthami |
| MOL002757 | 7,8-dimethyl-1H-pyrimido[5,6-g] quinoxaline-2,4-dione | C12H10N4O2 | 242.26 | Not Available | 21786815 | Flos Carthami |
| MOL002761 | Sophoraflavonoloside | C27H30O16 | 610.57 | 19895-95-5 | 5282155 | Flos Carthami |
| MOL002764 | Tricoumaroyl spermidine | C34H37N3O6 | 583.74 | Not Available | 14777879 | Flos Carthami |
| MOL002765 | N1,N5-(Z)-N10-(E)-tri-p-coumaroylspermidine | C34H37N3O6 | 583.74 | Not Available | 50942902 | Flos Carthami |
| MOL002766 | Quercetin-3,7-di-O-beta-d-glucoside | Not Available | 626.57 | Not Available | Not Available | Flos Carthami |
| MOL002771 | Vitamin E | C29H50O2 | 430.79 | 1406-18-4 | 14985 | Flos Carthami |
| MOL002773 | beta-carotene | C40H56 | 536.96 | 7235-40-7 | 5280489 | Flos Carthami |
| MOL002775 | Beta-tocopherol | C28H48O2 | 416.76 | 16698-35-4 | 6857447 | Flos Carthami |
| MOL002776 | Baicalin | C21H18O11 | 446.39 | 31564-28-0 | 64982 | Radix Bupleuri, Radix Paeoniae Rubra, Flos Carthami, Radix Achyranthis Bidentatae |
| MOL002781 | carthamone | C21H20O11 | 448.41 | 86579-00-2 | 5281241 | Flos Carthami |
| MOL002783 | Cumalic acid | C6H4O4 | 140.1 | 500-05-0 | 68141 | Flos Carthami |
| MOL002786 | Apocynin | C9H10O3 | 166.19 | 498-02-2 | 2214 | Radix Bupleuri |
| MOL002819 | catalpol | C15H22O10 | 362.37 | 2415-24-9 | 91520 | Radix Rehmanniae |
| MOL002820 | catapol_qt | C9H10O5 | 200.21 | 6736-85-2 | Not Available | Radix Rehmanniae |
| MOL002830 | 4-Methylphenol | C5H10O | 108.15 | 72269-62-6 | 2879 | Radix Bupleuri, Radix Angelicae Sinensis |
| MOL002844 | Pinocembrin | C15H12O4 | 256.27 | 480-39-7 | 68071 | Radix et Rhizoma Glycyrrhizae |
| MOL002850 | butylated hydroxytoluene | C15H24O | 220.39 | 50356-19-9 | 31404 | Radix et Rhizoma Glycyrrhizae |
| MOL002851 | ZINC05224268 | C15H24O | 220.39 | 118-65-0 | Not Available | Fructus Aurantii |
| MOL002885 | Valeric acid | C5H10O2 | 102.15 | 109-52-4 | 7991 | Radix Bupleuri |
| MOL002897 | epiberberine | C20H18NO4+ | 336.39 | 6873/9/2 | 160876 | Radix Achyranthis Bidentatae |
| MOL002936 | 5,8-Dihydroxy-6,7-dimethoxyflavone | C17H14O6 | 314.31 | 73202-52-5 | 153441 | Radix Bupleuri |
| MOL002972 | (4S)-1-methyl-4-(6-methylhepta-1,5-dien-2-yl)cyclohexene | C15H24 | 204.39 | 495-61-4 | 10104370 | Radix Bupleuri, Radix Angelicae Sinensis |
| MOL002983 | Guasol | C7H8O2 | 124.15 | 8021-39-4 | 460 | Radix Bupleuri, Radix Angelicae Sinensis |
| MOL003040 | Amylol | C5H12O | 88.17 | 71-41-0 | 6276 | Radix Bupleuri, Radix Achyranthis Bidentatae, |
| MOL003050 | nonanoic acid | C9H18O2 | 158.27 | 112-05-0 | 8158 | Radix Bupleuri |
| MOL003127 | Tripteroside | C19H18O11 | 204.39 | 82855-00-3 | 5281664 | Rhizoma Chuanxiong |
| MOL003202 | 8-Epiloganic acid | C16H24O10 | 376.4 | 82509-41-9 | 158144 | Radix Rehmanniae |
| MOL003218 | Neouralenol | C20H18O7 | 370.38 | Not Available | 5320118 | Radix et Rhizoma Glycyrrhizae |
| MOL003367 | Myricadiol | C30H50O2 | 442.8 | 17884-88-7 | 3084282 | Radix Bupleuri |
| MOL003493 | naphthalene | C10H8 | 128.18 | 72931-45-4 | 931 | Radix Bupleuri |
| MOL003509 | Nonanol | C9H20O | 144.29 | 143-08-8 | 8914 | Radix Bupleuri |
| MOL003534 | CADINENE | C15H24 | 204.39 | 523-47-7 | 10657 | Radix Angelicae Sinensis |
| MOL003573 | calacorene | C15H20 | 200.35 | 21391-99-1 | 12302243 | Radix Bupleuri |
| MOL003587 | Acoradiene | C15H24 | 204.39 | 24048-44-0 | 90351 | Radix Bupleuri, Radix Angelicae Sinensis |
| MOL003590 | angelicin | C11H6O3 | 186.17 | 39310-13-9 | 10658 | Radix Bupleuri |
| MOL003656 | Lupiwighteone | C20H18O5 | 338.38 | 104691-86-3 | 5317480 | Radix et Rhizoma Glycyrrhizae |
| MOL003686 | Narcissoside | C28H32O16 | 624.6 | 604-80-8 | 5481663 | Radix Bupleuri, Radix et Rhizoma Glycyrrhizae |
| MOL003689 | aeginetic acid | C15H24O4 | 268.39 | 53537-92-1 | 15693867 | Radix Rehmanniae |
| MOL003690 | Ajugol | C15H24O9 | 348.39 | 52949-83-4 | 6325127 | Radix Rehmanniae |
| MOL003692 | Ajugoside_qt | Not Available | 212.27 | 52916-96-8 | Not Available | Radix Rehmanniae |
| MOL003696 | Cistanoside F | C21H28O13 | 488.49 | Not Available | 44429870 | Radix Rehmanniae |
| MOL003698 | (2S,3R,4R,5S,6R)-2-[[(1S,4aS,5R,7aR)-4a,5-dihydroxy-7-methylol-5,7a-dihydro-1H-cyclopenta[c]pyran-1-yl]oxy]-6-methylol-tetrahydropyran-3,4,5-triol | C15H22O10 | 362.37 | Not Available | 21550273 | Radix Rehmanniae |
| MOL003699 | melittoside_qt | Not Available | 200.21 | Not Available | Not Available | Radix Rehmanniae |
| MOL003700 | Dihydrocatalpol | C15H24O10 | 364.39 | Not Available | 5705531 | Radix Rehmanniae |
| MOL003702 | geniposide_qt | C17H24O10 | 226.25 | 24512-63-8 | 107848 | Radix Achyranthis Bidentatae, Radix Rehmanniae |
| MOL003705 | jioglutin A | Not Available | 250.7 | Not Available | Not Available | Radix Rehmanniae |
| MOL003706 | jioglutin B | Not Available | 250.7 | Not Available | Not Available | Radix Rehmanniae |
| MOL003708 | jioglutin D | C11H18O6 | 246.29 | Not Available | 14486934 | Radix Rehmanniae |
| MOL003709 | jioglutin E | C11H20O5 | 232.31 | Not Available | 14486937 | Radix Rehmanniae |
| MOL003710 | jioglutolide | C9H14O4 | 186.23 | 124902-18-7 | 101260546 | Radix Rehmanniae |
| MOL003711 | Jioglutoside A | Not Available | 346.37 | Not Available | Not Available | Radix Rehmanniae |
| MOL003717 | Methyl palmitoleate | C17H32O2 | 268.49 | 1120-25-8 | 643801 | Radix Rehmanniae |
| MOL003718 | 6-O-p-coumaroylajugol | Not Available | 524.57 | Not Available | Not Available | Radix Rehmanniae |
| MOL003722 | Rehmaglutin C | C9H12O5 | 200.21 | 103744-81-6 | 21637649 | Radix Rehmanniae |
| MOL003723 | Rehmaionoside A | C19H34O8 | 390.53 | 104112-06-3 | 10023290 | Radix Rehmanniae |
| MOL003724 | Rehmaionoside B | C19H34O8 | 390.53 | 104056-83-9 | 10430488 | Radix Rehmanniae |
| MOL003725 | Rehmaionoside C | C19H32O8 | 388.51 | 104112-05-2 | 11740990 | Radix Rehmanniae |
| MOL003732 | Rehmaglutin D | Not Available | 220.67 | 103744-84-9 | 5320906 | Radix Rehmanniae |
| MOL003733 | 6-O-vanilloylajugol | C23H30O12 | 484.5 | 124168-04-3 | 14396664 | Radix Rehmanniae |
| MOL003735 | aucubin | C15H22O9 | 346.37 | 479-98-1 | 91458 | Radix Rehmanniae |
| MOL003778 | pjs-1_qt | Not Available | 456.78 | 14162-53-9 | Not Available | Radix Achyranthis Bidentatae |
| MOL003786 | patchoulane | C15H26 | 206.41 | 19078-35-4 | 29408 | Radix Bupleuri |
| MOL003837 | esculetin | C9H6O4 | 178.15 | 305-01-1 | 5281416 | Radix Bupleuri |
| MOL003847 | Inophyllum E | C25H22O5 | 402.47 | 17312-31-1 | 5254 | Radix Achyranthis Bidentatae |
| MOL003867 | Paeonolide | C20H28O12 | 460.48 | 72520-92-4 | 442923 | Radix Paeoniae Rubra |
| MOL003896 | 7-Methoxy-2-methyl isoflavone | C17H14O3 | 266.31 | 19725-44-1 | 354368 | Radix et Rhizoma Glycyrrhizae |
| MOL004067 | (+)-Nootkatone | C15H22O | 218.37 | 4674-50-4 | 1268142 | Radix Bupleuri |
| MOL004328 | naringenin | C27H30O16 | 272.27 | 480-41-1 | 439246 | Radix et Rhizoma Glycyrrhizae, Fructus Aurantii |
| MOL004355 | Spinasterol | C29H48O | 412.77 | 481-18-5 | 5281331 | Radix Paeoniae Rubra, Radix Platycodonis, Radix Achyranthis Bidentatae |
| MOL004368 | Hyperin | C21H20O12 | 464.41 | 482-36-0 | 5281643 | Radix Bupleuri, Radix Achyranthis Bidentatae, |
| MOL004385 | Yinyanghuo D | C20H18O5 | 338.38 | 119240-82-3 | 5315396 | Radix et Rhizoma Glycyrrhizae |
| MOL004474 | Maruzen M | C8H10O | 122.18 | 29471-88-3 | 31242 | Radix Angelicae Sinensis |
| MOL004479 | o-cresol | C7H8O | 108.15 | 95-48-7 | 335 | Radix Angelicae Sinensis |
| MOL004580 | cis-Dihydroquercetin | C15H12O7 | 304.27 | 480-18-2 | 443758 | Radix Platycodonis |
| MOL004582 | Methyl naphthalene | C11H10 | 142.21 | 78900-94-4 | 7002 | Radix Bupleuri |
| MOL004585 | 2,4-Dodecadienal | C12H20O | 180.32 | 21662-16-8 | 5367530 | Radix Bupleuri |
| MOL004587 | Heptenoic acid | C7H12O2 | 128.19 | 25377-46-2 | 5282709 | Radix Bupleuri |
| MOL004589 | Methylheptane | C8H18 | 114.26 | 592-27-8 | 11594 | Radix Bupleuri, Radix et Rhizoma Glycyrrhizae |
| MOL004591 | Nonenoic acid | C9H16O2 | 156.25 | 29830-11-3 | 5312586 | Radix Bupleuri |
| MOL004592 | 2-Octenic acid | C8H14O2 | 142.22 | 1871-67-6 | 5282714 | Radix Bupleuri |
| MOL004598 | 3,5,6,7-tetramethoxy-2-(3,4,5-trimethoxyphenyl) chromone | C22H24O9 | 432.46 | 17245-30-6 | 389001 | Radix Bupleuri |
| MOL004599 | 3,3,5-trimethylheptane | C10H22 | 142.32 | 7154-80-5 | 23544 | Radix Bupleuri |
| MOL004602 | (3R)-3-methylcyclotridecan-1-one | C14H26O | 210.4 | 61415-11-0 | 12002600 | Radix Bupleuri |
| MOL004603 | 3-ethyl-2-methyl-1,3-hexadiene | C9H16 | 124.25 | 61142-36-7 | 5365710 | Radix Bupleuri |
| MOL004604 | Heptan-3-on | C7H14O | 114.21 | 106-35-4 | 7802 | Radix Bupleuri |
| MOL004608 | (5S)-5-butyloxolan-2-one | C8H14O2 | 142.22 | 19340-56-8 | 7057972 | Radix Bupleuri |
| MOL004609 | Areapillin | C18H16O8 | 360.34 | 83162-82-7 | 158311 | Radix Bupleuri |
| MOL004610 | gamma-Undecalactone | C11H20O2 | 184.31 | 104-67-6 | 441644 | Radix Bupleuri |
| MOL004613 | Riligustilide | C24H28O4 | 380.52 | 89354-45-0 | 6442656 | Radix Bupleuri, Radix Angelicae Sinensis |
| MOL004616 | 7-Octen-4-ol | C8H16O | 128.24 | 53907-72-5 | 40923 | Radix Bupleuri |
| MOL004617 | Ayapanin | C10H8O3 | 176.18 | 531-59-9 | 10748 | Radix Bupleuri |
| MOL004619 | 8-nonenoic acid | C9H16O2 | 156.25 | 31642-67-8 | 35860 | Radix Bupleuri |
| MOL004623 | Encecalin | C14H16O3 | 232.3 | 20628-09-5 | 114703 | Radix Bupleuri |
| MOL004624 | Longikaurin A | C20H28O5 | 348.48 | 75207-67-9 | 433636 | Radix Bupleuri |
| MOL004626 | Longispinogenin | C30H50O3 | 458.8 | 465-94-1 | 23641100 | Radix Bupleuri |
| MOL004627 | Methyl hexoate | C7H14O2 | 130.21 | 106-70-7 | 7824 | Radix Bupleuri |
| MOL004629 | 2-Phenylacetic acid | C8H8O2 | 136.16 | 103-82-2 | 999 | Radix Bupleuri |
| MOL004631 | 7,8,4'-Trihydroxyisoflavone | C15H10O5 | 270.25 | 75187-63-2 | 5466139 | Radix Bupleuri |
| MOL004632 | Pulsatillic acid | C30H46O4 | 470.76 | Not Available | 10672073 | Radix Bupleuri |
| MOL004634 | Saikogenin G | C30H48O4 | 472.78 | 18175-79-6 | 21594258 | Radix Bupleuri |
| MOL004635 | saikosaponin a | C42H681O13 | 781.1 | 20736-09-8 | Not Available | Radix Bupleuri |
| MOL004647 | N-Tridecanoic acid | C13H26O2 | 214.39 | 638-53-9 | 12530 | Radix Bupleuri |
| MOL004650 | Acetovanillin | C10H10O4 | 194.2 | 881-68-5 | 61229 | Radix Bupleuri |
| MOL004651 | cis-2-Undecenal | C11H20O | 168.31 | 68820-32-6 | 6431019 | Radix Bupleuri |
| MOL004653 | (+)-Anomalin | C24H26O7 | 426.5 | 73069-28-0 | 5319252 | Radix Bupleuri |
| MOL004656 | (5R)-5-isopropyl-2-methyl-1-cyclohex-2-enone | C10H16O | 152.26 | 499-71-8 | 10888107 | Radix Bupleuri |
| MOL004657 | caryophyllene oxide | C15H24O | 220.39 | 1139-30-6 | 1742210 | Radix Bupleuri |
| MOL004658 | cedrenol | C15H24O | 220.39 | 28231-03-0 | 21119906 | Radix Bupleuri |
| MOL004661 | [(3R)-3,7-dimethyloct-6-enyl] acetate | C12H22O2 | 198.34 | 150-84-5 | 6708682 | Radix Bupleuri |
| MOL004662 | Cyclohexylisocyanate | C7H11NO | 125.19 | 3173-53-3 | 18502 | Radix Bupleuri |
| MOL004665 | Ethyl geranate | C12H20O2 | 196.32 | 13058-12-3 | 5317247 | Radix Bupleuri |
| MOL004666 | Ethyl protocatechuate | C9H10O4 | 182.19 | 3943-89-3 | 77547 | Radix Bupleuri |
| MOL004667 | fraxetin | C10H8O5 | 208.18 | 574-84-5 | 5273569 | Radix Bupleuri |
| MOL004668 | Beta-D-galactopyranose | C6H12O6 | 180.18 | 7296-64-2 | 439353 | Radix Bupleuri |
| MOL004671 | Isopulegol | C10H18O | 154.28 | 50373-36-9 | 170833 | Radix Bupleuri |
| MOL004673 | kaempferitrin | C27H30O14 | 578.57 | 482-38-2 | 5486199 | Radix Bupleuri |
| MOL004675 | kaempferol-7-O-rhamnoside | C21H20O10 | 432.41 | 20196-89-8 | 53891100 | Radix Bupleuri |
| MOL004677 | ledol | C15H26O | 222.41 | 489-41-8 | Not Available | Radix Bupleuri |
| MOL004678 | Limetin | C11H10O4 | 206.21 | 487-06-9 | 2775 | Radix Bupleuri |
| MOL004679 | longifolene | C15H24 | 204.39 | 475-20-7 | 5489258 | Radix Bupleuri |
| MOL004680 | cis-p-2-Menthen-1-ol | C10H18O | 154.28 | 29803-82-5 | 122485 | Radix Bupleuri |
| MOL004682 | Methyl octylate | C9H18O2 | 158.27 | 111-11-5 | 8091 | Radix Bupleuri |
| MOL004683 | methyl (2E,4E)-octadeca-2,4-dienoate | C19H34O2 | 294.53 | 28061-47-4 | 6442145 | Radix Bupleuri |
| MOL004687 | 2-octanone | C8H16O | 128.24 | 111-13-7 | 8093 | Radix Bupleuri |
| MOL004688 | Cumic acid | C10H12O2 | 164.22 | 536-66-3 | 10820 | Radix Bupleuri |
| MOL004702 | saikosaponin c_qt | Not Available | 472.78 | 20736-08-7 | Not Available | Radix Bupleuri |
| MOL004705 | (3E,6S,7R)-3-butylidene-6,7-dihydroxy-4,5,6,7-tetrahydroisobenzofuran-1-one | C12H16O4 | 224.28 | 94596-27-7 | 10036567 | Radix Bupleuri |
| MOL004706 | ZINC33961943 | C15H24O | 220.39 | 6750-60-3 | 9794468 | Radix Bupleuri |
| MOL004707 | tau-cadinol | C15H26O | 222.41 | Not Available | 12302227 | Radix Bupleuri |
| MOL004708 | δ-cadinol | C15H26O | 222.41 | 481-34-5 | 51394521 | Radix Bupleuri |
| MOL004709 | (+)-trans-Carveol | C10H16O | 152.26 | 99-48-9 | 443178 | Radix Bupleuri |
| MOL004710 | (2R)-2-azaniumyl-3-(1H-indol-3-yl)propanoate | C11H12N2O2 | 204.25 | 153-94-6 | 6923517 | Radix Bupleuri |
| MOL004713 | alpha-Eudesmol | C15H26O | 222.41 | 473-16-5 | 92762 | Radix Bupleuri |
| MOL004714 | (1R,4S)-7,7-dimethyl-2-methylenenorbornane | C10H16 | 136.26 | 471-84-1 | 10887972 | Radix Bupleuri |
| MOL004715 | (4aS,9aS)-2,9,9-trimethyl-5-methylene-4,4a,6,7, 8,9a-hexahydro-3H-benzo[7]annulene | C15H26O | 204.39 | 3853-83-6 | 24798713 | Radix Bupleuri |
| MOL004716 | (1S,4R)-2,3-dimethylbicyclo[2.2.1]hept-2-ene | C9H14 | 122.23 | 529-16-8 | 11457687 | Radix Bupleuri |
| MOL004717 | (3R,4aR,8aR)-3-isopropenyl-5,8a-dimethyl-2,3,4, 4a,7,8-hexahydro-1H-naphthalene | C15H24 | 204.39 | 473-13-2 | 10856614 | Radix Bupleuri |
| MOL004718 | α-spinasterol | C29H48O | 412.77 | 481-18-5 | 5281331 | Radix Bupleuri |
| MOL004720 | β-cedrene | C15H24 | 204.39 | 546-28-1 | 11106485 | Radix Bupleuri |
| MOL004721 | β-fenchene | C10H16 | 136.26 | Not Available | 12309844 | Radix Bupleuri |
| MOL004722 | β-oplopenone | C15H24O | 220.39 | 28305-60-4 | 14038847 | Radix Bupleuri |
| MOL004723 | beta-Terpinene | C10H16 | 136.26 | 99-84-3 | 66841 | Radix Bupleuri, Radix Angelicae Sinensis, Radix et Rhizoma Glycyrrhizae |
| MOL004725 | (5S)-5-hexyloxolan-2-one | C10H18O2 | 170.28 | 706-14-9 | 7057904 | Radix Bupleuri |
| MOL004726 | (5S)-5-propyloxolan-2-one | C7H12O2 | 128.19 | 105-21-5 | 5325912 | Radix Bupleuri |
| MOL004727 | (1R,4aR,8aS)-1-isopropyl-7-methyl-4-methylene-2,3,4a,5,6,8a-hexahydro-1H-naphthalene | C15H24 | 204.39 | 30021-74-0 | 6432308 | Radix Bupleuri |
| MOL004728 | γ-patchoulene | C15H24 | 204.39 | 508-55-4 | 521302 | Radix Bupleuri |
| MOL004802 | (E)-1-butoxyhex-2-ene | C10H20O | 156.3 | 54340-67-9 | 5364961 | Radix et Rhizoma Glycyrrhizae |
| MOL004804 | 18beta-glycyrrhetinic acid | C30H46O4 | 470.76 | 471-53-4 | 10114 | Radix et Rhizoma Glycyrrhizae |
| MOL004805 | Semilicoisoflavone | C25H26O4 | 390.51 | 157414-03-4 | 197678 | Radix et Rhizoma Glycyrrhizae |
| MOL004806 | euchrenone | Not Available | 406.56 | Not Available | Not Available | Radix et Rhizoma Glycyrrhizae |
| MOL004807 | glucuronic acid | C6H10O7 | 194.16 | 576-37-4 | 441478 | Radix et Rhizoma Glycyrrhizae |
| MOL004808 | glyasperin B | C21H22O6 | 370.43 | 142488-54-8 | 480784 | Radix et Rhizoma Glycyrrhizae |
| MOL004810 | glyasperin F | C20H18O6 | 354.38 | Not Available | 392442 | Radix et Rhizoma Glycyrrhizae |
| MOL004811 | Glyasperin C | C21H24O5 | 356.45 | 142474-53-1 | 480859 | Radix et Rhizoma Glycyrrhizae |
| MOL004814 | Isotrifoliol | C16H10O6 | 298.26 | Not Available | 5318679 | Radix et Rhizoma Glycyrrhizae |
| MOL004815 | Kanzonol B | C20H18O4 | 322.38 | Not Available | 10881804 | Radix et Rhizoma Glycyrrhizae |
| MOL004820 | kanzonols W | C20H16O5 | 336.36 | Not Available | 15380912 | Radix et Rhizoma Glycyrrhizae |
| MOL004824 | (2S)-6-(2,4-dihydroxyphenyl)-2-(2-hydroxypropan-2-yl)-4-methoxy-2,3-dihydrofuro [3,2-g]chromen-7-one | C21H20O7 | 384.41 | Not Available | 637112 | Radix et Rhizoma Glycyrrhizae |
| MOL004827 | Semilicoisoflavone B | C20H16O6 | 352.36 | 129280-33-7 | 5481948 | Radix et Rhizoma Glycyrrhizae |
| MOL004828 | Glepidotin A | C20H18O5 | 338.38 | 42193-83-9 | 5281619 | Radix et Rhizoma Glycyrrhizae |
| MOL004829 | Glepidotin B | C20H20O5 | 340.4 | 87440-56-0 | 442411 | Radix et Rhizoma Glycyrrhizae |
| MOL004830 | Octadiene | C8H14 | 110.22 | 63597-41-1 | 517653 | Radix et Rhizoma Glycyrrhizae |
| MOL004832 | N-Butyl benzoate | C11H14O2 | 178.25 | 136-60-7 | 8698 | Radix et Rhizoma Glycyrrhizae |
| MOL004833 | Phaseolinisoflavan | C20H20O4 | 324.4 | 40323-57-7 | 162412 | Radix et Rhizoma Glycyrrhizae |
| MOL004835 | Glypallichalcone | C17H16O4 | 284.33 | 146763-58-8 | 5317768 | Radix et Rhizoma Glycyrrhizae |
| MOL004836 | echinatin | C16H14O4 | 270.3 | 34221-41-5 | 6442675 | Radix et Rhizoma Glycyrrhizae |
| MOL004837 | Karenzu DK2 | C15H12O2 | 224.27 | 61346-73-4 | 8433 | Radix et Rhizoma Glycyrrhizae |
| MOL004838 | Glabrocoumarone A | C19H16O4 | 308.35 | Not Available | 10542808 | Radix et Rhizoma Glycyrrhizae |
| MOL004839 | (1S,2S)-1,2-dimethylcyclopentane | C7H14 | 98.21 | 822-50-4 | 641612 | Radix et Rhizoma Glycyrrhizae |
| MOL004841 | Licochalcone B | C16H14O5 | 286.3 | 58749-23-8 | 5318999 | Radix et Rhizoma Glycyrrhizae |
| MOL004847 | 2,2-DIMETHYLPENTANE | C7H16 | 100.23 | 590-35-2 | 11542 | Radix et Rhizoma Glycyrrhizae |
| MOL004848 | licochalcone G | C21H22O5 | 354.43 | Not Available | 49856081 | Radix et Rhizoma Glycyrrhizae |
| MOL004849 | Licoarylcoumarin | C21H20O6 | 368.41 | Not Available | 10090416 | Radix et Rhizoma Glycyrrhizae |
| MOL004853 | Licoflavonol | C20H18O6 | 354.38 | 60197-60-6 | 5481964 | Radix et Rhizoma Glycyrrhizae |
| MOL004855 | Licoricone | C22H22O6 | 382.44 | 51847-92-8 | 5319013 | Radix et Rhizoma Glycyrrhizae |
| MOL004856 | Gancaonin A | C21H20O5 | 352.41 | 27762-99-8 | 5317478 | Radix et Rhizoma Glycyrrhizae |
| MOL004857 | Gancaonin B | C21H20O6 | 368.41 | 124596-86-7 | 5317479 | Radix et Rhizoma Glycyrrhizae |
| MOL004859 | 2,3-dimethylhexane | C8H18 | 114.26 | 584-94-1 | 24884354 | Radix et Rhizoma Glycyrrhizae |
| MOL004863 | Gancaonin L | C20H18O6 | 354.38 | 129145-50-2 | 14604077 | Radix et Rhizoma Glycyrrhizae |
| MOL004864 | Gancaonin M | C21H20O5 | 352.41 | 129145-51-3 | 14604078 | Radix et Rhizoma Glycyrrhizae |
| MOL004866 | 2-(3,4-dihydroxyphenyl)-5,7-dihydroxy-6-(3-methylbut-2-enyl)chromone | C20H18O6 | 354.38 | 129145-53-5 | 14604081 | Radix et Rhizoma Glycyrrhizae |
| MOL004871 | (3S)-2,3-dimethylpentane | C7H16 | 100.23 | 565-59-3 | 22810194 | Radix et Rhizoma Glycyrrhizae |
| MOL004879 | Glycyrin | C22H22O6 | 382.44 | 66056-18-6 | 480787 | Radix et Rhizoma Glycyrrhizae |
| MOL004880 | 5,6,7,8-Tetrahydro-2,4-dimethylquinoline | C11H15N | 161.27 | 60169-66-6 | 5321849 | Radix et Rhizoma Glycyrrhizae |
| MOL004881 | MORACHALCONE A | C20H20O5 | 340.4 | 76472-88-3 | 9862769 | Radix et Rhizoma Glycyrrhizae |
| MOL004882 | Licocoumarone | 20H20O5 | 340.4 | 118524-14-4 | 503731 | Radix et Rhizoma Glycyrrhizae |
| MOL004883 | Licoisoflavone A | C20H18O6 | 354.38 | 66056-19-7 | 5281789 | Radix et Rhizoma Glycyrrhizae |
| MOL004884 | Licoisoflavone B | C20H16O6 | 352.36 | 66056-30-2 | 5481234 | Radix et Rhizoma Glycyrrhizae |
| MOL004885 | licoisoflavanone | C20H18O6 | 354.38 | 66067-26-3 | 392443 | Radix et Rhizoma Glycyrrhizae |
| MOL004890 | (4S)-2,4-dimethylhexane | C8H18 | 114.26 | 589-43-5 | 22810193 | Radix et Rhizoma Glycyrrhizae |
| MOL004891 | shinpterocarpin | C20H18O4 | 322.38 | 157414-04-5 | 10336244 | Radix et Rhizoma Glycyrrhizae |
| MOL004898 | 5-Prenylbutein | C20H20O5 | 340.4 | Not Available | 11267805 | Radix et Rhizoma Glycyrrhizae |
| MOL004903 | liquiritin | C21H22O9 | 418.43 | 31564-20-2 | 503737 | Radix et Rhizoma Glycyrrhizae |
| MOL004904 | licopyranocoumarin | C21H20O7 | 384.41 | 117038-80-9 | 122851 | Radix et Rhizoma Glycyrrhizae |
| MOL004907 | Glyzaglabrin | C16H10O6 | 298.26 | Not Available | 5317777 | Radix et Rhizoma Glycyrrhizae |
| MOL004908 | Glabridin | C20H20O4 | 324.4 | 59870-68-7 | 124052 | Radix et Rhizoma Glycyrrhizae |
| MOL004910 | Glabranin | C20H20O4 | 324.4 | 41983-91-9 | 124049 | Radix et Rhizoma Glycyrrhizae |
| MOL004911 | Glabrene | C20H18O4 | 322.38 | 60008-03-9 | 480774 | Radix et Rhizoma Glycyrrhizae |
| MOL004912 | Glabrone | C20H16O5 | 336.36 | 60008-02-8 | 5317652 | Radix et Rhizoma Glycyrrhizae |
| MOL004913 | Hedysarimcoumestan B | C16H10O6 | 298.26 | Not Available | 11558452 | Radix et Rhizoma Glycyrrhizae |
| MOL004914 | 1,3-dihydroxy-8,9-dimethoxy-6-benzofurano[3,2-c]chromenone | C17H12O7 | 328.29 | Not Available | 11602329 | Radix et Rhizoma Glycyrrhizae |
| MOL004915 | Eurycarpin A | C20H18O5 | 338.38 | 166547-20-2 | 5317300 | Radix et Rhizoma Glycyrrhizae |
| MOL004919 | Sextone B | C7H14 | 98.21 | 108-87-2 | 7962 | Radix et Rhizoma Glycyrrhizae |
| MOL004920 | Methylcyclopentane | C6H12 | 84.18 | 5310-57-6 | 7296 | Radix et Rhizoma Glycyrrhizae |
| MOL004924 | (-)-Medicocarpin | C22H24O9 | 432.46 | Not Available | 23724664 | Radix et Rhizoma Glycyrrhizae |
| MOL004925 | vitexin | C21H20O10 | 432.41 | 521-33-5 | 5280441 | Radix et Rhizoma Glycyrrhizae |
| MOL004926 | 4H-1-Benzopyran-4-one, 2-(4-(beta-D-glucopyran-osyloxy)phenyl)-2,3-dihydro-5,7-dihydroxy-, (2S)- | C21H22O10 | 434.43 | 81202-36-0 | 157745 | Radix et Rhizoma Glycyrrhizae |
| MOL004932 | glycyrrhizin | C42H62O16 | 823.04 | 103000-77-7 | 128229 | Radix et Rhizoma Glycyrrhizae |
| MOL004934 | Isohexane | C6H14 | 86.2 | 73513-42-5 | 7892 | Radix et Rhizoma Glycyrrhizae |
| MOL004935 | Sigmoidin-B | C20H20O6 | 356.4 | 87746-47-2 | 73205 | Radix et Rhizoma Glycyrrhizae |
| MOL004936 | Uralene | C21H20O7 | 384.41 | 150853-99-9 | 192490 | Radix et Rhizoma Glycyrrhizae |
| MOL004940 | neoliquiritin | C21H22O9 | 418.43 | 5088-75-5 | 51666249 | Radix et Rhizoma Glycyrrhizae |
| MOL004941 | (2R)-7-hydroxy-2-(4-hydroxyphenyl)chroman-4-one | C15H12O4 | 256.27 | 5088-75-5 | 928837 | Radix et Rhizoma Glycyrrhizae |
| MOL004944 | Cyclobutanol, 1-ethyl- | C6H12O | 100.18 | 84256-19-9 | 145025 | Radix et Rhizoma Glycyrrhizae |
| MOL004945 | (2S)-7-hydroxy-2-(4-hydroxyphenyl)-8-(3-methylbut-2-enyl)chroman-4-one | C20H20O4 | 324.4 | 31524-62-6 | 193679 | Radix et Rhizoma Glycyrrhizae |
| MOL004948 | Isoglycyrol | C21H18O6 | 366.39 | 23013-86-7 | 124050 | Radix et Rhizoma Glycyrrhizae |
| MOL004949 | Isolicoflavonol | C20H18O6 | 354.38 | 94805-83-1 | 5318585 | Radix et Rhizoma Glycyrrhizae |
| MOL004957 | Isoformononetin | C16H12O4 | 268.28 | 486-63-5 | 3764 | Radix et Rhizoma Glycyrrhizae |
| MOL004959 | 1-Methoxyphaseollidin | C21H22O5 | 354.43 | Not Available | 480873 | Radix et Rhizoma Glycyrrhizae |
| MOL004961 | Quercetin der. | C17H14O7 | 330.31 | 4382-17-6 | 5316900 | Radix et Rhizoma Glycyrrhizae |
| MOL004963 | 24-Hydroxyglycyrrhetic acid | Not Available | 486.76 | Not Available | Not Available | Radix et Rhizoma Glycyrrhizae |
| MOL004964 | (Z)-1-(2,4-dihydroxyphenyl)-3-phenylprop-2-en-1-one | C15H12O3 | 240.27 | Not Available | 10331849 | Radix et Rhizoma Glycyrrhizae |
| MOL004966 | 3'-Hydroxy-4'-O-Methylglabridin | C21H22O5 | 354.43 | Not Available | 15228662 | Radix et Rhizoma Glycyrrhizae |
| MOL004967 | 3,3-Dimethylpentane | C7H16 | 100.23 | 562-49-2 | 11229 | Radix et Rhizoma Glycyrrhizae |
| MOL004969 | 2-Ethyl-p-xylene | C10H14 | 134.24 | 1758-88-9 | 15653 | Radix et Rhizoma Glycyrrhizae |
| MOL004970 | 3-methylheptane | C8H18 | 114.26 | 589-81-1 | 12263096 | Radix et Rhizoma Glycyrrhizae |
| MOL004971 | 3-methylhexane | C7H16 | 100.23 | 589-34-4 | 13800357 | Radix et Rhizoma Glycyrrhizae |
| MOL004972 | 3-Methylpentane | C6H14 | 86.2 | 96-14-0 | 7282 | Radix et Rhizoma Glycyrrhizae |
| MOL004973 | 3-Ethylpentane | C7H16 | 100.23 | 617-78-7 | 12048 | Radix et Rhizoma Glycyrrhizae |
| MOL004974 | 3'-Methoxyglabridin | C21H22O5 | 354.43 | Not Available | 15228663 | Radix et Rhizoma Glycyrrhizae |
| MOL004978 | 4'-methoxyglabridin | C21H22O4 | 338.43 | Not Available | 9927807 | Radix et Rhizoma Glycyrrhizae |
| MOL004980 | Inflacoumarin A | C20H18O4 | 322.38 | 158446-33-4 | 5318437 | Radix et Rhizoma Glycyrrhizae |
| MOL004982 | 2,6,10-trimethyl-dodecane | C6H8O4 | 144.14 | 28564-83-2 | 119838 | Radix et Rhizoma Glycyrrhizae |
| MOL004983 | 5,6,7,8-Tetrahydro-4-methylquinoline | C10H13N | 147.24 | 28971-03-1 | 185667 | Radix et Rhizoma Glycyrrhizae |
| MOL004985 | icos-5-enoic acid | C20H38O2 | 310.58 | Not Available | 5312521 | Radix et Rhizoma Glycyrrhizae |
| MOL004987 | 11-deoxyglycyrrhetic acid | C30H48O3 | 456.78 | 564-16-9 | 12305517 | Radix et Rhizoma Glycyrrhizae |
| MOL004988 | Kanzonol F | C26H28O5 | 420.54 | Not Available | 101666840 | Radix et Rhizoma Glycyrrhizae |
| MOL004989 | 6-prenylated eriodictyol | Not Available | 356.4 | Not Available | Not Available | Radix et Rhizoma Glycyrrhizae |
| MOL004990 | 7,2',4'-trihydroxy－5-methoxy-3－arylcoumarin | C16H12O6 | 300.28 | Not Available | 25015742 | Radix et Rhizoma Glycyrrhizae |
| MOL004991 | 7-Acetoxy-2-methylisoflavone | C18H14O4 | 294.32 | 3211-63-0 | 268208 | Radix et Rhizoma Glycyrrhizae |
| MOL004993 | 8-prenylated eriodictyol | Not Available | 356.4 | Not Available | Not Available | Radix et Rhizoma Glycyrrhizae |
| MOL004996 | gadelaidic acid | C20H38O2 | 310.58 | 506-31-0 | 5460988 | Radix et Rhizoma Glycyrrhizae |
| MOL005000 | Gancaonin G | C21H20O5 | 352.41 | 126716-34-5 | 480780 | Radix et Rhizoma Glycyrrhizae |
| MOL005001 | Gancaonin H | C25H24O6 | 420.49 | 126716-35-6 | 5481949 | Radix et Rhizoma Glycyrrhizae |
| MOL005003 | Licoagrocarpin | C21H22O4 | 338.43 | Not Available | 15840593 | Radix et Rhizoma Glycyrrhizae |
| MOL005007 | Glyasperin M | C21H20O6 | 368.41 | 156162-05-9 | 101664572 | Radix et Rhizoma Glycyrrhizae |
| MOL005008 | Glycyrrhiza flavonol A | C20H18O7 | 370.38 | Not Available | 5317765 | Radix et Rhizoma Glycyrrhizae |
| MOL005012 | Licoagroisoflavone | C20H16O5 | 336.36 | Not Available | 636883 | Radix et Rhizoma Glycyrrhizae |
| MOL005013 | 18α-hydroxyglycyrrhetic acid | Not Available | 486.76 | 17991-67-2 | Not Available | Radix et Rhizoma Glycyrrhizae |
| MOL005016 | Odoratin | C17H14O6 | 314.31 | Not Available | 13965473 | Radix et Rhizoma Glycyrrhizae |
| MOL005017 | Phaseol | C20H16O5 | 336.36 | Not Available | 44257530 | Radix et Rhizoma Glycyrrhizae |
| MOL005018 | Xambioona | C25H24O4 | 388.49 | 82345-36-6 | 73352581 | Radix et Rhizoma Glycyrrhizae |
| MOL005020 | dehydroglyasperins C | C21H22O5 | 354.396 | Not Available | 480775 | Radix et Rhizoma Glycyrrhizae |
| MOL005021 | Mipax | C10H10O4 | 194.2 | 131-11-3 | 8554 | Radix et Rhizoma Glycyrrhizae |
| MOL005125 | P-Anisic acid | C8H8O3 | 152.16 | 100-09-4 | 7478 | Radix Angelicae Sinensis |
| MOL005155 | ginsenoside Ro_qt | C30H48O3 | 455.77 | 7518-22-1 | 7055468 | Radix Achyranthis Bidentatae |
| MOL005449 | L-Met | C5H11NO2S | 149.24 | 26062-47-5 | 6992087 | Radix Angelicae Sinensis |
| MOL005589 | 3,4-dimethylbenzaldehyde | C9H10O | 134.19 | 5973-71-7 | 22278 | Radix Angelicae Sinensis |
| MOL005590 | Ethylbenzaldehyde | C9H10O | 134.19 | 4748-78-1 | 20861 | Radix Angelicae Sinensis |
| MOL005608 | 2,3,5-trimethylbenzene-1,4-diol | C9H12O2 | 152.21 | 700-13-0 | 12785 | Radix Angelicae Sinensis |
| MOL005812 | naringin | C27H32O14 | 580.59 | 10236-47-2 | 442428 | Radix et Rhizoma Glycyrrhizae, Fructus Aurantii |
| MOL005828 | nobiletin | C21H22O8 | 402.43 | 478-01-3 | 72344 | Fructus Aurantii |
| MOL005990 | 1-methoxy-1,2-butadiene | C5H8O | 84.13 | Not Available | 57109763 | Radix Platycodonis |
| MOL005991 | ISOPROPYL FORMATE | C4H8O2 | 88.12 | 625-55-8 | 12257 | Radix Platycodonis |
| MOL005992 | 2,3-dimethyl-1-pentene | C7H14 | 98.21 | 3404-72-6 | 12927329 | Radix Platycodonis |
| MOL005994 | 2-hydroxybicyclo[3.1.1]heptan-6-one | Not Available | 126.17 | Not Available | Not Available | Radix Platycodonis |
| MOL005999 | crotonaldehyde | C4H6O | 70.1 | 123-73-9 | 447466 | Radix Platycodonis |
| MOL006002 | 3,4-Heptadiene | C7H12 | 96.19 | 2454-31-1 | 556860 | Radix Platycodonis |
| MOL006003 | (1S,5R)-3,7,7-trimethylbicyclo[3.1.1]hept-3-ene | C10H16 | 136.26 | Not Available | 11240513 | Radix Platycodonis |
| MOL006005 | allyloxyethylene | C5H8O | 84.13 | 3917-15-5 | 221523 | Radix Platycodonis |
| MOL006006 | 3-ethyl-1,4-hexadiene | C8H14 | 110.22 | 2080-89-9 | 6433321 | Radix Platycodonis |
| MOL006007 | 3-ethyl-cyclohexene | Not Available | 110.22 | Not Available | 13077038 | Radix Platycodonis |
| MOL006023 | α-Spinasterol-β-D-glucoside_qt | Not Available | 412.77 | Not Available | Not Available | Radix Platycodonis |
| MOL006028 | dimethyl 3-O-β-D-glucopyranosylplatycogenate A | Not Available | 546.82 | Not Available | Not Available | Radix Platycodonis |
| MOL006029 | dimethyl platyconate A | Not Available | 562.82 | Not Available | Not Available | Radix Platycodonis |
| MOL006031 | Grandoside | C17H32O11 | 412.49 | Not Available | 10341593 | Radix Platycodonis |
| MOL006035 | lobetyolin | C20H28O8 | 396.48 | 136085-37-5 | 53486204 | Radix Platycodonis |
| MOL006039 | Methyl 3-methyl-2-pentenoate | C7H12O2 | 128.19 | 50652-79-4 | 5362896 | Radix Platycodonis |
| MOL006041 | methyl 3-O-β-D-glucopyranosyl polygalacate _qt | Not Available | 518.81 | Not Available | Not Available | Radix Platycodonis |
| MOL006047 | methylbutyl-1,2-benzenedicarboxylate | C13H16O4 | 236.29 | Not Available | 70153369 | Radix Platycodonis |
| MOL006069 | stigmastenol | Not Available | 412.77 | Not Available | Not Available | Radix Platycodonis |
| MOL006071 | (1R,2R,3R,5R)-5-(((1R,2R,4R,5S)-5-hydroxy-4-(hydroxymethyl)-2-(isopentylperoxy)cyclohexyl) oxy)-3-(hydroxymethyl)cyclohexane-1,2-diol | Not Available | 392.55 | Not Available | Not Available | Radix Platycodonis |
| MOL006077 | Thiamine | C12H18N4OS | 265.4 | 59-43-8 | 9543525 | Radix Platycodonis |
| MOL006179 | 2-(6-carboxy-2,3,4-trihydroxyphenyl)-3,4,5-trihydroxybenzoic acid | C14H10O10 | 338.24 | 517-46-4 | 10315050 | Radix Paeoniae Rubra |
| MOL006731 | Areginal | C3H6O2 | 74.09 | 109-94-4 | 8025 | Radix Achyranthis Bidentatae |
| MOL006765 | peonidin | C16H14O6 | 301.29 | 134-01-0 | Not Available | Radix Paeoniae Rubra |
| MOL006869 | 4-Acetylresorcinol | C8H8O3 | 152.16 | 89-84-9 | 6990 | Radix Angelicae Sinensis |
| MOL006991 | Endo-2-Cineolylol | C10H18O2 | 170.28 | 60761-00-4 | 109010 | Radix Paeoniae Rubra |
| MOL006992 | (2R,3R)-4-methoxyl-distylin | Not Available | 318.3 | Not Available | Not Available | Radix Paeoniae Rubra |
| MOL006994 | 1-o-beta-d-glucopyranosyl-8-o-benzoyl-paeonisuffrone_qt | Not Available | 302.35 | Not Available | Not Available | Radix Paeoniae Rubra |
| MOL006996 | 1-o-beta-d-glucopyranosylpaeonisuffrone_qt | Not Available | 332.38 | Not Available | Not Available | Radix Paeoniae Rubra |
| MOL006997 | 2-[(2R,5R,6R)-6,10-dimethylspiro[4.5]dec-9-en-2-yl] propan-2-ol | C15H26O | 222.41 | 1460-73-7 | 21675005 | Radix Paeoniae Rubra |
| MOL006999 | stigmast-7-en-3-ol | C29H50O | 414.79 | 18525-35-4 | 12315376 | Radix Paeoniae Rubra |
| MOL007000 | 2-methoxy-5-(e)-propenyl-phenol-beta-vicianoside | Not Available | 458.51 | Not Available | Not Available | Radix Paeoniae Rubra |
| MOL007001 | 2-methoxy-5-[(Z)-prop-1-enyl]phenol | C10H12O2 | 164.22 | Not Available | 1781945 | Radix Paeoniae Rubra |
| MOL007005 | Albiflorin_qt | Not Available | 318.35 | Not Available | Not Available | Radix Paeoniae Rubra |
| MOL007015 | 8-debenzoylpaeonidanin_qt | Not Available | 228.27 | Not Available | Not Available | Radix Paeoniae Rubra |
| MOL007016 | Paeoniflorigenone | C17H18O6 | 318.35 | 80454-42-8 | 70698143 | Radix Paeoniae Rubra |
| MOL007018 | 9-ethyl-neo-paeoniaflorin A_qt | Not Available | 334.4 | Not Available | Not Available | Radix Paeoniae Rubra |
| MOL007020 | Lactiflorin_qt | Not Available | 318.35 | Not Available | Not Available | Radix Paeoniae Rubra |
| MOL007026 | Paeonin A | C17H24O9 | 372.41 | Not Available | 44233203 | Radix Paeoniae Rubra |
| MOL007027 | paeonin,a_qt | Not Available | 210.25 | Not Available | Not Available | Radix Paeoniae Rubra |
| MOL007028 | paeonin b | C16H22O9 | 358.38 | Not Available | 44233202 | Radix Paeoniae Rubra |
| MOL007029 | paeonin,b_qt | Not Available | 196.22 | Not Available | Not Available | Radix Paeoniae Rubra |
| MOL007030 | Paeonin C | C17H24O9 | 372.41 | Not Available | 44233425 | Radix Paeoniae Rubra |
| MOL007031 | paeonin,c_qt | Not Available | 210.25 | Not Available | Not Available | Radix Paeoniae Rubra |
| MOL007260 | Isorhamnetin-3-mono-beta-D-glucoside | C22H22O12 | 478.44 | 482-36-0 | 5318645 | Radix Bupleuri |
| MOL007561 | N-Methyltyramine | C9H13NO | 151.23 | 370-98-9 | 9727 | Fructus Aurantii |
| MOL007663 | ZINC43574872 | C15H22O10 | 362.37 | 2415-24-9 | 11810558 | Radix Rehmanniae |
| MOL007745 | WLN: QVR BVQ | C8H6O4 | 166.14 | 88-99-3 | 18183610 | Radix Angelicae Sinensis |
| MOL007891 | 2,6-Dimethylpiazine | C6H8N2 | 108.16 | 108-50-9 | 7938 | Radix Achyranthis Bidentatae |
| MOL007930 | hesperidin | C28H34O15 | 610.62 | 520-26-3 | 10621 | Fructus Aurantii |
| MOL008251 | sedanolide | C12H18O2 | 194.3 | 6415-59-4 | 12315451 | Radix Angelicae Sinensis |
| MOL008253 | sphingomyelin | Not Available | 493.73 | 85187-10-6 | Not Available | Radix Angelicae Sinensis |
| MOL008255 | α-acoradiene | C15H24 | 204.39 | 28400-13-7 | 6429151 | Radix Angelicae Sinensis |
| MOL008256 | InChI=1/C15H24/c1-10-7-8-15-9-12(10)14(3,4) 13(15)6-5-11(15)2/h7,11-13H,5-6,8-9H2,1-4H | C15H24 | 204.39 | 469-61-4 | Not Available | Radix Angelicae Sinensis |
| MOL008258 | (1R,4R,5S)-4-isopropenyl-1,8-dimethylspiro[4.5]dec-8-ene | C15H24 | 204.39 | Not Available | 13743810 | Radix Angelicae Sinensis |
| MOL008259 | 2,6-di(phenyl)thiopyran-4-thione | C17H12S2 | 280.43 | 1029-97-6 | 11832833 | Radix Angelicae Sinensis |
| MOL008260 | o-Xylenol | C8H10O | 122.18 | 526-75-0 | 10687 | Radix Angelicae Sinensis |
| MOL008262 | Mesitaldehyde | C10H12O | 148.22 | 487-68-3 | 10254 | Radix Angelicae Sinensis |
| MOL008263 | Isoxylaldehyde | C9H10O | 134.19 | 5779-94-2 | 22015 | Radix Angelicae Sinensis |
| MOL008265 | 2-valerylbenzoic acid | C12H14O3 | 206.26 | 64624-87-9 | 5315165 | Radix Angelicae Sinensis |
| MOL008273 | 4-Methyl-6-hepten-3-one | C8H14O | 126.22 | 26118-97-8 | 25018179 | Radix Angelicae Sinensis |
| MOL008274 | 6-Ethylresorcinol | C8H10O2 | 138.18 | 2896-60-8 | 17927 | Radix Angelicae Sinensis |
| MOL008277 | 7,10-pentadecadiynoic acid | C15H22O2 | 234.37 | 22117-06-2 | 30942 | Radix Angelicae Sinensis |
| MOL008281 | (Z)-2-[[(Z)-2-methylbut-2-enoyl]oxymethyl]but-2-enoic acid | C10H14O4 | 198.24 | 69188-40-5 | 6440421 | Radix Angelicae Sinensis |
| MOL008284 | Butyric acid | C4H8O2 | 88.12 | 107-92-6 | 264 | Radix Angelicae Sinensis |
| MOL008285 | (3S)-butylphthalide | C12H14O2 | 190.26 | 93133-67-6 | 11116832 | Radix Angelicae Sinensis |
| MOL008286 | ()-Camphoric acid | C10H16O4 | 200.26 | 560-09-8 | 101807 | Radix Angelicae Sinensis |
| MOL008287 | (3E)-3-butylidene-7-hydroxy-2-benzofuran-1-one | C12H12O3 | 204.24 | 103659-69-4 | 5852943 | Radix Angelicae Sinensis |
| MOL008288 | Coniferylferulate | C20H20O6 | 356.4 | 63644-62-2 | Not Available | Radix Angelicae Sinensis |
| MOL008291 | 2,4-Xylylaldehyde | C9H10O | 134.19 | 15764-16-6 | 61814 | Radix Angelicae Sinensis |
| MOL008292 | m-Ethylphenol | C8H10O | 122.18 | 620-17-7 | 12101 | Radix Angelicae Sinensis |
| MOL008293 | alpha.-Pyronene | C10H16 | 136.26 | 514-94-3 | 10581 | Radix Angelicae Sinensis |
| MOL008653 | Acetylfuran | C6H6O2 | 110.12 | 80145-44-4 | 14505 | Radix Achyranthis Bidentatae |
| MOL008671 | 2-isobutyl-3-methoxypyrazine | C9H14N2O | 166.25 | 24683-00-9 | 32594 | Radix Achyranthis Bidentatae |
| MOL008680 | acetaldehyde | C2H4O | 44.06 | 75-07-0 | 177 | Radix Achyranthis Bidentatae |
| MOL011390 | 3-epioleanolic acid | C30H48O3 | 456.78 | 25499-90-5 | 7061302 | Radix Achyranthis Bidentatae |
| MOL011404 | ginsenoside-Rg2_qt | Not Available | 476.82 | 52286-74-5 | Not Available | Radix Achyranthis Bidentatae |
| MOL011944 | 2-isopropyl-3-methoxypyrazine | C8H12N2O | 152.22 | 25773-40-4 | 33166 | Radix Achyranthis Bidentatae |
| MOL012297 | puerarin | C21H20O9 | 416.41 | 3681-99-0 | 5281807 | Radix Bupleuri |
| MOL012451 | (20r,22r)-2beta,3beta,20,22,26-pentahydroxy-cholestan-7,12-dien-6-one | Not Available | 462.69 | Not Available | Not Available | Radix Achyranthis Bidentatae |
| MOL012452 | (20R,22R)-2β,3β,20,22,26-pentahydroxy-cholestan-7,12-dien-6-one | Not Available | 462.69 | Not Available | Not Available | Radix Achyranthis Bidentatae |
| MOL012454 | 18-(β-D-Oxy glucose)-28-Oxo-12-oleanolic acid-3β-3-O-(β-D-glucose)-β-D-glucuronic acid methylester_qt | Not Available | 456.78 | Not Available | Not Available | Radix Achyranthis Bidentatae |
| MOL012455 | oct-1-en-2-ol | C8H16O | 128.24 | 142382-43-2 | 15372159 | Radix Achyranthis Bidentatae |
| MOL012456 | Inokosterone | C27H44O7 | 480.71 | 15130-85-5 | 441828 | Radix Achyranthis Bidentatae |
| MOL012460 | 28-deglucosyl-chikusetsusaponin,iva_qt | Not Available | 456.78 | Not Available | Not Available | Radix Achyranthis Bidentatae |
| MOL012461 | 28-norolean-17-en-3-ol | Not Available | 412.77 | Not Available | Not Available | Radix Achyranthis Bidentatae |
| MOL012463 | 2β,3β,20α,22α,25-pentahydroxy cholesta-8,14-dien-6-one | Not Available | 462.69 | Not Available | Not Available | Radix Achyranthis Bidentatae |
| MOL012465 | (3S,4aR,6aR,6bS,8aS,12aS,14aR,14bR)-4,4,6a,6b, 11,11,14b-heptamethyl-1,2,3,4a,5,6,7,8,9,10,12, 12a, 14,14a-tetradecahydropicene-3,8a-diol | C29H48O2 | 428.77 | Not Available | 21633445 | Radix Achyranthis Bidentatae |
| MOL012474 | achybidensaponin,ii_qt | Not Available | 456.78 | Not Available | Not Available | Radix Achyranthis Bidentatae |
| MOL012513 | deglucose chikusetsusaponin Iva_qt | Not Available | 456.78 | Not Available | Not Available | Radix Achyranthis Bidentatae |
| MOL012515 | Ecdysterone-3-O-beta-D-glucopyranoside_qt | Not Available | 466.68 | Not Available | Not Available | Radix Achyranthis Bidentatae |
| MOL012516 | geniposide | C17H24O10 | 388.41 | 24512-63-8 | 29927668 | Radix Achyranthis Bidentatae |
| MOL012518 | hederagenin-28-O-β-D-glucopyranosyl ester_qt | Not Available | 472.78 | Not Available | Not Available | Radix Achyranthis Bidentatae |
| MOL012523 | N-trans-feruloyl-3-methoxytyramine-4'-O-β-D-glucopyranoside | Not Available | 505.57 | Not Available | Not Available | Radix Achyranthis Bidentatae |
| MOL012524 | N-trans-feruloylmethoxytyramine | C19H21NO5 | 343.41 | 83608-86-0 | 5352115 | Radix Achyranthis Bidentatae |
| MOL012525 | N-trans-feruloyl-3-methoxytyramine-4-O-β-D-glucopyranoside | Not Available | 505.57 | Not Available | Not Available | Radix Achyranthis Bidentatae |
| MOL012533 | quercetin-3-O-rutinoside | C27H30O16 | 610.57 | 6764-99-9 | Not Available | Radix Achyranthis Bidentatae |
| MOL012535 | rubrosterone | C19H26O5 | 334.45 | 19466-41-2 | 12315102 | Radix Achyranthis Bidentatae |
| MOL012536 | Rubschisantherin | C25H30O8 | 458.55 | 102637-03-6 | 73353444 | Radix Achyranthis Bidentatae |
| MOL012538 | stachysterone A | C27H42O6 | 464.71 | 30655-78-8 | Not Available | Radix Achyranthis Bidentatae |
| MOL012539 | stachysterone D | C27H42O6 | 462.69 | 26361-67-1 | 91745162 | Radix Achyranthis Bidentatae |
| MOL012541 | zingibroside r1_qt | Not Available | 456.78 | 80930-74-1 | Not Available | Radix Achyranthis Bidentatae |
| MOL013187 | Cubebin | C20H20O6 | 356.4 | 18423-69-3 | 117443 | Radix Bupleuri |
| MOL013381 | Marmin | C19H24O5 | 332.43 | 14957-38-1 | 6450230 | Fructus Aurantii |
| MOL013382 | Meranzin | C15H16O4 | 260.31 | 23971-42-8 | 1803558 | Fructus Aurantii |
