# Supplementary material for: Natural formulas and the nature of formulas: Exploring potential therapeutic targets based on traditional Chinese herbal formulas
Source: PLoS One. 2017 Feb 9;12(2):e0171628. doi: 10.1371/journal.pone.0171628 (PMC5300118; doi:10.1371/journal.pone.0171628)
Supplement: S2 Table — (DOCX) [file pone.0171628.s002.docx]

Table 2. Chemical compounds in herbal medicines of GXBD

| MOL_ID | Chemical Compounds | M.F. | M.W. | CAS | Pubchem_CID | Herbal Medicines |
| --- | --- | --- | --- | --- | --- | --- |
| MOL001787 | Adenosine | C10H13N5O4 | 267.280 | 30143-02-3 | 60961 | Bulbus Allii Macrostemonis |
| MOL001780 | (2S)-2-azaniumyl-3-(1H-indol-3-yl) propanoate | C11H12N2O2 | 204.250 | 80206-30-0 | 6923516 | Bulbus Allii Macrostemonis |
| MOL000348 | 4-[(Z)-3-hydroxyprop-1-enyl]-2,6-dimethoxyphenol | C11H14O4 | 210.250 | 118-34-3 | 10130521 | Bulbus Allii Macrostemonis |
| MOL007607 | (3R)-2,3,4,9-tetrahydro-1H-$b-carboline-3-carboxylic acid | C12H12N2O2 | 216.260 | Not Available | 6920043 | Bulbus Allii Macrostemonis |
| MOL007606 | (1R,3R)-1-methyl-2,3,4,9-tetrahydro-1H-$b-carboline-3-carboxylic acid | C13H14N2O2 | 230.290 | 40678-46-4 | 6920042 | Bulbus Allii Macrostemonis |
| MOL000098 | Quercetin | C15H10O7 | 302.250 | 73123-10-1 | 5280343 | Bulbus Allii Macrostemonis |
| MOL001789 | Isoliquiritigenin | C15H12O4 | 256.270 | 961-29-5 | 638278 | Bulbus Allii Macrostemonis |
| MOL004328 | Naringenin | C15H12O5 | 272.270 | 153-18-4 | 439246 | Bulbus Allii Macrostemonis |
| MOL007635 | Lunularic acid | C15H14O4 | 258.290 | 23255-59-6 | 161413 | Bulbus Allii Macrostemonis |
| MOL002341 | Hesperetin | C16H14O6 | 302.300 | 520-33-2 | 72281 | Bulbus Allii Macrostemonis |
| MOL005932 | Gaidic acid | C16H30O2 | 254.460 | 25447-95-4 | 5282743 | Bulbus Allii Macrostemonis |
| MOL000332 | N-coumaroyltyramine | C17H17NO3 | 283.350 | 20375-37-5 | 5372945 | Bulbus Allii Macrostemonis |
| MOL000631 | Coumaroyltyramine | C17H17NO3 | 283.350 | 20375-37-5 | 13939145 | Bulbus Allii Macrostemonis |
| MOL000347 | Syrigin | C17H24O9 | 372.410 | 118-34-3 | 5316860 | Bulbus Allii Macrostemonis |
| MOL007631 | Heptadecenoic acid | C17H32O2 | 268.490 | 26265-99-6 | 5282747 | Bulbus Allii Macrostemonis |
| MOL001501 | Daturic acid | C17H34O2 | 270.510 | 67701-03-5 | 10465 | Bulbus Allii Macrostemonis |
| MOL000483 | (Z)-3-(4-hydroxy-3-methoxy-phenyl)-N-[2-(4-hydroxyphenyl)ethyl]acrylamide | C18H19NO4 | 313.380 | 80510-09-4 | 6440659 | Bulbus Allii Macrostemonis |
| MOL007650 | Prostaglandin A1 | C20H32O4 | 336.520 | 14152-28-4 | 5281912 | Bulbus Allii Macrostemonis |
| MOL007651 | Prostaglandin B1 | C20H32O4 | 336.520 | 13345-42-1 | 5280388 | Bulbus Allii Macrostemonis |
| MOL007610 | 21-methyl tricosanoic acid | C24H48O2 | 368.720 | 36332-96-4 | 71367623 | Bulbus Allii Macrostemonis |
| MOL007634 | Laxogenin | C27H42O4 | 430.690 | 1177-71-5 | 10950057 | Bulbus Allii Macrostemonis |
| MOL003894 | Smilagenin | C27H44O3 | 416.710 | 126-18-1 | 91439 | Bulbus Allii Macrostemonis |
| MOL004495 | Tigogenin | C27H44O3 | 416.710 | 77-60-1 | 99516 | Bulbus Allii Macrostemonis |
| MOL004487 | Gitogenin | C27H44O4 | 432.710 | 6811-13-8 | 12304409 | Bulbus Allii Macrostemonis |
| MOL007621 | Dimethyl disulfide | C2H6S2 | 94.220 | 624-92-0 | 12232 | Bulbus Allii Macrostemonis |
| MOL001973 | Sitosteryl acetate | C31H52O2 | 456.830 | 915-05-9 | 5354503 | Bulbus Allii Macrostemonis |
| MOL007622 | Methyldisulfanylethane | C3H8S2 | 108.250 | 20333-39-5 | 123388 | Bulbus Allii Macrostemonis |
| MOL007632 | 2-methyldisulfanylpropane | C4H10S2 | 122.280 | 40136-65-0 | 123494 | Bulbus Allii Macrostemonis |
| MOL007646 | 2,3-dithiahexane | C4H10S2 | 122.280 | 2179-60-4 | 16592 | Bulbus Allii Macrostemonis |
| MOL007624 | Thiole | C4H4S | 84.150 | 25233-34-5 | 8030 | Bulbus Allii Macrostemonis |
| MOL007619 | Methyl allyl sulfide | C4H8S | 88.190 | 10152-76-8 | 66282 | Bulbus Allii Macrostemonis |
| MOL007643 | Methylallyl disulphide | C4H8S2 | 120.260 | 2179-58-0 | 62434 | Bulbus Allii Macrostemonis |
| MOL007644 | Methyldithio-1-propene | C4H8S2 | 120.260 | 5905-47-5 | 5366552 | Bulbus Allii Macrostemonis |
| MOL007627 | Allyl disulfide | C6H10S2 | 146.300 | 2179-57-9 | 16590 | Bulbus Allii Macrostemonis |
| MOL007620 | Allitridin | C6H10S3 | 178.370 | 2050-87-5 | 16315 | Bulbus Allii Macrostemonis |
| MOL007601 | (+)-L-alliin | C6H11NO3S | 177.250 | 209-118-9 | 51380903 | Bulbus Allii Macrostemonis |
| MOL007648 | 4,5-Dithia-1-octene | C6H12S2 | 148.320 | 2179-59-1 | 16591 | Bulbus Allii Macrostemonis |
| MOL007609 | Allyl propyl trisulfide | C6H12S3 | 180.390 | 33922-73-5 | 525330 | Bulbus Allii Macrostemonis |
| MOL007629 | Propyl disulfide | C6H14S2 | 150.340 | 629-19-6 | 12377 | Bulbus Allii Macrostemonis |
| MOL007649 | 2-propyldisulfanylpropane | C6H14S2 | 150.340 | 33672-51-4 | 118529 | Bulbus Allii Macrostemonis |
| MOL007630 | Propyl trisulfide | C6H14S3 | 182.410 | 58973-40-3 | 22383 | Bulbus Allii Macrostemonis |
| MOL007608 | 2,4-dimethylthiophene | C6H8S | 112.210 | 638-00-6 | 34296 | Bulbus Allii Macrostemonis |
| MOL004919 | Hexahydrotoluene | C7H14 | 98.210 | 108-87-2 | 7962 | Bulbus Allii Macrostemonis |
| MOL007615 | (3S)-3-methylhexane | C7H16 | 100.230 | 589-34-4 | 638046 | Bulbus Allii Macrostemonis |
| MOL004967 | 3,3-dimethylpentane | C7H16 | 100.230 | 562-49-2 | 11229 | Bulbus Allii Macrostemonis |
| MOL002198 | Heptan | C7H16 | 100.230 | 142-82-5 | 8900 | Bulbus Allii Macrostemonis |
| MOL000103 | 4-oxoniobenzoate | C7H6O3 | 138.130 | 99-96-7 | 3702506 | Bulbus Allii Macrostemonis |
| MOL000771 | P-coumaric acid | C9H8O3 | 164.170 | 7400-08-0 | 637542 | Bulbus Allii Macrostemonis |
| MOL007626 | Desgalactotigonin_qt | Not Available | 416.710 | Not Available | Not Available | Bulbus Allii Macrostemonis |
| MOL007637 | Macrostemonoside d_qt | Not Available | 400.710 | Not Available | Not Available | Bulbus Allii Macrostemonis |
| MOL007640 | Macrostemonoside e_qt | Not Available | 416.710 | Not Available | Not Available | Bulbus Allii Macrostemonis |
| MOL007642 | Macrostemonoside f_qt | Not Available | 416.710 | Not Available | Not Available | Bulbus Allii Macrostemonis |
| MOL007654 | Stigmasterol-3-o-β-d-glucoside_qt | Not Available | 398.740 | Not Available | Not Available | Bulbus Allii Macrostemonis |
| MOL006240 | Thymidine | C10H14N2O5 | 242.260 | 50-88-4 | 5789 | Bulbus Allii Macrostemonis, Rhizoma Pinelliae |
| MOL001396 | Pentadecylic acid | C15H30O2 | 242.450 | 1002-84-2 | 13849 | Bulbus Allii Macrostemonis, Rhizoma Pinelliae |
| MOL000131 | Linoleic acid | C18H32O2 | 280.500 | 2197-37-7 | 5280450 | Bulbus Allii Macrostemonis, Rhizoma Pinelliae |
| MOL000358 | Beta-sitosterol | C29H50O | 414.790 | 83-46-5 | 222284 | Bulbus Allii Macrostemonis, Rhizoma Pinelliae |
| MOL000357 | Sitogluside | C35H60O6 | 576.950 | 474-58-8 | 5742590 | Bulbus Allii Macrostemonis, Rhizoma Pinelliae |
| MOL000346 | Succinic acid | C4H6O4 | 118.100 | 110-15-6 | 21952380 | Bulbus Allii Macrostemonis, Rhizoma Pinelliae |
| MOL001640 | Decanoic acid | C10H20O2 | 172.300 | 334-48-5 | 2969 | Fructus Trichosanthis |
| MOL007183 | Dibutyl (2R)-2-hydroxybutanedioate | C12H22O5 | 246.340 | Not Available | 10467092 | Fructus Trichosanthis |
| MOL005530 | Hydroxygenkwanin | C16H12O6 | 300.280 | 6980-25-2 | 5318214 | Fructus Trichosanthis |
| MOL002881 | Diosmetin | C16H12O6 | 300.280 | 520-34-3 | 5281612 | Fructus Trichosanthis |
| MOL002083 | Tricin | C17H14O7 | 330.310 | 520-32-1 | 5281702 | Fructus Trichosanthis |
| MOL000879 | Methyl palmitate | C17H34O2 | 270.510 | 112-39-0 | 8181 | Fructus Trichosanthis |
| MOL007181 | Punicic acid | C18H30O2 | 278.480 | 544-72-9 | 5281126 | Fructus Trichosanthis |
| MOL002038 | 9E,12Z-octadecadienoic acid | C18H32O2 | 280.500 | 2420-55-5 | 5282798 | Fructus Trichosanthis |
| MOL001398 | Methyllinolenate | C19H32O2 | 292.510 | 301-00-8 | 5319706 | Fructus Trichosanthis |
| MOL001641 | Methyl linoleate | C19H34O2 | 294.530 | 112-63-0 | 5284421 | Fructus Trichosanthis |
| MOL007179 | Linolenic acid ethyl ester | C20H34O2 | 306.540 | 1191-41-9 | 5367460 | Fructus Trichosanthis |
| MOL001494 | Mandenol | C20H36O2 | 308.560 | 544-35-4 | 5282184 | Fructus Trichosanthis |
| MOL007182 | 5-hydroxy-2-[4-hydroxy-3-[(2S,3R,4S,5S,6R)-3,4,5-trihydroxy-6-(hydroxymethyl)oxan-2-yl] oxyphenyl]-7-methoxychromen-4-one | C22H22O11 | 462.440 | Not Available | 10479460 | Fructus Trichosanthis |
| MOL007174 | 5-hydroxy-2-(3-hydroxy-4-methoxyphenyl)-7-[(2S, 3R,4S,5S,6R)-3,4,5-trihydroxy-6-(hydroxymethyl) oxan-2-yl]oxychromen-4-one | C22H22O11 | 462.440 | Not Available | 11016019 | Fructus Trichosanthis |
| MOL007180 | Glimepiride | C24H34N4O5S | 490.690 | 1406-18-4 | 3476 | Fructus Trichosanthis |
| MOL000663 | Lignoceric acid | C24H48O2 | 368.720 | 557-59-5 | 11197 | Fructus Trichosanthis |
| MOL000662 | Ceric acid | C26H52O2 | 396.780 | 506-46-7 | 10469 | Fructus Trichosanthis |
| MOL000659 | Montanic acid | C28H56O2 | 424.840 | 506-48-9 | 10470 | Fructus Trichosanthis |
| MOL004355 | Spinasterol | C29H48O | 412.770 | 481-18-5 | 5281331 | Fructus Trichosanthis |
| MOL006756 | Schottenol | C29H50O | 414.790 | 521-03-9 | 441837 | Fructus Trichosanthis |
| MOL007171 | 5-dehydrokarounidiol | C30H46O2 | 438.760 | Not Available | 10094697 | Fructus Trichosanthis |
| MOL007186 | Karounidiol | C30H48O2 | 440.780 | 118117-31-0 | 159490 | Fructus Trichosanthis |
| MOL007185 | Isokarounidiol | C30H48O2 | 440.780 | Not Available | 10003399 | Fructus Trichosanthis |
| MOL000602 | Fumaric acid | C4H4O4 | 116.080 | 110-17-8 | 444972 | Fructus Trichosanthis |
| MOL007176 | Alpha-l-arabinose | C5H10O5 | 150.150 | 5328-37-0 | 439731 | Fructus Trichosanthis |
| MOL007173 | Beta-D-arabinopyranose | C5H10O5 | 150.150 | 87-72-9 | 444173 | Fructus Trichosanthis |
| MOL000731 | Alpha-d-xylopyranose | C5H10O5 | 150.150 | 6763-34-4 | 6027 | Fructus Trichosanthis |
| MOL005449 | L-met | C5H11NO2S | 149.240 | 26062-47-5 | 6992087 | Fructus Trichosanthis |
| MOL000061 | Prolinum | C5H9NO2 | 115.150 | 18875-45-1 | 6971047 | Fructus Trichosanthis |
| MOL007184 | [(2R,5S)-5-(hydroxymethyl)oxolan-2-yl] methanol | C6H12O3 | 132.180 | 1883-75-6 | 12886254 | Fructus Trichosanthis |
| MOL000003 | Mannitol | C6H14O6 | 182.200 | 133-43-7 | 6251 | Fructus Trichosanthis |
| MOL007168 | 4-Hydroxynicotinic acid | C6H5NO3 | 139.120 | 72676-96-1 | 69113 | Fructus Trichosanthis |
| MOL000635 | Vanillin | C8H8O3 | 152.160 | 121-33-5 | 1183 | Fructus Trichosanthis |
| MOL007169 | 4-hydroxy-2-methoxybenzoic acid | C8H8O4 | 168.160 | Not Available | 12695575 | Fructus Trichosanthis |
| MOL000041 | (2S)-2-azaniumyl-3-phenylpropanoate | C9H11NO2 | 165.210 | 5297-02-9 | 6925665 | Fructus Trichosanthis |
| MOL003050 | Nonanoic acid | C9H18O2 | 158.270 | 112-05-0 | 8158 | Fructus Trichosanthis |
| MOL007165 | 10α-cucurbita-5,24-diene-3β-ol | Not Available | 426.800 | Not Available | Not Available | Fructus Trichosanthis |
| MOL007172 | 7-oxo-dihydrokaro-unidiol | Not Available | 456.780 | Not Available | Not Available | Fructus Trichosanthis |
| MOL000305 | Lauric acid | C12H24O2 | 200.360 | 8045-27-0 | 3893 | Fructus Trichosanthis, Bulbus Allii Macrostemonis |
| MOL001393 | Myristic acid | C14H28O2 | 228.420 | 45184-05-2 | 11005 | Fructus Trichosanthis, Bulbus Allii Macrostemonis |
| MOL000069 | Palmitic acid | C16H32O2 | 256.480 | 67701-02-4 | 985 | Fructus Trichosanthis, Bulbus Allii Macrostemonis, Rhizoma Pinelliae |
| MOL000675 | Oleic acid | C18H34O2 | 282.520 | 17156-84-2 | 445639 | Fructus Trichosanthis, Bulbus Allii Macrostemonis, Rhizoma Pinelliae |
| MOL000860 | Stearic acid | C18H36O2 | 284.540 | 609343-71-7 | 5281 | Fructus Trichosanthis, Bulbus Allii Macrostemonis, Rhizoma Pinelliae |
| MOL001739 | Zoomaric acid | C16H30O2 | 254.460 | 373-49-9 | 445638 | Fructus Trichosanthisrhizoma Pinelliae |
| MOL000432 | Linolenic acid | C18H32O2 | 278.480 | 60-33-3 | 5280934 | Fructus Trichosanthisrhizoma Pinelliae |
| MOL000971 | Ethylpalmitate | C18H36O2 | 284.540 | 628-97-7 | 12366 | Fructus Trichosanthisrhizoma Pinelliae |
| MOL000458 | Campesterol | C28H48O | 400.760 | 474-62-4 | 173183 | Fructus Trichosanthisrhizoma Pinelliae |
| MOL000050 | 2-azaniumylacetate | C2H5NO2.xCu | 75.080 | 32817-15-5 | 5257127 | Fructus Trichosanthisrhizoma Pinelliae |
| MOL004739 | (2R)-2-azaniumylpropanoate | C3H7NO2 | 89.110 | 56-41-7 | 7311725 | Fructus Trichosanthisrhizoma Pinelliae |
| MOL000065 | Asi | C4H7NO4.1/2Ca | 133.120 | 39162-75-9 | 44367445 | Fructus Trichosanthisrhizoma Pinelliae |
| MOL003971 | Threonin | C4H9NO3 | 119.140 | 7013-32-3 | 6971019 | Fructus Trichosanthisrhizoma Pinelliae |
| MOL000067 | L-valin | C5H11NO2 | 117.170 | 16872-32-5 | 6971018 | Fructus Trichosanthisrhizoma Pinelliae |
| MOL003969 | L-serin | C5H9NO2 | 105.110 | 6898-95-9 | 6857581 | Fructus Trichosanthisrhizoma Pinelliae |
| MOL000052 | Gulutamine | C5H9NO4 | 147.150 | 26717-13-5 | 44272391 | Fructus Trichosanthisrhizoma Pinelliae |
| MOL005448 | Leucinum | C6H12NO2T | 131.200 | 71000-80-1 | 7045798 | Fructus Trichosanthisrhizoma Pinelliae |
| MOL000068 | L-ile | C6H13NO2 | 131.200 | 73-32-5 | 7043901 | Fructus Trichosanthisrhizoma Pinelliae |
| MOL000055 | L-lysin | C6H14N2O2 | 146.220 | 26714-32-9 | 5962 | Fructus Trichosanthisrhizoma Pinelliae |
| MOL000054 | Arginine | C6H14N4O2 | 174.240 | 142-49-4 | 28782 | Fructus Trichosanthisrhizoma Pinelliae |
| MOL000748 | 5-(Hydroxymethyl)furfural | C6H6O3 | 126.120 | 76330-16-0 | 237332 | Fructus Trichosanthisrhizoma Pinelliae |
| MOL000071 | Istidina | C6H9N3O2 | 155.180 | 30641-68-0 | 6971009 | Fructus Trichosanthisrhizoma Pinelliae |
| MOL000114 | Vanillic acid | C8H8O4 | 168.160 | 121-34-6 | 8468 | Fructus Trichosanthisrhizoma Pinelliae |
| MOL000056 | (2S)-2-azaniumyl-3-(4-hydroxyphenyl)propanoate | C9H11NO3 | 181.210 | 140-43-2 | 6942100 | Fructus Trichosanthisrhizoma Pinelliae |
| MOL000389 | Ferulic acid (cis) | C10H10O4 | 194.200 | 1014-83-1 | 1548883 | Rhizoma Pinelliae |
| MOL006967 | Beta-D-Ribofuranoside, xanthine-9 | C10H12N4O6 | 284.260 | 5968-90-1 | 64959 | Rhizoma Pinelliae |
| MOL000475 | Anethole | C10H12O | 148.220 | 12002-40-3 | 637563 | Rhizoma Pinelliae |
| MOL006594 | Ephedrine | C10H15NO | 165.260 | 299-42-3 | 9294 | Rhizoma Pinelliae |
| MOL006932 | L-Pseudoephedrine | C10H15NO | 165.260 | 7009-81-6 | 62946 | Rhizoma Pinelliae |
| MOL000924 | 2-undecanone | C11H22O | 170.330 | 112-12-9 | 8163 | Rhizoma Pinelliae |
| MOL006844 | Norharman | C11H8N2 | 168.210 | 244-63-3 | 64961 | Rhizoma Pinelliae |
| MOL006958 | Cyclo-(val-tyr) | C14H18N2O3 | 262.340 | Not Available | 56929107 | Rhizoma Pinelliae |
| MOL001729 | Crysophanol | C15H10O4 | 254.250 | 481-74-3 | 10208 | Rhizoma Pinelliae |
| MOL002714 | Baicalein | C15H10O5 | 270.250 | 491-67-8 | 5281605 | Rhizoma Pinelliae |
| MOL006956 | Cyclo-(leu-tyr) | C15H20N2O3 | 276.370 | Not Available | 15550385 | Rhizoma Pinelliae |
| MOL000908 | Beta-elemene | C15H24 | 204.390 | 122-78-1 | 6918391 | Rhizoma Pinelliae |
| MOL000875 | Cedrol | C15H24O | 222.410 | 77-53-2 | 65575 | Rhizoma Pinelliae |
| MOL006962 | 2Z-hexadecenoic acid | C16H30O2 | 254.460 | 373-49-9 | 5312417 | Rhizoma Pinelliae |
| MOL002495 | 6-shogaol | C17H24O3 | 276.410 | 555-66-8 | 5281794 | Rhizoma Pinelliae |
| MOL006960 | (5R)-5-hydroxy-1-(4-hydroxy-3-methoxyphenyl) tetradecan-3-one | C17H26O4 | 350.550 | 23513-14-6 | 23786427 | Rhizoma Pinelliae |
| MOL001818 | Methyl palmitelaidate | C17H32O2 | 268.490 | 10030-74-7 | 638303 | Rhizoma Pinelliae |
| MOL006957 | (3S,6S)-3-(benzyl)-6-(4-hydroxybenzyl)piperazine-2,5-quinone | C18H18N2O3 | 310.380 | Not Available | 11438306 | Rhizoma Pinelliae |
| MOL006944 | 8-Octadecenoic acid | C18H34O2 | 282.520 | 2197-55-9 | 5282758 | Rhizoma Pinelliae |
| MOL003870 | Gynesine | C18H34O2 | 137.150 | 112-80-1 | 5571 | Rhizoma Pinelliae |
| MOL000519 | Coniferin | C19H22O4 | 314.410 | 109664-02-0 | 389888 | Rhizoma Pinelliae |
| MOL006931 | Isolariciresinol | C20H24O6 | 360.440 | 548-29-8 | 160521 | Rhizoma Pinelliae |
| MOL006963 | Ethyl linolelaidate | C20H36O2 | 308.560 | 544-35-4 | 5365672 | Rhizoma Pinelliae |
| MOL005030 | Gondoic acid | C20H38O2 | 310.580 | 5561-99-9 | 5282768 | Rhizoma Pinelliae |
| MOL002776 | Baicalin | C21H18O11 | 446.390 | 31564-28-0 | 64982 | Rhizoma Pinelliae |
| MOL002670 | Cavidine | C21H23NO4 | 353.450 | 32728-75-9 | 193148 | Rhizoma Pinelliae |
| MOL006936 | 10,13-eicosadienoic | C21H38O2 | 308.560 | 30223-50-8 | 5365687 | Rhizoma Pinelliae |
| MOL000399 | Docosanoate | C22H44O2 | 340.660 | 16529-65-0 | 8215 | Rhizoma Pinelliae |
| MOL000449 | Stigmasterol | C29H48O | 412.770 | 83-48-7 | 5280794 | Rhizoma Pinelliae |
| MOL001755 | 24-Ethylcholest-4-en-3-one | C29H48O | 412.770 | 67392-96-5 | 5484202 | Rhizoma Pinelliae |
| MOL003578 | Cycloartenol | C30H50O | 426.800 | 469-38-5 | 382580 | Rhizoma Pinelliae |
| MOL000697 | Meso-erythritol | C4H10O4 | 122.140 | 149-32-6 | 222285 | Rhizoma Pinelliae |
| MOL001744 | Uracil | C4H4N2O2 | 112.100 | 66-22-8 | 1174 | Rhizoma Pinelliae |
| MOL006940 | D-2-aminobutyrate | C4H9NO2 | 103.140 | 2623-91-8 | 6971251 | Rhizoma Pinelliae |
| MOL000388 | Gamma-aminobutyric acid | C4H9NO2 | 103.140 | 28805-76-7 | 6992099 | Rhizoma Pinelliae |
| MOL006934 | Pentane-1,5-diol | C5H12O2 | 104.170 | 111-29-5 | 8105 | Rhizoma Pinelliae |
| MOL001831 | Hypoxanthine | C5H4N4O | 136.130 | 25991-08-6 | 790 | Rhizoma Pinelliae |
| MOL001788 | Adenine | C5H5N5 | 135.150 | 73-24-5 | 190 | Rhizoma Pinelliae |
| MOL001757 | Guanine | C5H5N5O | 151.150 | 11006-44-3 | 764 | Rhizoma Pinelliae |
| MOL006953 | Thymine | C5H6N2O2 | 126.130 | 65-71-4 | 1135 | Rhizoma Pinelliae |
| MOL006954 | N-Butyl vinyl ether | C6H12O | 100.180 | 111-34-2 | 8108 | Rhizoma Pinelliae |
| MOL000579 | Hydroquinone | C6H6O2 | 110.120 | 57534-13-1 | 785 | Rhizoma Pinelliae |
| MOL000172 | Furol | C6H9N3O2 | 96.090 | 98-01-1 | 7362 | Rhizoma Pinelliae |
| MOL000708 | Benzaldehyde | C7H6O | 106.130 | 100-52-7 | 240 | Rhizoma Pinelliae |
| MOL001452 | Protocatechualdehyde | C7H6O3 | 138.130 | 139-85-5 | 8768 | Rhizoma Pinelliae |
| MOL000105 | Protocatechuic acid | C7H6O4 | 154.130 | 99-50-3 | 72 | Rhizoma Pinelliae |
| MOL000513 | 3,4,5-trihydroxybenzoic acid | C7H6O5 | 170.130 | 149-91-7 | 370 | Rhizoma Pinelliae |
| MOL006935 | Octylene | C8H16 | 112.240 | 68526-54-5 | 8125 | Rhizoma Pinelliae |
| MOL005125 | P-Anisic acid | C8H8O3 | 152.160 | 100-09-4 | 7478 | Rhizoma Pinelliae |
| MOL006597 | Homogentisic acid | C8H8O4 | 168.160 | 71694-00-3 | 780 | Rhizoma Pinelliae |
| MOL006938 | Deoxyuridine | C9H12N2O5 | 228.230 | 20649-53-0 | 13712 | Rhizoma Pinelliae |
| MOL006952 | Pedatisectine F | C9H14N2O4 | 200.220 | 206757-32-6 | 12285902 | Rhizoma Pinelliae |
| MOL000397 | Cis-p-Coumarate | C9H8O3 | 164.170 | 4501-31-9 | 1549106 | Rhizoma Pinelliae |
| MOL000223 | Caffeic acid | C9H8O4 | 180.170 | 71693-97-5 | 689043 | Rhizoma Pinelliae |
| MOL006951 | Pedatisectine A | Not Available | 242.270 | Not Available | Not Available | Rhizoma Pinelliae |
| MOL006943 | 5,8-epidioxyergosta-6,22-dien-3-ol | Not Available | 428.720 | Not Available | Not Available | Rhizoma Pinelliae |
| MOL006930 | (+)-Isolariciresinol 9-o-β-d-glucopyranoside | Not Available | 522.600 | Not Available | Not Available | Rhizoma Pinelliae |
| MOL006937 | 12,13-epoxy-9-hydroxynonadeca-7,10-dienoic acid | Not Available | 324.510 | Not Available | Not Available | Rhizoma Pinelliae |
