# Supplementary material for: Natural formulas and the nature of formulas: Exploring potential therapeutic targets based on traditional Chinese herbal formulas
Source: PLoS One. 2017 Feb 9;12(2):e0171628. doi: 10.1371/journal.pone.0171628 (PMC5300118; doi:10.1371/journal.pone.0171628)
Supplement: S3 Table — (DOCX) [file pone.0171628.s003.docx]

S3 Table. Mutual chemical compounds of XZD and GXBD

| MOL_ID | Compound Names | CAS | Pubchem_CID |
| --- | --- | --- | --- |
| MOL004967 | 3,3-Dimethylpentane | 562-49-2 | 11229 |
| MOL005449 | L-Met | 26062-47-5 | 6992087 |
| MOL005125 | P-Anisic acid | 100-09-4 | 7478 |
| MOL004919 | Hexahydrotoluene | 108-87-2 | 7962 |
| MOL000348 | 4-[(Z)-3-hydroxyprop-1-enyl]-2,6-dimethoxyphenol | 118-34-3 | 10130521 |
| MOL001729 | Crysophanol | 481-74-3 | 10208 |
| MOL001501 | Daturic acid | 67701-03-5 | 10465 |
| MOL001393 | Myristic Acid | 45184-05-2 | 11005 |
| MOL001744 | Uracil | 66-22-8 | 1174 |
| MOL000635 | Vanillin | 121-33-5 | 1183 |
| MOL000971 | Ethylpalmitate | 628-97-7 | 12366 |
| MOL001396 | Pentadecylic acid | 1002-84-2 | 13849 |
| MOL000389 | Ferulic acid, cis- | 1014-83-1 | 1548883 |
| MOL000397 | cis-p-Coumarate | 4501-31-9 | 1549106 |
| MOL001788 | Adenine | 73-24-5 | 190 |
| MOL000346 | Succinic Acid | 110-15-6 | 21952380 |
| MOL000358 | *β*-sitosterol | 83-46-5 | 222284 |
| MOL000748 | 5-(Hydroxymethyl)furfural | 76330-16-0 | 237332 |
| MOL000708 | Benzaldehyde | 100-52-7 | 240 |
| MOL001640 | Decanoic acid | 334-48-5 | 2969 |
| MOL000513 | 3,4,5-Trihydroxybenzoic acid | 149-91-7 | 370 |
| MOL000103 | 4-Oxoniobenzoate | 99-96-7 | 3702506 |
| MOL000305 | Lauric acid | 8045-27-0 | 3893 |
| MOL004328 | Naringenin | 153-18-4 | 439246 |
| MOL001739 | Zoomaric acid | 373-49-9 | 445638 |
| MOL000675 | Oleic acid | 17156-84-2 | 445639 |
| MOL000098 | Quercetin | 73123-10-1 | 5280343 |
| MOL000131 | Linoleic acid | 2197-37-7 | 5280450 |
| MOL000449 | Stigmasterol | 83-48-7 | 5280794 |
| MOL000432 | Linolenic acid | 60-33-3 | 5280934 |
| MOL000860 | Stearic acid | 609343-71-7 | 5281 |
| MOL004355 | Spinasterol | 481-18-5 | 5281331 |
| MOL002714 | Baicalein | 491-67-8 | 5281605 |
| MOL001494 | Mandenol | 544-35-4 | 5282184 |
| MOL001641 | Methyl linoleate | 112-63-0 | 5284421 |
| MOL000347 | Syrigin | 118-34-3 | 5316860 |
| MOL001398 | Methyllinolenate | 301-00-8 | 5319706 |
| MOL000357 | Sitogluside | 474-58-8 | 5742590 |
| MOL000055 | L-lysin | 26714-32-9 | 5962 |
| MOL001787 | Adenosine | 30143-02-3 | 60961 |
| MOL000003 | Mannitol | 133-43-7 | 6251 |
| MOL000475 | Anethole | 12002-40-3 | 637563 |
| MOL001789 | Isoliquiritigenin | 961-29-5 | 638278 |
| MOL002776 | Baicalin | 31564-28-0 | 64982 |
| MOL000223 | Caffeic acid | 71693-97-5 | 689043 |
| MOL000908 | *β*-elemene | 122-78-1 | 6918391 |
| MOL000067 | L-Valin | 16872-32-5 | 6971018 |
| MOL000388 | *γ*-aminobutyric acid | 28805-76-7 | 6992099 |
| MOL000068 | L-Ile | 73-32-5 | 7043901 |
| MOL000105 | Protocatechuic acid | 99-50-3 | 72 |
| MOL002341 | Hesperetin | 520-33-2 | 72281 |
| MOL000172 | Furol | 98-01-1 | 7362 |
| MOL000579 | Hydroquinone | 57534-13-1 | 785 |
| MOL003050 | Nonanoic acid | 112-05-0 | 8158 |
| MOL000924 | 2-undecanone | 112-12-9 | 8163 |
| MOL000879 | Methyl palmitate | 112-39-0 | 8181 |
| MOL000399 | Docosanoate | 16529-65-0 | 8215 |
| MOL000114 | Vanillic acid | 121-34-6 | 8468 |
| MOL002198 | Heptan | 142-82-5 | 8900 |
| MOL000069 | Palmitic acid | 67701-02-4 | 985 |
