# Supplementary material for: Natural formulas and the nature of formulas: Exploring potential therapeutic targets based on traditional Chinese herbal formulas
Source: PLoS One. 2017 Feb 9;12(2):e0171628. doi: 10.1371/journal.pone.0171628 (PMC5300118; doi:10.1371/journal.pone.0171628)
Supplement: S4 Table — (DOCX) [file pone.0171628.s004.docx]

S4 Table. Target proteins of herbal medicines in XZD

| ID | Proteins | Synonyms | UniProtKB | Herbal Medicines |
| --- | --- | --- | --- | --- |
| **1** | Nitric oxide synthase, inducible | NOS2 | P35228 | Radix Bupleuri, Radix Paeoniae Rubra, Rhizoma Chuanxiong, Radix Angelicae Sinensis, Radix et Rhizoma Glycyrrhizae, Flos Carthami, Radix Platycodonis, Radix Achyranthis Bidentatae, Semen Persicae, Fructus Aurantii, Radix Rehmanniae |
| **2** | Prostaglandin G/H synthase 1 | PTGS1 | P23219 | Radix Bupleuri, Radix Paeoniae Rubra, Rhizoma Chuanxiong, Radix Angelicae Sinensis, Radix et Rhizoma Glycyrrhizae, Flos Carthami, Radix Platycodonis, Radix Achyranthis Bidentatae, Semen Persicae, Fructus Aurantii, Radix Rehmanniae |
| **3** | D(1A) dopamine receptor | DRD1 | P21728 | Radix Bupleuri, Radix Paeoniae Rubra, Rhizoma Chuanxiong, Radix Angelicae Sinensis, Radix et Rhizoma Glycyrrhizae, Flos Carthami, Radix Achyranthis Bidentatae, Semen Persicae, Fructus Aurantii |
| **4** | Insulin receptor | INSR | P06213 | Radix Bupleuri, Radix Et Rhizoma Glycyrrhizae, Flos Carthami, Radix Platycodonis, Radix Achyranthis Bidentatae |
| **5** | Muscarinic acetylcholine receptor M3 | CHRM3 | P20309 | Radix Bupleuri, Radix Paeoniae Rubra, Rhizoma Chuanxiong, Radix Angelicae Sinensis, Radix et Rhizoma Glycyrrhizae, Flos Carthami, Radix Achyranthis Bidentatae, Semen Persicae, Fructus Aurantii |
| **6** | prothrombin | F2 | P00734 | Radix Bupleuri, Radix Paeoniae Rubra, Rhizoma Chuanxiong, Radix Angelicae Sinensis, Radix et Rhizoma Glycyrrhizae, Flos Carthami, Radix Platycodonis, Radix Achyranthis Bidentatae, Semen Persicae, Fructus Aurantii, Radix Rehmanniae |
| **7** | Type-1 angiotensin II receptor | AGTR1 | P50052 | Radix Bupleuri |
| **8** | Nitric oxide synthase, brain | NOS1 | P29475 | Radix Bupleuri, Radix Angelicae Sinensis, Flos Carthami, Radix Platycodonis, Radix Achyranthis Bidentatae |
| **9** | Potassium voltage-gated channel subfamily H member 2 | KCNA4 | Q9H252 | Radix Bupleuri, Radix Paeoniae Rubra, Rhizoma Chuanxiong, Radix Angelicae Sinensis, Radix et Rhizoma Glycyrrhizae, Flos Carthami, Radix Achyranthis Bidentatae, Semen Persicae, Radix Rehmanniae |
| **10** | Muscarinic acetylcholine receptor M1 | CHRM1 | P11229 | Radix Bupleuri, Radix Paeoniae Rubra, Rhizoma Chuanxiong, Radix Angelicae Sinensis, Radix Et Rhizoma Glycyrrhizae, Flos Carthami, Radix Achyranthis Bidentatae, Semen Persicae |
| **12** | Estrogen receptor | ESR1 | P03372 | Radix Bupleuri, Radix Paeoniae Rubra, Rhizoma Chuanxiong, Radix Angelicae Sinensis, Radix et Rhizoma Glycyrrhizae, Flos Carthami, Radix Platycodonis, Radix Achyranthis Bidentatae, Semen Persicae, Fructus Aurantii, Radix Rehmanniae |
| **13** | Androgen receptor | AR | P10275 | Radix Bupleuri, Radix Paeoniae Rubra, Rhizoma Chuanxiong, Radix Angelicae Sinensis, Radix et Rhizoma Glycyrrhizae, Flos Carthami, Radix Platycodonis, Radix Achyranthis Bidentatae, Semen Persicae, Fructus Aurantii, Radix Rehmanniae |
| **14** | Aldehyde dehydrogenase, mitochondrial | ALDH2 | P05091 | Rhizoma Chuanxiong, Flos Carthami, Radix Achyranthis Bidentatae |
| **15** | Succinate semialdehyde dehydrogenase, mitochondrial | ALDH5A1 | P51649 | Radix Angelicae Sinensis, Flos Carthami, Radix Achyranthis Bidentatae |
| **16** | Beta-1 adrenergic receptor | ADRB1 | P08588 | Radix Bupleuri, Radix Paeoniae Rubra, Rhizoma Chuanxiong, Radix Angelicae Sinensis, Radix Et Rhizoma Glycyrrhizae, Flos Carthami, Radix Achyranthis Bidentatae, Semen Persicae, Fructus Aurantii |
| **17** | Sodium channel protein type 10 subunit alpha | SCN3A | Q9Y5Y9 | Rhizoma Chuanxiong, Radix Angelicae Sinensis, |
| **18** | cGMP-specific 3',5'-cyclic phosphodiesterase | PDE5A | O76074 | Radix Achyranthis Bidentatae |
| **19** | Sodium channel protein type 5 subunit alpha | SCN5A | Q14524 | Radix Bupleuri,Radix Paeoniae Rubra, Rhizoma Chuanxiong, Radix Angelicae Sinensis, Radix et Rhizoma Glycyrrhizae, Flos Carthami, Radix Achyranthis Bidentatae, Semen Persicae, Fructus Aurantii, Radix Rehmanniae |
| **20** | Retinoic acid receptor beta | RARB | P10826 | Radix Achyranthis Bidentatae |
| **21** | Plasminogen | PLG | P00747 | Radix Bupleuri, Radix Paeoniae Rubra, Rhizoma Chuanxiong, Flos Carthami, |
| **22** | High-affinity cationic amino acid transporter-1 | SLC7A1 | P30825 | Flos Carthami |
| **23** | Peroxisome proliferator-activated receptor gamma | PPARG | P37231 | Radix Bupleuri, Radix Paeoniae Rubra, Rhizoma Chuanxiong, Radix Angelicae Sinensis, Radix et Rhizoma Glycyrrhizae, Flos Carthami, Radix Platycodonis, Radix Achyranthis Bidentatae, Semen Persicae, Fructus Aurantii, Radix Rehmanniae |
| **24** | Coagulation factor X | F10 | P00742 | Radix Bupleuri,Radix Paeoniae Rubra, Rhizoma Chuanxiong, Radix Angelicae Sinensis, Radix et Rhizoma Glycyrrhizae, Flos Carthami, Radix Achyranthis Bidentatae, Semen Persicae, Fructus Aurantii, Radix Rehmanniae |
| **25** | Ribosyldihydronicotinamide dehydrogenase [quinone] | NQO2 | P16083 | Radix Bupleuri, Radix Angelicae Sinensis, Radix et Rhizoma Glycyrrhizae, Flos Carthami, Semen Persicae, Fructus Aurantii |
| **26** | Lipoprotein lipase | LPL | P06858 | Radix Bupleuri, Rhizoma Chuanxiong, Radix Angelicae Sinensis, Flos Carthami, Radix Platycodonis, Radix Achyranthis Bidentatae |
| **27** | Apoptosis regulator Bcl-2 | BCL2 | P10415 | Radix Bupleuri, Radix Paeoniae Rubra, Rhizoma Chuanxiong, Radix Angelicae Sinensis, Radix et Rhizoma Glycyrrhizae, Flos Carthami, Radix Platycodonis, Radix Achyranthis Bidentatae, Radix Rehmanniae |
| **28** | Muscarinic acetylcholine receptor M5 | CHRM5 | p08912 | Radix Bupleuri, Radix Et Rhizoma Glycyrrhizae, Radix Rehmanniae |
| **29** | Arachidonate 5-lipoxygenase | ALOX5 | P09917 | Radix Bupleuri, Radix Paeoniae Rubra, Radix Et Rhizoma Glycyrrhizae, Flos Carthami, Radix Achyranthis Bidentatae |
| **30** | Sodium-and chloride-dependent GABA transporter 1 | SLC6A1 | P30531 | Radix Rehmanniae |
| **31** | Purine nucleoside phosphorylase | PNP | P00491 | Rhizoma Chuanxiong, Radix Angelicae Sinensis, Flos Carthami, Radix Achyranthis Bidentatae |
| **32** | 4-aminobutyrate aminotransferase, mitochondrial | ABAT | P80404 | Rhizoma Chuanxiong, Radix Angelicae Sinensis, Radix Et Rhizoma Glycyrrhizae, Flos Carthami, Radix Platycodonis, Radix Achyranthis Bidentatae, Semen Persicae, Fructus Aurantii |
| **33** | Prostaglandin G/H synthase 2 | PTGS2 | P35354 | Radix Bupleuri, Radix Paeoniae Rubra, Rhizoma Chuanxiong, Radix Angelicae Sinensis, Radix Et Rhizoma Glycyrrhizae, Flos Carthami, Radix Platycodonis, Radix Achyranthis Bidentatae, Semen Persicae, Fructus Aurantii, Radix Rehmanniae |
| **34** | Nitric-oxide synthase, endothelial | NOS3 | P29474 | Radix Bupleuri, Radix Paeoniae Rubra, Rhizoma Chuanxiong, Radix Angelicae Sinensis, Radix Et Rhizoma Glycyrrhizae, Flos Carthami, Radix Platycodonis, Radix Achyranthis Bidentatae, Semen Persicae, Fructus Aurantii, Radix Rehmanniae |
| **35** | Carbonic anhydrase I | CA1 | P00915 | Radix Bupleuri, Radix Angelicae Sinensis, Radix Et Rhizoma Glycyrrhizae, Radix Rehmanniae |
| **36** | Renin | REN | P00797 | Radix Bupleuri, Rhizoma Chuanxiong, Radix et Rhizoma Glycyrrhizae, Flos Carthami, Radix Platycodonis, Radix Achyranthis Bidentatae, Radix Rehmanniae |
| **37** | Alpha-2A adrenergic receptor | ADRA2A | P08913 | Radix Bupleuri, Radix Paeoniae Rubra, Rhizoma Chuanxiong, Radix Angelicae Sinensis, Radix et Rhizoma Glycyrrhizae, Flos Carthami, Radix Achyranthis Bidentatae, Semen Persicae, Fructus Aurantii |
| **38** | Serine hydroxymethyltransferase, mitochondrial | SHMT2 | P34897 | Flos Carthami |
| **39** | DNA-dependent protein kinase catalytic subunit | PRKDC | P78527 | Flos Carthami, Radix Achyranthis Bidentatae |
| **40** | 5-hydroxytryptamine receptor 3A | HTR3A | P46098 | Radix Bupleuri, Radix Paeoniae Rubra, Rhizoma Chuanxiong, Radix Angelicae Sinensis, Radix et Rhizoma Glycyrrhizae, Flos Carthami, Radix Achyranthis Bidentatae, Radix Rehmanniae |
| **41** | Carbonic anhydrase II | CA2 | P00918 | Radix Bupleuri, Radix Paeoniae Rubra, Rhizoma Chuanxiong, Radix Angelicae Sinensis, Radix et Rhizoma Glycyrrhizae, Flos Carthami, Radix Platycodonis, Radix Achyranthis Bidentatae, Semen Persicae, Fructus Aurantii, Radix Rehmanniae |
| **42** | Coagulation factor VII | F7 | P08709 | Radix Bupleuri, Rhizoma Chuanxiong, Radix et Rhizoma Glycyrrhizae, Flos Carthami, Radix Achyranthis Bidentatae, Semen Persicae, Radix Rehmanniae |
| **44** | Alpha-2C adrenergic receptor | ADRA2C | P18825 | Radix Bupleuri, Radix Paeoniae Rubra, Rhizoma Chuanxiong, Radix Angelicae Sinensis, Radix et Rhizoma Glycyrrhizae, Flos Carthami, Semen Persicae, Fructus Aurantii |
| **47** | Vascular endothelial growth factor receptor 2 | KDR | P35968 | Radix Bupleuri, Rhizoma Chuanxiong, Radix Et Rhizoma Glycyrrhizae, Flos Carthami, Radix Achyranthis Bidentatae, Semen Persicae, Radix Rehmanniae |
| **48** | Vitamin K-dependent protein C | PROC | P04070 | Flos Carthami, Semen Persicae |
| **49** | Gamma-aminobutyric-acid receptor alpha-2 subunit | GABRA2 | P47869 | Radix Bupleuri, Radix Paeoniae Rubra, Rhizoma Chuanxiong, Radix Angelicae Sinensis, Radix et Rhizoma Glycyrrhizae, Flos Carthami, Radix Platycodonis, Radix Achyranthis Bidentatae, Radix Rehmanniae |
| **50** | Substance-P receptor | TACR1 | P25103 | Radix Bupleuri |
| **51** | Kynureninase | KYNU | Q16719 | Radix Achyranthis Bidentatae |
| **52** | Ornithine decarboxylase | ODC1 | P11926 | Radix Bupleuri, Radix et Rhizoma Glycyrrhizae, Flos Carthami, Radix Achyranthis Bidentatae |
| **53** | Muscarinic acetylcholine receptor M4 | CHRM4 | P08173 | Radix Bupleuri, Radix Paeoniae Rubra, Rhizoma Chuanxiong, Radix Angelicae Sinensis, Radix et Rhizoma Glycyrrhizae, Flos Carthami, Radix Achyranthis Bidentatae, Semen Persicae, Radix Rehmanniae |
| **54** | Retinoic acid receptor RXR-alpha | RXRA | P19793 | Radix Bupleuri, Radix Paeoniae Rubra, Rhizoma Chuanxiong, Radix Angelicae Sinensis, Radix Et Rhizoma Glycyrrhizae, Flos Carthami, Radix Platycodonis, Radix Achyranthis Bidentatae, Semen Persicae, Fructus Aurantii, Radix Rehmanniae |
| **56** | Delta-type opioid receptor | OPRD1 | P41143 | Radix Et Rhizoma Glycyrrhizae |
| **57** | Ornithine aminotransferase, mitochondrial | OAT | P04181 | Radix Angelicae Sinensis, Flos Carthami, Radix Achyranthis Bidentatae |
| **58** | Acetylcholinesterase | ACHE | P22303 | Radix Bupleuri, Radix Paeoniae Rubra, Rhizoma Chuanxiong, Radix Angelicae Sinensis, Radix et Rhizoma Glycyrrhizae, Flos Carthami, Radix Platycodonis, Radix Achyranthis Bidentatae, Radix Rehmanniae |
| **59** | Glycine receptor subunit alpha-1 | GLRA1 | P23415 | Radix Angelicae Sinensis, Flos Carthami, Radix Platycodonis, Radix Achyranthis Bidentatae, Semen Persicae, Fructus Aurantii |
| **60** | cGMP-inhibited 3',5'-cyclic phosphodiesterase A | PDE3A | Q14432 | Radix Bupleuri, Radix Paeoniae Rubra, Rhizoma Chuanxiong, Radix Angelicae Sinensis, Radix et Rhizoma Glycyrrhizae, Flos Carthami, Radix Platycodonis, Radix Achyranthis Bidentatae, Semen Persicae, Fructus Aurantii, Radix Rehmanniae |
| **61** | 5-hydroxytryptamine receptor 2A | HTR2A | P28223 | Radix Bupleuri, Radix Paeoniae Rubra, Rhizoma Chuanxiong, Radix Angelicae Sinensis, Radix et Rhizoma Glycyrrhizae, Flos Carthami, Radix Achyranthis Bidentatae, Semen Persicae, Fructus Aurantii |
| **62** | Gamma-aminobutyric-acid receptor alpha-5 subunit | GABRA5 | P31644 | Radix Bupleuri, Radix Paeoniae Rubra, Rhizoma Chuanxiong, Radix Angelicae Sinensis, Radix et Rhizoma Glycyrrhizae, Flos Carthami, Radix Achyranthis Bidentatae, Radix Rehmanniae |
| **63** | Sodium-dependent noradrenaline transporter | SLC6A2 | P23975 | Radix Bupleuri, Radix Paeoniae Rubra, Rhizoma Chuanxiong, Radix Angelicae Sinensis, Radix et Rhizoma Glycyrrhizae, Flos Carthami, Radix Achyranthis Bidentatae, Fructus Aurantii |
| **64** | Low-density lipoprotein receptor | LDLR | P01130 | Radix Et Rhizoma Glycyrrhizae |
| **65** | Alpha-1A adrenergic receptor | ADRA1A | P35348 | Radix Bupleuri,Radix Paeoniae Rubra, Rhizoma Chuanxiong, Radix Angelicae Sinensis, Radix et Rhizoma Glycyrrhizae, Flos Carthami, Radix Achyranthis Bidentatae, Semen Persicae, Fructus Aurantii, Radix Rehmanniae |
| **66** | Thyroid peroxidase | TPO | P07202 | Radix Paeoniae Rubra, Flos Carthami |
| **67** | Gamma-aminobutyric-acid receptor alpha-3 subunit | GABRA3 | P34903 | Radix Bupleuri,Radix Paeoniae Rubra, Rhizoma Chuanxiong, Radix Angelicae Sinensis, Radix et Rhizoma Glycyrrhizae, Flos Carthami, Radix Achyranthis Bidentatae, Semen Persicae, Fructus Aurantii, Radix Rehmanniae |
| **68** | Aspartate aminotransferase, cytoplasmic | GOT1 | P17174 | Radix Angelicae Sinensis, Radix Et Rhizoma Glycyrrhizae, Flos Carthami, Radix Platycodonis, Radix Achyranthis Bidentatae, Semen Persicae, Fructus Aurantii |
| **69** | 5-hydroxytryptamine receptor 2C | HTR2C | P28335 | Radix Bupleuri, Radix Rehmanniae |
| **70** | Carbonic anhydrase IV | CA4 | P22748 | Radix Bupleuri |
| **71** | Progesterone receptor | PGR | P06401 | Radix Bupleuri, Radix Paeoniae Rubra, Rhizoma Chuanxiong, Radix Angelicae Sinensis, Radix et Rhizoma Glycyrrhizae, Flos Carthami, Radix Platycodonis, Radix Achyranthis Bidentatae, Semen Persicae, Fructus Aurantii, Radix Rehmanniae |
| **72** | Muscarinic acetylcholine receptor M2 | CHRM2 | P08172 | Radix Bupleuri, Radix Paeoniae Rubra, Rhizoma Chuanxiong, Radix Angelicae Sinensis, Radix et Rhizoma Glycyrrhizae, Flos Carthami, Radix Achyranthis Bidentatae, Fructus Aurantii, Radix Rehmanniae |
| **73** | Alpha-2B adrenergic receptor | ADRA2B | P18089 | Radix Bupleuri, Radix Paeoniae Rubra, Rhizoma Chuanxiong, Radix Angelicae Sinensis, Flos Carthami, Semen Persicae, Fructus Aurantii |
| **74** | Alpha-1B adrenergic receptor | ADRA1B | P35368 | Radix Bupleuri, Radix Paeoniae Rubra, Rhizoma Chuanxiong, Radix Angelicae Sinensis, Radix et Rhizoma Glycyrrhizae, Flos Carthami, Radix Achyranthis Bidentatae, Fructus Aurantii |
| **75** | Aldo-keto reductase family 1 member C3 | AKR1C3 | P42330 | Radix Bupleuri, Radix Et Rhizoma Glycyrrhizae, Flos Carthami, Radix Achyranthis Bidentatae |
| **76** | Tyrosine 3-monooxygenase | TH | P07101 | Flos Carthami, Semen Persicae |
| **77** | mRNA of Protein-tyrosine phosphatase, non-receptor type 1 | PTPN1 | P18031 | Radix Bupleuri, Radix Paeoniae Rubra, Rhizoma Chuanxiong, Radix Angelicae Sinensis, Radix et Rhizoma Glycyrrhizae, Flos Carthami, Radix Platycodonis, Radix Achyranthis Bidentatae, Semen Persicae, Radix Rehmanniae |
| **78** | Acetyl-CoA carboxylase 1 | ACACA | Q13085 | Radix Bupleuri, Radix et Rhizoma Glycyrrhizae, Flos Carthami, Radix Achyranthis Bidentatae |
| **79** | Kappa-type opioid receptor | OPRK1 | P41145 | Radix Achyranthis Bidentatae |
| **80** | Glutamate receptor 1 | GRIA1 | P42261 | Radix Achyranthis Bidentatae |
| **81** | 72 kDa type IV collagenase | MMP2 | P08253 | Radix Bupleuri, Radix Paeoniae Rubra, Radix et Rhizoma Glycyrrhizae, Flos Carthami, Radix Platycodonis, Radix Achyranthis Bidentatae, Semen Persicae, Radix Rehmanniae |
| **82** | Sodium-dependent dopamine transporter | SLC6A3 | Q01959 | Radix Bupleuri,Radix Paeoniae Rubra, Rhizoma Chuanxiong, Radix Angelicae Sinensis, Radix et Rhizoma Glycyrrhizae, Flos Carthami, Radix Achyranthis Bidentatae, Semen Persicae, Fructus Aurantii, Radix Rehmanniae |
| **83** | Glutathione reductase, mitochondrial | GSR | P00390 | Radix Bupleuri, Radix Et Rhizoma Glycyrrhizae |
| **84** | Cytosolic phospholipase A2 | PLA2G4A | P47712 | Fructus Aurantii |
| **85** | Mineralocorticoid receptor | NR3C2 | P08235 | Radix Bupleuri, Radix Paeoniae Rubra, Rhizoma Chuanxiong, Radix Angelicae Sinensis, Radix et Rhizoma Glycyrrhizae, Flos Carthami, Radix Platycodonis, Radix Achyranthis Bidentatae, Semen Persicae, Fructus Aurantii, Radix Rehmanniae |
| **86** | Beta-2 adrenergic receptor | ADRB2 | P07550 | Radix Bupleuri, Radix Paeoniae Rubra, Rhizoma Chuanxiong, Radix Angelicae Sinensis, Radix et Rhizoma Glycyrrhizae, Flos Carthami, Radix Platycodonis, Radix Achyranthis Bidentatae, Semen Persicae, Fructus Aurantii, Radix Rehmanniae |
| **87** | Tumor necrosis factor | TNF | P01375 | Radix Bupleuri, Radix Paeoniae Rubra, Rhizoma Chuanxiong, Radix Angelicae Sinensis, Radix et Rhizoma Glycyrrhizae, Flos Carthami, Radix Platycodonis, Radix Achyranthis Bidentatae, Semen Persicae, Radix Rehmanniae |
| **88** | Retinoic acid receptor RXR-gamma | RXRG | P48443 | Flos Carthami, Semen Persicae, Fructus Aurantii |
| **89** | Alpha-1D adrenergic receptor | ADRA1D | P25100 | Radix Bupleuri, Radix Paeoniae Rubra, Rhizoma Chuanxiong, Radix Angelicae Sinensis, Radix et Rhizoma Glycyrrhizae, Flos Carthami, Radix Achyranthis Bidentatae, Radix Rehmanniae |
| **90** | Branched-chain-amino-acid aminotransferase, mitochondrial | BCAT2 | O15382 | Radix Angelicae Sinensis, Flos Carthami, Radix Platycodonis, Radix Achyranthis Bidentatae |
| **91** | Neuronal acetylcholine receptor subunit alpha-2 | CHRNA2 | Q15822 | Radix Bupleuri, Radix Paeoniae Rubra, Rhizoma Chuanxiong, Radix Angelicae Sinensis, Radix et Rhizoma Glycyrrhizae, Flos Carthami, Radix Achyranthis Bidentatae, Semen Persicae, Fructus Aurantii |
| **92** | DNA topoisomerase 2-alpha | TOP2A | P11388 | Radix Bupleuri, Radix Paeoniae Rubra, Rhizoma Chuanxiong, Radix Angelicae Sinensis, Radix et Rhizoma Glycyrrhizae, Flos Carthami, Radix Achyranthis Bidentatae, Semen Persicae, Radix Rehmanniae |
| **93** | Aldose reductase | AKR1B1 | P15121 | Radix Bupleuri, Radix Paeoniae Rubra, Rhizoma Chuanxiong, Radix Angelicae Sinensis, Radix et Rhizoma Glycyrrhizae, Flos Carthami, Radix Platycodonis, Radix Achyranthis Bidentatae, Semen Persicae |
| **94** | Sodium-dependent serotonin transporter | SLC6A4 | P31645 | Radix Bupleuri, Radix Paeoniae Rubra, Rhizoma Chuanxiong, Radix Angelicae Sinensis, Radix et Rhizoma Glycyrrhizae, Flos Carthami, Radix Achyranthis Bidentatae, Semen Persicae, Fructus Aurantii |
| **95** | D(2) dopamine receptor | DRD2 | P14416 | Rhizoma Chuanxiong, Radix Angelicae Sinensis |
| **97** | Thromboxane A2 receptor | TBXA2R | P21731 | Radix Bupleuri, Radix et Rhizoma Glycyrrhizae, Flos Carthami, Radix Achyranthis Bidentatae |
| **98** | Glutamate receptor ionotropic, NMDA 2A | GRIN2A | Q12879 | Radix Bupleuri, Radix Achyranthis Bidentatae |
| **99** | Epidermal growth factor receptor | EGFR | P00533 | Radix Bupleuri, Radix et Rhizoma Glycyrrhizae, Flos Carthami, Radix Platycodonis, Radix Achyranthis Bidentatae |
| **100** | Mu-type opioid receptor | OPRM1 | P35372 | Radix Bupleuri, Radix Paeoniae Rubra, Radix Angelicae Sinensis, Radix et Rhizoma Glycyrrhizae, Flos Carthami, Radix Achyranthis Bidentatae, Radix Rehmanniae |
| **101** | Multidrug resistance-associated protein 1 | ABCC1 | P33527 | Radix et Rhizoma Glycyrrhizae |
| **102** | Estrogen receptor beta | ESR2 | Q92731 | Radix Bupleuri, Radix Paeoniae Rubra, Rhizoma Chuanxiong, Radix Angelicae Sinensis, Radix et Rhizoma Glycyrrhizae, Flos Carthami, Radix Platycodonis, Radix Achyranthis Bidentatae, Semen Persicae, Fructus Aurantii, Radix Rehmanniae |
| **103** | Glucocorticoid receptor | NR3C1 | P04150 | Radix Bupleuri, Radix Paeoniae Rubra, Rhizoma Chuanxiong, Radix Angelicae Sinensis, Radix et Rhizoma Glycyrrhizae, Flos Carthami, Radix Platycodonis, Radix Achyranthis Bidentatae, Semen Persicae, Fructus Aurantii, Radix Rehmanniae |
| **104** | Gamma-aminobutyric acid receptor subunit alpha-1 | GABRA1 | P14867 | Radix Bupleuri, Radix Paeoniae Rubra, Rhizoma Chuanxiong, Radix Angelicae Sinensis, Radix et Rhizoma Glycyrrhizae, Flos Carthami, Radix Platycodonis, Radix Achyranthis Bidentatae, Fructus Aurantii, Radix Rehmanniae |
| **105** | Maltase-glucoamylase, intestinal | MGAM | O43451 | Radix Bupleuri, Radix Et Rhizoma Glycyrrhizae, Flos Carthami, Radix Achyranthis Bidentatae |
| **106** | Proto-oncogene tyrosine-protein kinase SRC | SRC | P12931 | Radix Angelicae Sinensis, Flos Carthami, Radix Achyranthis Bidentatae, Semen Persicae, Fructus Aurantii |
| **107** | T-lymphocyte activation antigen CD86 | CD86 | P42081 | Radix Bupleuri, Flos Carthami |
| **108** | T-lymphocyte activation antigen CD80 | CD80 | P33681 | Radix Bupleuri, Flos Carthami |
| **109** | Dipeptidyl peptidase 4 | DPP4 | P27487 | Radix Bupleuri, Radix Paeoniae Rubra, Rhizoma Chuanxiong, Radix Angelicae Sinensis, Radix et Rhizoma Glycyrrhizae, Flos Carthami, Radix Platycodonis, Radix Achyranthis Bidentatae, Semen Persicae, Fructus Aurantii, Radix Rehmanniae |
| **110** | Voltage-dependent T-type calcium channel subunit alpha-1H | CACNA1H | O95180 | Radix Bupleuri |
| **111** | Urokinase-type plasminogen activator | PLAU | P00749 | Radix Bupleuri, Radix Paeoniae Rubra, Rhizoma Chuanxiong, Radix Angelicae Sinensis, Radix et Rhizoma Glycyrrhizae, Flos Carthami, Radix Achyranthis Bidentatae, Semen Persicae |
| **112** | Hepatocyte growth factor receptor | MET | P08581 | Flos Carthami, Radix Platycodonis |
| **113** | Glycogen phosphorylase, muscle form | PYGM | P11217 | Radix Bupleuri, Radix Et Rhizoma Glycyrrhizae, Flos Carthami, |
| **114** | Interleukin-6 | IL6 | P05231 | Radix Bupleuri, Radix Paeoniae Rubra, Rhizoma Chuanxiong, Radix et Rhizoma Glycyrrhizae, Flos Carthami, Radix Platycodonis, Radix Achyranthis Bidentatae, Semen Persicae, Radix Rehmanniae |
| **115** | Interstitial collagenase | MMP1 | P03956 | Radix Bupleuri, Radix et Rhizoma Glycyrrhizae, Flos Carthami, Radix Platycodonis, Radix Achyranthis Bidentatae, Semen Persicae, Radix Rehmanniae |
| **116** | Mitogen-activated protein kinase 1 | MAPK1 | P28482 | Radix Bupleuri, Radix Paeoniae Rubra, Rhizoma Chuanxiong, Radix Angelicae Sinensis, Radix et Rhizoma Glycyrrhizae, Flos Carthami, Radix Platycodonis, Radix Achyranthis Bidentatae |
| **117** | Adenosine receptor A2a | ADORA2A | P29274 | Rhizoma Chuanxiong, Flos Carthami |
| **118** | Serum paraoxonase/arylesterase 1 | PON1 | P27169 | Radix Bupleuri, Radix Paeoniae Rubra, Rhizoma Chuanxiong, Radix Angelicae Sinensis, Radix et Rhizoma Glycyrrhizae, Flos Carthami, Radix Achyranthis Bidentatae |
| **119** | Cathepsin B | CTSB | P07858 | Radix et Rhizoma Glycyrrhizae, Radix Achyranthis Bidentatae, Semen Persicae |
| **121** | Cathepsin D | CTSD | P07339 | Radix Bupleuri, Radix Paeoniae Rubra, Rhizoma Chuanxiong, Radix Angelicae Sinensis, Radix et Rhizoma Glycyrrhizae, Flos Carthami, Radix Platycodonis, Radix Achyranthis Bidentatae, Semen Persicae, Fructus Aurantii |
| **122** | Interferon gamma | IFNG | P01579 | Radix Bupleuri, Radix et Rhizoma Glycyrrhizae, Flos Carthami, Radix Platycodonis, Radix Achyranthis Bidentatae |
| **123** | Fatty acid synthase | FASN | P49327 | Radix Bupleuri, Radix Paeoniae Rubra, Radix et Rhizoma Glycyrrhizae, Flos Carthami, Radix Platycodonis, Semen Persicae, Radix Rehmanniae |
| **124** | catenin Beta-1 | CTNNB1 | P35222 | Radix Achyranthis Bidentatae |
| **125** | Beta-secretase 1 | BACE1 | P56817 | Radix Et Rhizoma Glycyrrhizae |
| **126** | Lactotransferrin | LTF | P02788 | Radix Bupleuri |
| **127** | Peroxisome proliferator-activated receptor delta | PPARD | Q03181 | Radix Bupleuri, Radix et Rhizoma Glycyrrhizae |
| **128** | Cathepsin G | CTSG | P08311 | Radix Achyranthis Bidentatae |
| **130** | Fibroblast growth factor 2 | FGF2 | P09038 | Rhizoma Chuanxiong, Radix et Rhizoma Glycyrrhizae, Semen Persicae |
| **131** | Mitogen-activated protein kinase 14 | MAPK14 | Q16539 | Radix Bupleuri, Radix Paeoniae Rubra, Rhizoma Chuanxiong, Radix Angelicae Sinensis, Radix et Rhizoma Glycyrrhizae, Flos Carthami, Radix Platycodonis, Radix Achyranthis Bidentatae, Semen Persicae, Fructus Aurantii, Radix Rehmanniae |
| **132** | Transient receptor potential cation channel subfamily V member 1 | TRPV1 | Q8NER1 | Radix Bupleuri, Radix Paeoniae Rubra, Rhizoma Chuanxiong, Flos Carthami |
| **133** | Multidrug resistance protein 1 | ABCB1 | P08183 | Radix Bupleuri |
| **134** | Chymase | CMA1 | P23946 | Radix Achyranthis Bidentatae |
| **135** | Transcription factor AP-1 | JUN | P05412 | Radix Bupleuri, Radix Paeoniae Rubra, Rhizoma Chuanxiong, Radix Angelicae Sinensis, Radix et Rhizoma Glycyrrhizae, Flos Carthami, Radix Platycodonis, Radix Achyranthis Bidentatae, Semen Persicae, Radix Rehmanniae |
| **136** | Integrin beta-2 | ITGB2 | P05107 | Radix Bupleuri, Radix et Rhizoma Glycyrrhizae, Flos Carthami, Radix Achyranthis Bidentatae |
| **137** | C-C motif chemokine 2 | CCL2 | P13500 | Radix Bupleuri, Radix et Rhizoma Glycyrrhizae, Flos Carthami, Radix Achyranthis Bidentatae, Semen Persicae, Radix Rehmanniae |
| **138** | Interleukin-1 beta | IL1B | P01584 | Radix Bupleuri, Rhizoma Chuanxiong, Radix et Rhizoma Glycyrrhizae, Flos Carthami, Radix Achyranthis Bidentatae, Semen Persicae |
| **139** | Mitogen-activated protein kinase 3 | MAPK3 | P27361 | Radix Bupleuri, Radix et Rhizoma Glycyrrhizae |
| **140** | Glycogen synthase kinase-3 beta | GSK3B | P49841 | Radix Bupleuri, Radix Paeoniae Rubra, Rhizoma Chuanxiong, Radix Angelicae Sinensis, Radix et Rhizoma Glycyrrhizae, Flos Carthami, Radix Platycodonis, Radix Achyranthis Bidentatae, Semen Persicae, Radix Rehmanniae |
| **141** | E-selectin | SELE | P16581 | Radix Bupleuri, Radix et Rhizoma Glycyrrhizae, Flos Carthami, Radix Achyranthis Bidentatae, Semen Persicae |
| **142** | Myeloperoxidase | MPO | P05164 | Radix Bupleuri, Radix Paeoniae Rubra, Rhizoma Chuanxiong, Radix et Rhizoma Glycyrrhizae, Flos Carthami, Radix Achyranthis Bidentatae |
| **143** | Cell division control protein 2 homolog | CDK1 | p06493 | Radix Bupleuri, Radix Paeoniae Rubra, Rhizoma Chuanxiong, Radix Et Rhizoma Glycyrrhizae, Flos Carthami, Radix Achyranthis Bidentatae |
| **144** | Tissue-type plasminogen activator | PLAT | P00750 | Radix Bupleuri, Radix Et Rhizoma Glycyrrhizae, Flos Carthami, Radix Achyranthis Bidentatae |
| **145** | Gap junction alpha-1 protein | GJA1 | P17302 | Radix Bupleuri, Radix et Rhizoma Glycyrrhizae, Flos Carthami, Radix Achyranthis Bidentatae |
| **146** | Mitogen-activated protein kinase 10 | MAPK10 | P53779 | Radix Bupleuri, Rhizoma Chuanxiong, Radix et Rhizoma Glycyrrhizae, Radix Achyranthis Bidentatae, Radix Rehmanniae |
| **147** | Vascular cell adhesion protein 1 | VCAM1 | P19320 | Radix Bupleuri, Radix et Rhizoma Glycyrrhizae, Flos Carthami, Radix Achyranthis Bidentatae |
| **148** | Stromelysin-1 | MMP3 | P08254 | Radix Bupleuri, Radix et Rhizoma Glycyrrhizae, Flos Carthami, Radix Achyranthis Bidentatae, Semen Persicae |
| **149** | Heat shock protein HSP 90-alpha | HSP90AA1 | P07900 | Radix Bupleuri, Radix Paeoniae Rubra, Rhizoma Chuanxiong, Radix Angelicae Sinensis, Radix et Rhizoma Glycyrrhizae, Flos Carthami, Radix Platycodonis, Radix Achyranthis Bidentatae, Semen Persicae, Fructus Aurantii, Radix Rehmanniae |
| **150** | Thrombomodulin | THBD | P07204 | Radix Bupleuri, Radix et Rhizoma Glycyrrhizae, Flos Carthami, Radix Achyranthis Bidentatae |
| **152** | P-selectin | SELP | P16109 | Radix Bupleuri, Rhizoma Chuanxiong, Flos Carthami, Radix Achyranthis Bidentatae |
| **153** | Tissue factor | F3 | P13726 | Radix Bupleuri, Rhizoma Chuanxiong, Radix et Rhizoma Glycyrrhizae, Flos Carthami, Radix Achyranthis Bidentatae, Radix Rehmanniae |
| **154** | Neutrophil collagenase | MMP8 | P22894 | Rhizoma Chuanxiong, Radix Angelicae Sinensis, Flos Carthami |
| **155** | NAD(P)H dehydrogenase [quinone] 1 | NQO1 | P15559 | Radix Bupleuri, Radix et Rhizoma Glycyrrhizae, Flos Carthami, Radix Achyranthis Bidentatae |
| **156** | Macrophage metalloelastase | MMP12 | P39900 | Flos Carthami, Radix Achyranthis Bidentatae |
| **157** | Rhodopsin | RHO | P08100 | Radix Bupleuri, Radix Paeoniae Rubra, Rhizoma Chuanxiong, Radix Angelicae Sinensis, Flos Carthami, Radix Platycodonis, Radix Achyranthis Bidentatae, Radix Rehmanniae |
| **158** | Cyclin-dependent kinase 2 | CDK2 | P24941 | Radix Bupleuri, Radix Paeoniae Rubra, Rhizoma Chuanxiong, Radix Angelicae Sinensis, Radix et Rhizoma Glycyrrhizae, Flos Carthami, Radix Platycodonis, Radix Achyranthis Bidentatae, Semen Persicae, Fructus Aurantii, Radix Rehmanniae |
| **159** | Tyrosine-protein kinase BTK | BTK | Q06187 | Rhizoma Chuanxiong, Flos Carthami |
| **160** | Phosphatidylinositol-4,5-bisphosphate 3-kinase catalytic subunit, gamma isoform | PIK3CG | P48736 | Radix Bupleuri, Radix Paeoniae Rubra, Rhizoma Chuanxiong, Radix Angelicae Sinensis, Radix et Rhizoma Glycyrrhizae, Flos Carthami, Radix Platycodonis, Radix Achyranthis Bidentatae, Semen Persicae, Radix Rehmanniae |
| **161** | Beta-lactamase | ampC | p00811 | Radix Bupleuri, Radix Paeoniae Rubra, Rhizoma Chuanxiong, Radix Angelicae Sinensis, Radix et Rhizoma Glycyrrhizae, Flos Carthami, Radix Platycodonis, Radix Achyranthis Bidentatae, Fructus Aurantii, Radix Rehmanniae |
| **164** | Leukotriene A-4 hydrolase | LTA4H | P09960 | Radix Bupleuri,Radix Paeoniae Rubra, Rhizoma Chuanxiong, Radix Angelicae Sinensis, Radix et Rhizoma Glycyrrhizae, Flos Carthami, Radix Achyranthis Bidentatae, Semen Persicae, Fructus Aurantii, Radix Rehmanniae |
| **165** | M-phase inducer phosphatase 2 | CDC25B | P30305 | Radix Angelicae Sinensis, Flos Carthami, Radix Achyranthis Bidentatae |
| **166** | Thromboxane-A synthase | TBXAS1 | P24557 | Rhizoma Chuanxiong |
| **167** | Thioredoxin reductase 1, cytoplasmic | TXNRD1 | Q16881 | Radix Angelicae Sinensis, Flos Carthami, Radix Achyranthis Bidentatae |
| **168** | Cholinesterase | BCHE | P06276 | Radix Bupleuri, Rhizoma Chuanxiong, Radix Angelicae Sinensis, Radix et Rhizoma Glycyrrhizae, Flos Carthami, Radix Platycodonis, Radix Achyranthis Bidentatae, Semen Persicae, Fructus Aurantii, Radix Rehmanniae |
| **169** | Amine oxidase [flavin-containing] B | MAOB | P27338 | Radix Bupleuri, Radix Paeoniae Rubra, Rhizoma Chuanxiong, Radix Angelicae Sinensis, Radix et Rhizoma Glycyrrhizae, Flos Carthami, Radix Platycodonis, Radix Achyranthis Bidentatae, Semen Persicae, Fructus Aurantii, Radix Rehmanniae |
| **170** | Amine oxidase [flavin-containing] A | MAOA | P21397 | Radix Bupleuri, Radix Paeoniae Rubra, Rhizoma Chuanxiong, Radix Angelicae Sinensis, Radix et Rhizoma Glycyrrhizae, Flos Carthami, Radix Achyranthis Bidentatae, Radix Rehmanniae |
| **171** | Xanthine dehydrogenase/oxidase | XDH | P47989 | Radix Bupleuri, Radix Angelicae Sinensis, Radix et Rhizoma Glycyrrhizae, Flos Carthami, Radix Platycodonis, Radix Achyranthis Bidentatae, Semen Persicae |
| **172** | Cyclin-dependent kinase 6 | CDK6 | Q00534 | Radix et Rhizoma Glycyrrhizae, Flos Carthami, Semen Persicae |
| **173** | Adenosine deaminase | ADA | P00813 | Rhizoma Chuanxiong, Flos Carthami |
| **174** | Cyclin-dependent kinase 4 | CDK4 | P11802 | Radix Bupleuri, Radix et Rhizoma Glycyrrhizae, Flos Carthami, Radix Platycodonis, Radix Achyranthis Bidentatae |
| **175** | Glutamyl aminopeptidase | ENPEP | Q07075 | Radix Bupleuri, Rhizoma Chuanxiong, Flos Carthami, |
| **176** | Neuronal acetylcholine receptor protein, alpha-7 chain | CHRNA7 | P36544 | Radix Bupleuri,Radix Paeoniae Rubra, Rhizoma Chuanxiong, Radix Angelicae Sinensis, Radix et Rhizoma Glycyrrhizae, Flos Carthami, Radix Achyranthis Bidentatae, Semen Persicae, Fructus Aurantii, Radix Rehmanniae |
| **177** | Prostaglandin E2 receptor EP3 subtype | PTGER3 | P43115 | Radix Bupleuri, Rhizoma Chuanxiong, Radix et Rhizoma Glycyrrhizae, Flos Carthami, Radix Achyranthis Bidentatae, Semen Persicae, Radix Rehmanniae |
| **178** | Superoxide dismutase [Cu-Zn] | SOD1 | P00441 | Radix Bupleuri, Radix Paeoniae Rubra, Rhizoma Chuanxiong, Radix et Rhizoma Glycyrrhizae, Flos Carthami, Radix Achyranthis Bidentatae |
| **179** | Tyrosine-protein kinase JAK2 | JAK2 | O60674 | Radix Bupleuri, Radix et Rhizoma Glycyrrhizae |
| **180** | Alpha-galactosidase A | GLA | P06280 | Radix Rehmanniae |
| **181** | Cytochrome P450 3A4 | CYP3A4 | P08684 | Radix Bupleuri, Radix et Rhizoma Glycyrrhizae, Flos Carthami, Radix Achyranthis Bidentatae |
| **182** | Pancreatic alpha-amylase | AMY2A | P04746 | Radix Bupleuri, Rhizoma Chuanxiong, Radix et Rhizoma Glycyrrhizae, Radix Achyranthis Bidentatae |
| **183** | Cytochrome P450 2A6 | CYP2A6 | P11509 | Radix Bupleuri |
| **184** | Cellular tumor antigen p53 | TP53 | P04637 | Radix Bupleuri, Radix Paeoniae Rubra, Rhizoma Chuanxiong, Radix et Rhizoma Glycyrrhizae, Flos Carthami, Radix Platycodonis, Radix Achyranthis Bidentatae, Semen Persicae, Radix Rehmanniae |
| **185** | Serine/threonine-protein kinase Chk1 | CHEK1 | O14757 | Radix Bupleuri, Radix Paeoniae Rubra, Rhizoma Chuanxiong, Radix Angelicae Sinensis, Radix et Rhizoma Glycyrrhizae, Flos Carthami, Radix Platycodonis, Radix Achyranthis Bidentatae, Semen Persicae, Fructus Aurantii |
| **186** | Amyloid beta A4 protein | APP | P05067 | Flos Carthami, Radix Platycodonis, |
| **187** | Transient receptor potential cation channel subfamily V member 3 | TRPV3 | Q8NET8 | Radix Bupleuri |
| **188** | Tyrosine-protein kinase JAK3 | JAK3 | P52333 | Radix Bupleuri |
| **189** | Cytochrome P450 2B6 | CYP2B6 | P20813 | Radix Bupleuri |
| **191** | Serine/threonine-protein kinase PLK1 | PLK1 | P53350 | Flos Carthami |
| **192** | Chymotrypsin-like elastase family member 1 | CELA1 | Q9UNI1 | Rhizoma Chuanxiong, Radix Angelicae Sinensis, Flos Carthami, Radix Achyranthis Bidentatae |
| **193** | mRNA of PKA Catalytic Subunit C-alpha | PRKACA | P17612 | Radix Bupleuri, Radix Paeoniae Rubra, Rhizoma Chuanxiong, Radix Angelicae Sinensis, Radix et Rhizoma Glycyrrhizae, Flos Carthami, Radix Platycodonis, Radix Achyranthis Bidentatae, Semen Persicae, Fructus Aurantii |
| **194** | Mitogen-activated protein kinase 9 | MAPK9 | P45984 | Radix Bupleuri, Radix Achyranthis Bidentatae |
| **195** | Mitogen-activated protein kinase 8 | MAPK8 | P45983 | Radix Bupleuri, Radix et Rhizoma Glycyrrhizae, Flos Carthami, Radix Achyranthis Bidentatae, Semen Persicae |
| **196** | Phospholipase A2 | PLA2G1B | P04054 | Radix Bupleuri, Rhizoma Chuanxiong, Radix Angelicae Sinensis, Flos Carthami, Radix Achyranthis Bidentatae, Radix Rehmanniae |
| **197** | Rhinovirus coat protein | HRV-1A | P23008 | Radix Bupleuri, Flos Carthami, Radix Achyranthis Bidentatae, Radix Rehmanniae |
| **198** | Hepatocyte nuclear factor 4-alpha | HNF4A | P41235 | Flos Carthami |
| **199** | Lactase-phlorizin hydrolase | LCT | P09848 | Radix Bupleuri, Rhizoma Chuanxiong, Radix et Rhizoma Glycyrrhizae, Radix Platycodonis, |
| **200** | Cytochrome P450 1A2 | CYPIA2 | P05177 | Radix Bupleuri, Rhizoma Chuanxiong, Radix et Rhizoma Glycyrrhizae, Flos Carthami, Radix Achyranthis Bidentatae |
| **201** | Stromelysin-2 | MMP10 | P09238 | Radix Et Rhizoma Glycyrrhizae, Flos Carthami, Semen Persicae |
| **202** | Alcohol dehydrogenase 1B | ADH1B | P00325 | Radix Bupleuri, Radix Paeoniae Rubra, Rhizoma Chuanxiong, Radix Angelicae Sinensis, Radix et Rhizoma Glycyrrhizae, Flos Carthami, Radix Platycodonis, Radix Achyranthis Bidentatae, Fructus Aurantii, Radix Rehmanniae |
| **203** | Alcohol dehydrogenase 1C | ADH1C | P00326 | Radix Bupleuri, Radix Paeoniae Rubra, Rhizoma Chuanxiong, Radix Angelicae Sinensis, Radix et Rhizoma Glycyrrhizae, Flos Carthami, Radix Platycodonis, Radix Achyranthis Bidentatae, Semen Persicae, Fructus Aurantii, Radix Rehmanniae |
| **205** | Alcohol dehydrogenase 1A | ADH1A | P07327 | Radix Bupleuri, Radix Paeoniae Rubra, Rhizoma Chuanxiong, Radix Angelicae Sinensis, Radix et Rhizoma Glycyrrhizae, Flos Carthami, Radix Platycodonis, Radix Achyranthis Bidentatae, Fructus Aurantii, Radix Rehmanniae |
| **206** | Arginase-1 | ARG1 | P05089 | Rhizoma Chuanxiong, Radix Achyranthis Bidentatae |
| **207** | Collagen alpha-1(I) chain | COL1A1 | P02452 | Radix Bupleuri, Radix Paeoniae Rubra, Rhizoma Chuanxiong, Radix Angelicae Sinensis, Radix et Rhizoma Glycyrrhizae, Flos Carthami, Radix Platycodonis, Radix Achyranthis Bidentatae |
| **208** | Glutathione S-transferase P | GSTP1 | P09211 | Radix Bupleuri, Radix Paeoniae Rubra, Radix et Rhizoma Glycyrrhizae, Flos Carthami, Radix Platycodonis, Radix Achyranthis Bidentatae, Radix Rehmanniae |
| **209** | Pro-epidermal growth factor | EGF | P01133 | Radix Bupleuri, Radix et Rhizoma Glycyrrhizae, Flos Carthami, Radix Achyranthis Bidentatae |
| **210** | Catalase | CAT | P04040 | Radix Bupleuri, Radix Paeoniae Rubra, Rhizoma Chuanxiong, Radix Angelicae Sinensis, Radix et Rhizoma Glycyrrhizae, Flos Carthami, Radix Achyranthis Bidentatae, Radix Rehmanniae |
| **211** | Vascular endothelial growth factor A | VEGFA | P15692 | Radix Bupleuri, Radix Paeoniae Rubra, Rhizoma Chuanxiong, Radix et Rhizoma Glycyrrhizae, Flos Carthami, Radix Platycodonis, Radix Achyranthis Bidentatae, Semen Persicae, Radix Rehmanniae |
| **213** | NADPH--cytochrome P450 reductase | POR | P16435 | Radix Bupleuri, Radix et Rhizoma Glycyrrhizae, Flos Carthami, Radix Achyranthis Bidentatae |
| **214** | Ribonucleoside-diphosphate reductase large subunit | RRM1 | P23921 | Flos Carthami, Radix Achyranthis Bidentatae |
| **215** | Pyruvate kinase isozymes R/L | PKLR | P30613 | Radix Paeoniae Rubra, Flos Carthami |
| **216** | Dihydroorotate dehydrogenase (quinone), mitochondrial | DHODH | Q02127 | Radix Achyranthis Bidentatae |
| **217** | Prostacyclin synthase | PTGIS | Q16647 | Radix Paeoniae Rubra, Flos Carthami, |
| **218** | Glutathione S-transferase A1 | GSTA1 | P08263 | Radix Paeoniae Rubra, Rhizoma Chuanxiong, Radix Rehmanniae |
| **219** | BDNF/NT-3 growth factors receptor | NTRK2 | Q16620 | Radix Bupleuri, Rhizoma Chuanxiong, Flos Carthami, |
| **220** | Alcohol dehydrogenase class 4 mu/sigma chain | ADH7 | P40394 | Radix Achyranthis Bidentatae |
| **221** | Interferon beta | IFNB1 | P01574 | Radix Bupleuri, Radix Paeoniae Rubra, Flos Carthami, |
| **222** | Estrogen sulfotransferase | SULT1E1 | P49888 | Radix Bupleuri, Radix et Rhizoma Glycyrrhizae, Flos Carthami, Radix Achyranthis Bidentatae |
| **223** | Aldehyde dehydrogenase, dimeric NADP-preferring | ALDH3A1 | P30838 | Radix Achyranthis Bidentatae |
| **224** | High affinity immunoglobulin epsilon receptor subunit beta | MS4A2 | Q01362 | Flos Carthami |
| **225** | Carboxylesterase 2 | estB | Q53547 | Radix Achyranthis Bidentatae |
| **226** | Retinoic acid receptor RXR-beta | RXRB | P28702 | Radix et Rhizoma Glycyrrhizae |
| **227** | Streptavidin | Streptavidin | P22629 | Radix Achyranthis Bidentatae |
| **228** | P-hydroxybenzoate hydroxylase | pobA | P00438 | Rhizoma Chuanxiong |
