# Supplementary material for: Natural formulas and the nature of formulas: Exploring potential therapeutic targets based on traditional Chinese herbal formulas
Source: PLoS One. 2017 Feb 9;12(2):e0171628. doi: 10.1371/journal.pone.0171628 (PMC5300118; doi:10.1371/journal.pone.0171628)
Supplement: S5 Table — (DOCX) [file pone.0171628.s005.docx]

S5 Table. Target proteins of herbal medicines in GXBD

| ID | Proteins | Synonyms | UniProtKB | Herbal Medicines |
| --- | --- | --- | --- | --- |
| 1 | Nitric oxide synthase, inducible | NOS2 | P35228 | Fructus Trichosanthis, Bulbus Allium Macrostemonis, Rhizoma Pinelliae |
| 2 | Prostaglandin G/H synthase 1 | PTGS1 | P23219 | Fructus Trichosanthis, Bulbus Allium Macrostemonis, Rhizoma Pinelliae |
| 3 | D(1A) dopamine receptor | DRD1 | P21728 | Fructus Trichosanthis, Bulbus Allium Macrostemonis, Rhizoma Pinelliae |
| 4 | Insulin receptor | INSR | P06213 | Bulbus Allium Macrostemonis |
| 5 | Muscarinic acetylcholine receptor M3 | CHRM3 | P20309 | Fructus Trichosanthis, Bulbus Allium Macrostemonis, Rhizoma Pinelliae |
| 6 | prothrombin | F2 | P00734 | Fructus Trichosanthis, Bulbus Allium Macrostemonis, Rhizoma Pinelliae |
| 8 | Nitric oxide synthase, brain | NOS1 | P29475 | Fructus Trichosanthis, Bulbus Allium Macrostemonis, Rhizoma Pinelliae |
| 9 | Potassium voltage-gated channel subfamily H member 2 | KCNA4 | Q9H252 | Fructus Trichosanthis, Bulbus Allium Macrostemonis, Rhizoma Pinelliae |
| 10 | Muscarinic acetylcholine receptor M1 | CHRM1 | P11229 | Fructus Trichosanthis, Bulbus Allium Macrostemonis, Rhizoma Pinelliae |
| 11 | S-adenosylmethionine decarboxylase proenzyme | AMD1 | P17707 | Fructus Trichosanthis, Rhizoma Pinelliae |
| 12 | Estrogen receptor | ESR1 | P03372 | Fructus Trichosanthis, Bulbus Allium Macrostemonis, Rhizoma Pinelliae |
| 13 | Androgen receptor | AR | P10275 | Fructus Trichosanthis, Bulbus Allium Macrostemonis, Rhizoma Pinelliae |
| 14 | Aldehyde dehydrogenase, mitochondrial | ALDH2 | P05091 | Fructus Trichosanthis, Rhizoma Pinelliae |
| 15 | Succinate semialdehyde dehydrogenase, mitochondrial | ALDH5A1 | P51649 | Fructus Trichosanthis, Bulbus Allium Macrostemonis, Rhizoma Pinelliae |
| 16 | Beta-1 adrenergic receptor | ADRB1 | P08588 | Fructus Trichosanthis, Bulbus Allium Macrostemonis, Rhizoma Pinelliae |
| 19 | Sodium channel protein type 5 subunit alpha | SCN5A | Q14524 | Fructus Trichosanthis, Bulbus Allium Macrostemonis, Rhizoma Pinelliae |
| 21 | Plasminogen | PLG | P00747 | Fructus Trichosanthis, Bulbus Allium Macrostemonis, Rhizoma Pinelliae |
| 22 | High-affinity cationic amino acid transporter-1 | SLC7A1 | P30825 | Fructus Trichosanthis, Rhizoma Pinelliae |
| 23 | Peroxisome proliferator-activated receptor gamma | PPARG | P37231 | Fructus Trichosanthis, Bulbus Allium Macrostemonis, Rhizoma Pinelliae |
| 24 | Coagulation factor X | F10 | P00742 | Fructus Trichosanthis, Bulbus Allium Macrostemonis, Rhizoma Pinelliae |
| 26 | Lipoprotein lipase | LPL | P06858 | Fructus Trichosanthis, Bulbus Allium Macrostemonis, Rhizoma Pinelliae |
| 27 | Apoptosis regulator Bcl-2 | BCL2 | P10415 | Fructus Trichosanthis, Bulbus Allium Macrostemonis, Rhizoma Pinelliae |
| 28 | Muscarinic acetylcholine receptor M5 | CHRM5 | p08912 | Rhizoma Pinelliae |
| 29 | Arachidonate 5-lipoxygenase | ALOX5 | P09917 | Fructus Trichosanthis, Bulbus Allium Macrostemonis, Rhizoma Pinelliae |
| 30 | Sodium-and chloride-dependent GABA transporter 1 | SLC6A1 | P30531 | Rhizoma Pinelliae |
| 31 | Purine nucleoside phosphorylase | PNP | P00491 | Bulbus Allium Macrostemonis, Rhizoma Pinelliae |
| 32 | 4-aminobutyrate aminotransferase, mitochondrial | ABAT | P80404 | Fructus Trichosanthis, Bulbus Allium Macrostemonis, Rhizoma Pinelliae |
| 33 | Prostaglandin G/H synthase 2 | PTGS2 | P35354 | Fructus Trichosanthis, Bulbus Allium Macrostemonis, Rhizoma Pinelliae |
| 34 | Nitric-oxide synthase, endothelial | NOS3 | P29474 | Fructus Trichosanthis, Bulbus Allium Macrostemonis, Rhizoma Pinelliae |
| 36 | Renin | REN | P00797 | Fructus Trichosanthis, Rhizoma Pinelliae |
| 37 | Alpha-2A adrenergic receptor | ADRA2A | P08913 | Fructus Trichosanthis, Bulbus Allium Macrostemonis, Rhizoma Pinelliae |
| 38 | Serine hydroxymethyltransferase, mitochondrial | SHMT2 | P34897 | Fructus Trichosanthis, Rhizoma Pinelliae |
| 39 | DNA-dependent protein kinase catalytic subunit | PRKDC | P78527 | Fructus Trichosanthis, Rhizoma Pinelliae |
| 40 | 5-hydroxytryptamine receptor 3A | HTR3A | P46098 | Bulbus Allium Macrostemonis, Rhizoma Pinelliae |
| 41 | Carbonic anhydrase II | CA2 | P00918 | Fructus Trichosanthis, Bulbus Allium Macrostemonis, Rhizoma Pinelliae |
| 42 | Coagulation factor VII | F7 | P08709 | Fructus Trichosanthis, Bulbus Allium Macrostemonis, Rhizoma Pinelliae |
| 43 | Urease subunit alpha | ureC | P41020 | Fructus Trichosanthis, Rhizoma Pinelliae |
| 44 | Alpha-2C adrenergic receptor | ADRA2C | P18825 | Fructus Trichosanthis, Rhizoma Pinelliae |
| 45 | DNA polymerase catalytic subunit | UL54 | Q9WHQ7 | Fructus Trichosanthis, Rhizoma Pinelliae |
| 46 | Glutamate receptor ionotropic, NMDA 1 | GRIN1 | Q05586 | Fructus Trichosanthis, Rhizoma Pinelliae |
| 49 | Gamma-aminobutyric-acid receptor alpha-2 subunit | GABRA2 | P47869 | Fructus Trichosanthis, Bulbus Allium Macrostemonis, Rhizoma Pinelliae |
| 51 | Kynureninase | KYNU | Q16719 | Fructus Trichosanthis, Rhizoma Pinelliae |
| 52 | Ornithine decarboxylase | ODC1 | P11926 | Bulbus Allium Macrostemonis, Rhizoma Pinelliae |
| 53 | Muscarinic acetylcholine receptor M4 | CHRM4 | P08173 | Bulbus Allium Macrostemonis, Rhizoma Pinelliae |
| 54 | Retinoic acid receptor RXR-alpha | RXRA | P19793 | Fructus Trichosanthis, Bulbus Allium Macrostemonis, Rhizoma Pinelliae |
| 55 | Glutamate receptor ionotropic, NMDA 2B | GRIN2B | Q13224 | Fructus Trichosanthis, Rhizoma Pinelliae |
| 56 | Delta-type opioid receptor | OPRD1 | P41143 | Rhizoma Pinelliae |
| 57 | Ornithine aminotransferase, mitochondrial | OAT | P04181 | Fructus Trichosanthis, Bulbus Allium Macrostemonis, Rhizoma Pinelliae |
| 58 | Acetylcholinesterase | ACHE | P22303 | Fructus Trichosanthis, Bulbus Allium Macrostemonis, Rhizoma Pinelliae |
| 59 | Glycine receptor subunit alpha-1 | GLRA1 | P23415 | Fructus Trichosanthis, Bulbus Allium Macrostemonis, Rhizoma Pinelliae |
| 60 | cGMP-inhibited 3',5'-cyclic phosphodiesterase A | PDE3A | Q14432 | Bulbus Allium Macrostemonis, Rhizoma Pinelliae |
| 61 | 5-hydroxytryptamine receptor 2A | HTR2A | P28223 | Bulbus Allium Macrostemonis, Rhizoma Pinelliae |
| 62 | Gamma-aminobutyric-acid receptor alpha-5 subunit | GABRA5 | P31644 | Fructus Trichosanthis, Bulbus Allium Macrostemonis, Rhizoma Pinelliae |
| 63 | Sodium-dependent noradrenaline transporter | SLC6A2 | P23975 | Fructus Trichosanthis, Bulbus Allium Macrostemonis, Rhizoma Pinelliae |
| 64 | Low-density lipoprotein receptor | LDLR | P01130 | Bulbus Allium Macrostemonis |
| 65 | Alpha-1A adrenergic receptor | ADRA1A | P35348 | Fructus Trichosanthis, Bulbus Allium Macrostemonis, Rhizoma Pinelliae |
| 67 | Gamma-aminobutyric-acid receptor alpha-3 subunit | GABRA3 | P34903 | Fructus Trichosanthis, Bulbus Allium Macrostemonis, Rhizoma Pinelliae |
| 68 | Aspartate aminotransferase, cytoplasmic | GOT1 | P17174 | Fructus Trichosanthis, Bulbus Allium Macrostemonis, Rhizoma Pinelliae |
| 69 | 5-hydroxytryptamine receptor 2C | HTR2C | P28335 | Rhizoma Pinelliae |
| 71 | Progesterone receptor | PGR | P06401 | Fructus Trichosanthis, Bulbus Allium Macrostemonis, Rhizoma Pinelliae |
| 72 | Muscarinic acetylcholine receptor M2 | CHRM2 | P08172 | Fructus Trichosanthis, Bulbus Allium Macrostemonis, Rhizoma Pinelliae |
| 73 | Alpha-2B adrenergic receptor | ADRA2B | P18089 | Fructus Trichosanthis, Bulbus Allium Macrostemonis, Rhizoma Pinelliae |
| 74 | Alpha-1B adrenergic receptor | ADRA1B | P35368 | Fructus Trichosanthis, Bulbus Allium Macrostemonis, Rhizoma Pinelliae |
| 77 | mRNA of Protein-tyrosine phosphatase, non-receptor type 1 | PTPN1 | P18031 | Fructus Trichosanthis, Bulbus Allium Macrostemonis, Rhizoma Pinelliae |
| 78 | Acetyl-CoA carboxylase 1 | ACACA | Q13085 | Bulbus Allium Macrostemonis |
| 80 | Glutamate receptor 1 | GRIA1 | P42261 | Fructus Trichosanthis, Rhizoma Pinelliae |
| 81 | 72 kDa type IV collagenase | MMP2 | P08253 | Bulbus Allium Macrostemonis |
| 82 | Sodium-dependent dopamine transporter | SLC6A3 | Q01959 | Fructus Trichosanthis, Bulbus Allium Macrostemonis, Rhizoma Pinelliae |
| 83 | Glutathione reductase, mitochondrial | GSR | P00390 | Bulbus Allium Macrostemonis |
| 85 | Mineralocorticoid receptor | NR3C2 | P08235 | Fructus Trichosanthis, Bulbus Allium Macrostemonis, Rhizoma Pinelliae |
| 86 | Beta-2 adrenergic receptor | ADRB2 | P07550 | Fructus Trichosanthis, Bulbus Allium Macrostemonis, Rhizoma Pinelliae |
| 87 | Tumor necrosis factor | TNF | P01375 | Fructus Trichosanthis, Bulbus Allium Macrostemonis, Rhizoma Pinelliae |
| 88 | Retinoic acid receptor RXR-gamma | RXRG | P48443 | Fructus Trichosanthis, Rhizoma Pinelliae |
| 89 | Alpha-1D adrenergic receptor | ADRA1D | P25100 | Bulbus Allium Macrostemonis, Rhizoma Pinelliae |
| 90 | Branched-chain-amino-acid aminotransferase, mitochondrial | BCAT2 | O15382 | Fructus Trichosanthis, Bulbus Allium Macrostemonis, Rhizoma Pinelliae |
| 91 | Neuronal acetylcholine receptor subunit alpha-2 | CHRNA2 | Q15822 | Bulbus Allium Macrostemonis, Rhizoma Pinelliae |
| 92 | DNA topoisomerase 2-alpha | TOP2A | P11388 | Fructus Trichosanthis, Bulbus Allium Macrostemonis, Rhizoma Pinelliae |
| 93 | Aldose reductase | AKR1B1 | P15121 | Fructus Trichosanthis, Bulbus Allium Macrostemonis, Rhizoma Pinelliae |
| 94 | Sodium-dependent serotonin transporter | SLC6A4 | P31645 | Bulbus Allium Macrostemonis, Rhizoma Pinelliae |
| 96 | Arginase II, mitochondrial | ARG2 | P78540 | Fructus Trichosanthis, Rhizoma Pinelliae |
| 98 | Glutamate receptor ionotropic, NMDA 2A | GRIN2A | Q12879 | Fructus Trichosanthis, Rhizoma Pinelliae |
| 99 | Epidermal growth factor receptor | EGFR | P00533 | Bulbus Allium Macrostemonis |
| 100 | Mu-type opioid receptor | OPRM1 | P35372 | Bulbus Allium Macrostemonis, Rhizoma Pinelliae |
| 101 | Multidrug resistance-associated protein 1 | ABCC1 | P33527 | Bulbus Allium Macrostemonis |
| 102 | Estrogen receptor beta | ESR2 | Q92731 | Fructus Trichosanthis, Bulbus Allium Macrostemonis, Rhizoma Pinelliae |
| 103 | Glucocorticoid receptor | NR3C1 | P04150 | Fructus Trichosanthis, Bulbus Allium Macrostemonis, Rhizoma Pinelliae |
| 104 | Gamma-aminobutyric acid receptor subunit alpha-1 | GABRA1 | P14867 | Fructus Trichosanthis, Bulbus Allium Macrostemonis, Rhizoma Pinelliae |
| 105 | Maltase-glucoamylase, intestinal | MGAM | O43451 | Bulbus Allium Macrostemonis, Rhizoma Pinelliae |
| 106 | Proto-oncogene tyrosine-protein kinase SRC | SRC | P12931 | Fructus Trichosanthis, Bulbus Allium Macrostemonis, Rhizoma Pinelliae |
| 107 | T-lymphocyte activation antigen CD86 | CD86 | P42081 | Fructus Trichosanthis, Bulbus Allium Macrostemonis |
| 108 | T-lymphocyte activation antigen CD80 | CD80 | P33681 | Fructus Trichosanthis, Bulbus Allium Macrostemonis |
| 109 | Dipeptidyl peptidase 4 | DPP4 | P27487 | Fructus Trichosanthis, Bulbus Allium Macrostemonis, Rhizoma Pinelliae |
| 111 | Urokinase-type plasminogen activator | PLAU | P00749 | Fructus Trichosanthis, Bulbus Allium Macrostemonis, Rhizoma Pinelliae |
| 113 | Glycogen phosphorylase, muscle form | PYGM | P11217 | Fructus Trichosanthis, Rhizoma Pinelliae |
| 114 | Interleukin-6 | IL6 | P05231 | Fructus Trichosanthis, Bulbus Allium Macrostemonis, Rhizoma Pinelliae |
| 115 | Interstitial collagenase | MMP1 | P03956 | Bulbus Allium Macrostemonis |
| 116 | Mitogen-activated protein kinase 1 | MAPK1 | P28482 | Fructus Trichosanthis, Bulbus Allium Macrostemonis |
| 117 | Adenosine receptor A2a | ADORA2A | P29274 | Bulbus Allium Macrostemonis |
| 118 | Serum paraoxonase/arylesterase 1 | PON1 | P27169 | Fructus Trichosanthis, Bulbus Allium Macrostemonis, Rhizoma Pinelliae |
| 120 | Sodium-and chloride-dependent glycine transporter 1 | SLC6A9 | P48067 | Fructus Trichosanthis, Rhizoma Pinelliae |
| 121 | Cathepsin D | CTSD | P07339 | Fructus Trichosanthis, Bulbus Allium Macrostemonis, Rhizoma Pinelliae |
| 122 | Interferon gamma | IFNG | P01579 | Bulbus Allium Macrostemonis |
| 123 | Fatty acid synthase | FASN | P49327 | Bulbus Allium Macrostemonis, Rhizoma Pinelliae |
| 124 | catenin Beta-1 | CTNNB1 | P35222 | Fructus Trichosanthis, Rhizoma Pinelliae |
| 126 | Lactotransferrin | LTF | P02788 | Fructus Trichosanthis, Rhizoma Pinelliae |
| 129 | Sodium-and chloride-dependent glycine transporter 2 | SLC6A5 | Q9Y345 | Fructus Trichosanthis |
| 131 | Mitogen-activated protein kinase 14 | MAPK14 | Q16539 | Fructus Trichosanthis, Bulbus Allium Macrostemonis, Rhizoma Pinelliae |
| 132 | Transient receptor potential cation channel subfamily V member 1 | TRPV1 | Q8NER1 | Fructus Trichosanthis, Bulbus Allium Macrostemonis, Rhizoma Pinelliae |
| 135 | Transcription factor AP-1 | JUN | P05412 | Fructus Trichosanthis, Bulbus Allium Macrostemonis, Rhizoma Pinelliae |
| 137 | C-C motif chemokine 2 | CCL2 | P13500 | Bulbus Allium Macrostemonis |
| 138 | Interleukin-1 beta | IL1B | P01584 | Bulbus Allium Macrostemonis |
| 139 | Mitogen-activated protein kinase 3 | MAPK3 | P27361 | Bulbus Allium Macrostemonis |
| 140 | Glycogen synthase kinase-3 beta | GSK3B | P49841 | Fructus Trichosanthis, Bulbus Allium Macrostemonis, Rhizoma Pinelliae |
| 141 | E-selectin | SELE | P16581 | Bulbus Allium Macrostemonis |
| 142 | Myeloperoxidase | MPO | P05164 | Fructus Trichosanthis, Bulbus Allium Macrostemonis, Rhizoma Pinelliae |
| 143 | Cell division control protein 2 homolog | CDK1 | p06493 | Bulbus Allium Macrostemonis, Rhizoma Pinelliae |
| 144 | Tissue-type plasminogen activator | PLAT | P00750 | Bulbus Allium Macrostemonis |
| 145 | Gap junction alpha-1 protein | GJA1 | P17302 | Bulbus Allium Macrostemonis |
| 147 | Vascular cell adhesion protein 1 | VCAM1 | P19320 | Bulbus Allium Macrostemonis |
| 148 | Stromelysin-1 | MMP3 | P08254 | Bulbus Allium Macrostemonis |
| 149 | Heat shock protein HSP 90-alpha | HSP90AA1 | P07900 | Fructus Trichosanthis, Bulbus Allium Macrostemonis, Rhizoma Pinelliae |
| 150 | Thrombomodulin | THBD | P07204 | Bulbus Allium Macrostemonis |
| 151 | Inhibitor of nuclear factor kappa-B kinase subunit beta | IKBKB | O14920 | Fructus Trichosanthis, Rhizoma Pinelliae |
| 152 | P-selectin | SELP | P16109 | Rhizoma Pinelliae |
| 153 | Tissue factor | F3 | P13726 | Bulbus Allium Macrostemonis |
| 154 | Neutrophil collagenase | MMP8 | P22894 | Fructus Trichosanthis, Rhizoma Pinelliae |
| 155 | NAD(P)H dehydrogenase [quinone] 1 | NQO1 | P15559 | Bulbus Allium Macrostemonis |
| 156 | Macrophage metalloelastase | MMP12 | P39900 | Fructus Trichosanthis, Rhizoma Pinelliae |
| 157 | Rhodopsin | RHO | P08100 | Fructus Trichosanthis, Bulbus Allium Macrostemonis, Rhizoma Pinelliae |
| 158 | Cyclin-dependent kinase 2 | CDK2 | P24941 | Fructus Trichosanthis, Bulbus Allium Macrostemonis, Rhizoma Pinelliae |
| 159 | Tyrosine-protein kinase BTK | BTK | Q06187 | Rhizoma Pinelliae |
| 160 | Phosphatidylinositol-4,5-bisphosphate 3-kinase catalytic subunit, gamma isoform | PIK3CG | P48736 | Fructus Trichosanthis, Bulbus Allium Macrostemonis, Rhizoma Pinelliae |
| 161 | Beta-lactamase | ampC | p00811 | Fructus Trichosanthis, Bulbus Allium Macrostemonis, Rhizoma Pinelliae |
| 162 | Dihydroorotase | pyrC | P05020 | Fructus Trichosanthis, Rhizoma Pinelliae |
| 163 | UDP-glucose 4-epimerase | GALE | Q14376 | Fructus Trichosanthis, Rhizoma Pinelliae |
| 164 | Leukotriene A-4 hydrolase | LTA4H | P09960 | Fructus Trichosanthis, Bulbus Allium Macrostemonis, Rhizoma Pinelliae |
| 165 | M-phase inducer phosphatase 2 | CDC25B | P30305 | Fructus Trichosanthis, Bulbus Allium Macrostemonis, Rhizoma Pinelliae |
| 167 | Thioredoxin reductase 1, cytoplasmic | TXNRD1 | Q16881 | Fructus Trichosanthis, Bulbus Allium Macrostemonis, Rhizoma Pinelliae |
| 168 | Cholinesterase | BCHE | P06276 | Fructus Trichosanthis, Bulbus Allium Macrostemonis, Rhizoma Pinelliae |
| 169 | Amine oxidase [flavin-containing] B | MAOB | P27338 | Fructus Trichosanthis, Bulbus Allium Macrostemonis, Rhizoma Pinelliae |
| 170 | Amine oxidase [flavin-containing] A | MAOA | P21397 | Fructus Trichosanthis, Bulbus Allium Macrostemonis, Rhizoma Pinelliae |
| 171 | Xanthine dehydrogenase/oxidase | XDH | P47989 | Fructus Trichosanthis, Bulbus Allium Macrostemonis, Rhizoma Pinelliae |
| 173 | Adenosine deaminase | ADA | P00813 | Bulbus Allium Macrostemonis |
| 175 | Glutamyl aminopeptidase | ENPEP | Q07075 | Fructus Trichosanthis, Bulbus Allium Macrostemonis, Rhizoma Pinelliae |
| 176 | Neuronal acetylcholine receptor protein, alpha-7 chain | CHRNA7 | P36544 | Bulbus Allium Macrostemonis, Rhizoma Pinelliae |
| 177 | Prostaglandin E2 receptor EP3 subtype | PTGER3 | P43115 | Fructus Trichosanthis, Bulbus Allium Macrostemonis |
| 178 | Superoxide dismutase [Cu-Zn] | SOD1 | P00441 | Fructus Trichosanthis, Bulbus Allium Macrostemonis, Rhizoma Pinelliae |
| 179 | Tyrosine-protein kinase JAK2 | JAK2 | O60674 | Bulbus Allium Macrostemonis |
| 181 | Cytochrome P450 3A4 | CYP3A4 | P08684 | Bulbus Allium Macrostemonis |
| 182 | Pancreatic alpha-amylase | AMY2A | P04746 | Fructus Trichosanthis, Rhizoma Pinelliae |
| 184 | Cellular tumor antigen p53 | TP53 | P04637 | Bulbus Allium Macrostemonis, Rhizoma Pinelliae |
| 185 | Serine/threonine-protein kinase Chk1 | CHEK1 | O14757 | Fructus Trichosanthis, Bulbus Allium Macrostemonis, Rhizoma Pinelliae |
| 190 | V-type proton ATPase catalytic subunit A | ATP6V1A | P38606 | Fructus Trichosanthis, Rhizoma Pinelliae |
| 192 | Chymotrypsin-like elastase family member 1 | CELA1 | Q9UNI1 | Fructus Trichosanthis, Rhizoma Pinelliae |
| 193 | mRNA of PKA Catalytic Subunit C-alpha | PRKACA | P17612 | Fructus Trichosanthis, Bulbus Allium Macrostemonis, Rhizoma Pinelliae |
| 196 | Phospholipase A2 | PLA2G1B | P04054 | Fructus Trichosanthis, Bulbus Allium Macrostemonis, Rhizoma Pinelliae |
| 197 | Rhinovirus coat protein | HRV-1A | P23008 | Fructus Trichosanthis, Bulbus Allium Macrostemonis |
| 198 | Hepatocyte nuclear factor 4-alpha | HNF4A | P41235 | Fructus Trichosanthis, Rhizoma Pinelliae |
| 200 | Cytochrome P450 1A2 | CYPIA2 | P05177 | Bulbus Allium Macrostemonis |
| 202 | Alcohol dehydrogenase 1B | ADH1B | P00325 | Fructus Trichosanthis, Bulbus Allium Macrostemonis, Rhizoma Pinelliae |
| 203 | Alcohol dehydrogenase 1C | ADH1C | P00326 | Fructus Trichosanthis, Bulbus Allium Macrostemonis, Rhizoma Pinelliae |
| 204 | Retinal dehydrogenase 1 | ALDH1A1 | P00352 | Fructus Trichosanthis, Rhizoma Pinelliae |
| 205 | Alcohol dehydrogenase 1A | ADH1A | P07327 | Fructus Trichosanthis, Bulbus Allium Macrostemonis, Rhizoma Pinelliae |
| 206 | Arginase-1 | ARG1 | P05089 | Fructus Trichosanthis, Rhizoma Pinelliae |
| 207 | Collagen alpha-1(I) chain | COL1A1 | P02452 | Fructus Trichosanthis, Bulbus Allium Macrostemonis, Rhizoma Pinelliae |
| 208 | Glutathione S-transferase P | GSTP1 | P09211 | Bulbus Allium Macrostemonis |
| 209 | Pro-epidermal growth factor | EGF | P01133 | Bulbus Allium Macrostemonis |
| 210 | Catalase | CAT | P04040 | Fructus Trichosanthis, Bulbus Allium Macrostemonis, Rhizoma Pinelliae |
| 211 | Vascular endothelial growth factor A | VEGFA | P15692 | Bulbus Allium Macrostemonis, Rhizoma Pinelliae |
| 212 | Glycine dehydrogenase [decarboxylating], mitochondrial | GLDC | P23378 | Fructus Trichosanthis, Rhizoma Pinelliae |
| 213 | NADPH--cytochrome P450 reductase | POR | P16435 | Bulbus Allium Macrostemonis |
| 214 | Ribonucleoside-diphosphate reductase large subunit | RRM1 | P23921 | Fructus Trichosanthis, Rhizoma Pinelliae |
| 215 | Pyruvate kinase isozymes R/L | PKLR | P30613 | Fructus Trichosanthis, Rhizoma Pinelliae |
| 216 | Dihydroorotate dehydrogenase (quinone), mitochondrial | DHODH | Q02127 | Fructus Trichosanthis, Rhizoma Pinelliae |
| 219 | BDNF/NT-3 growth factors receptor | NTRK2 | Q16620 | Fructus Trichosanthis, Bulbus Allium Macrostemonis, Rhizoma Pinelliae |
| 222 | Estrogen sulfotransferase | SULT1E1 | P49888 | Fructus Trichosanthis, Bulbus Allium Macrostemonis |
| 226 | Retinoic acid receptor RXR-beta | RXRB | P28702 | Rhizoma Pinelliae |
| 228 | P-hydroxybenzoate hydroxylase | pobA | P00438 | Bulbus Allium Macrostemonis, Rhizoma Pinelliae |
