# Supplementary material for: Natural formulas and the nature of formulas: Exploring potential therapeutic targets based on traditional Chinese herbal formulas
Source: PLoS One. 2017 Feb 9;12(2):e0171628. doi: 10.1371/journal.pone.0171628 (PMC5300118; doi:10.1371/journal.pone.0171628)
Supplement: S6 Table — (DOCX) [file pone.0171628.s006.docx]

S6 Table. Target proteins of anti-CAD drugs

| ID | Target proteins | UniprotKB | Anti-CAD drugs |
| --- | --- | --- | --- |
| 1 | ABCA1 | O95477 | Probucol |
| 2 | ACCN2 | P78348 | Amiloride |
| 3 | ACE | P12821 | Benazepril, Bendroflumethiazide, Candesartan, Captopril, Cilazapril, Delapril, Enalapril, Fosinopril, Imidapril, Lisinopril, Metolazone, Moexipril, Perindopril, Practolol, Quinapril, Ramipril, Spirapril, Telmisartan, Temocapril, Trandolapril, Zofenopril |
| 4 | ACE2 | Q9BYF1 | Moexipril |
| 5 | ACHE | P22303 | Ephedrine |
| 6 | ACSL3 | O95573 | Icosapent |
| 7 | ACSL4 | O60488 | Icosapent |
| 8 | ADA | P00813 | Dipyridamole |
| 9 | ADORA1 | P30542 | Defibrotide, Theobromine |
| 10 | ADORA2A | P29274 | Defibrotide, Regadenoson, Theobromine |
| 11 | ADORA2B | P29275 | Defibrotide |
| 12 | ADRA1A | P35348 | Bevantolol, Carvedilol, Ephedrine, Labetalol, Nicardipine |
| 13 | ADRA1B | P35368 | Carvedilol, Labetalol, Nicardipine |
| 14 | ADRA1D | P25100 | Carvedilol, Labetalol, Nicardipine |
| 15 | ADRA2A | P08913 | Carvedilol |
| 16 | ADRA2B | P18089 | Carvedilol |
| 17 | ADRA2C | P18825 | Carvedilol |
| 18 | ADRB1 | P08588 | Acebutolol, Alprenolol, Atenolol, Betaxolol, Bevantolol, Bisoprolol, Bopindolol, Bupranolol, Carteolol, Carvedilol, Celiprolol, Cloranolol, Epanolol, Esmolol, Labetalol, Mepindolol, Metoprolol, Nadolol, Nebivolol, Oxprenolol, Penbutolol, Pindolol, Practolol, Propranolol, S-atenolol, Sotalol, Talinolol, Tertatolol, Timolol |
| 19 | ADRB2 | P07550 | Acebutolol, Alprenolol, Atenolol, Betaxolol, Bevantolol, Bisoprolol, Bopindolol, Bupranolol, Carteolol, Carvedilol, Cloranolol, Labetalol, Mepindolol, Metoprolol, Nadolol, Nebivolol, Orciprenaline, Oxprenolol, Penbutolol, Pindolol, Propranolol, Sotalol, Tertatolol, Timolol |
| 20 | ADRB3 | P13945 | Alprenolol, Bopindolol, Bupranolol, Carteolol, Cloranolol, Labetalol, Mepindolol, Nadolol, Oxprenolol, Penbutolol, Pindolol, Propranolol, Tertatolol, Timolol |
| 21 | AGTR1 | P30556 | Azilsartan medoxomil, Candesartan, Eprosartan, Irbesartan, Losartan, Olmesartan, Olmesartan medoxomil, Tasosartan, Telmisartan, Valsartan |
| 22 | AGTR2 | P50052 | Tasosartan |
| 23 | AHR | P35869 | Atorvastatin, Nimodipine |
| 24 | ANPEP | P15144 | Ezetimibe |
| 25 | ANXA2 | P07355 | Tenecteplase |
| 26 | AOC1 | P19801 | Amiloride |
| 27 | APOB | P04114 | Mipomersen sodium |
| 28 | AR | P10275 | Spironolactone |
| 29 | ASIC2 | Q16515 | Amiloride |
| 30 | ATP1A1 | P05023 | Aluminium, Bepridil, Ethacrynic acid, Hydroflumethiazide, Trichlormethiazide |
| 31 | AVPR1A | P37288 | Conivaptan, Tolvaptan |
| 32 | AVPR2 | P30518 | Conivaptan, Tolvaptan |
| 33 | C1QA | P02745 | Abciximab |
| 34 | C1QB | P02746 | Abciximab |
| 35 | C1QC | P02747 | Abciximab |
| 36 | C1R | P00736 | Abciximab |
| 37 | C1S | P09871 | Abciximab |
| 38 | CA1 | P00915 | Amlodipine, Bendroflumethiazide, Chlorothiazide, Clofenamide, Cyclothiazide, Hydrochlorothiazide, Hydroflumethiazide, Methyclothiazide, Quinethazone, Trichlormethiazide |
| 39 | CA12 | O43570 | Clofenamide, Hydrochlorothiazide, Hydroflumethiazide |
| 40 | CA13 | Q8N1Q1 | Clofenamide |
| 41 | CA14 | Q9ULX7 | Clofenamide |
| 42 | CA2 | P00918 | Bendroflumethiazide, Chlorothiazide, Clofenamide, Cyclothiazide, Furosemide, Hydrochlorothiazide, Hydroflumethiazide, Methyclothiazide, Quinethazone, Trichlormethiazide |
| 43 | CA3 | P07451 | Clofenamide |
| 44 | CA4 | P22748 | Bendroflumethiazide, Chlorothiazide, Clofenamide, Cyclothiazide, Hydrochlorothiazide, Hydroflumethiazide, Methyclothiazide, Trichlormethiazide |
| 45 | CA5A | P35218 | Clofenamide |
| 46 | CA5B | Q9Y2D0 | Clofenamide |
| 47 | CA6 | P23280 | Clofenamide |
| 48 | CA7 | P43166 | Clofenamide |
| 49 | CA8 | P35219 | Clofenamide |
| 50 | CA9 | Q16790 | Clofenamide, Hydrochlorothiazide, Hydroflumethiazide |
| 51 | CACNA1A | O00555 | Bepridil, Spironolactone, Verapamil |
| 52 | CACNA1B | Q00975 | Amlodipine, Spironolactone, Verapamil |
| 53 | CACNA1C | Q13936 | Amlodipine, Barnidipine, Benidipine, Cilnidipine, Clevidipine, Diltiazem, Felodipine, Fendiline, Gallopamil, Isradipine, Lacidipine, Lercanidipine, Lidoflazine, Manidipine, Nicardipine, Nifedipine, Nilvadipine, Nimodipine, Nisoldipine, Nitrendipine, Perhexiline, Spironolactone, Verapamil |
| 54 | CACNA1D | Q01668 | Amlodipine, Barnidipine, Benidipine, Cilnidipine, Clevidipine, Diltiazem, Felodipine, Fendiline, Gallopamil, Isradipine, Lacidipine, Lercanidipine, Lidoflazine, Manidipine, Nicardipine, Nifedipine, Nilvadipine, Nimodipine, Nisoldipine, Nitrendipine, Perhexiline, Spironolactone, Verapamil |
| 55 | CACNA1F | O60840 | Barnidipine, Benidipine, Cilnidipine, Clevidipine, Diltiazem, Fendiline, Gallopamil, Isradipine, Lacidipine, Lercanidipine, Lidoflazine, Manidipine, Nicardipine, Nifedipine, Nilvadipine, Nimodipine, Nisoldipine, Nitrendipine, Perhexiline, Spironolactone, Verapamil |
| 56 | CACNA1G | O43497 | Mibefradil, Spironolactone, Verapamil |
| 57 | CACNA1H | O95180 | Bepridil, Felodipine, Isradipine, Mibefradil, Nifedipine, Nitrendipine, Spironolactone |
| 58 | CACNA1I | Q9P0X4 | Mibefradil, Spironolactone, Verapamil |
| 59 | CACNA1S | Q13698 | Amlodipine, Barnidipine, Benidipine, Cilnidipine, Clevidipine, Diltiazem, Felodipine, Fendiline, Gallopamil, Isradipine, Lacidipine, Lercanidipine, Lidoflazine, Manidipine, Nicardipine, Nifedipine, Nilvadipine, Nimodipine, Nisoldipine, Nitrendipine, Perhexiline, Spironolactone, Verapamil |
| 60 | CACNA2D1 | P54289 | Amlodipine, Felodipine, Isradipine, Nicardipine, Nifedipine, Nilvadipine, Nisoldipine, Nitrendipine, Spironolactone |
| 61 | CACNA2D2 | Q9NY47 | Bepridil, Felodipine, Isradipine, Nitrendipine, Spironolactone |
| 62 | CACNA2D3 | Q8IZS8 | Amlodipine, Nilvadipine, Spironolactone |
| 63 | CACNB1 | Q02641 | Amlodipine, Nimodipine, Spironolactone, Verapamil |
| 64 | CACNB2 | Q08289 | Amlodipine, Felodipine, Isradipine, Nicardipine, Nifedipine, Nilvadipine, Nimodipine, Nisoldipine, Nitrendipine, Spironolactone, Verapamil |
| 65 | CACNB3 | P54284 | Nimodipine, Spironolactone, Verapamil |
| 66 | CACNB4 | O00305 | Nimodipine, Spironolactone, Verapamil |
| 67 | CACNG1 | Q06432 | Diltiazem, Lercanidipine, Nitrendipine, Spironolactone |
| 68 | CALM1 | P62158 | Bepridil, Felodipine, Nicardipine, Nifedipine |
| 69 | CALR | P27797 | Tenecteplase |
| 70 | CANX | P27824 | Tenecteplase |
| 71 | CES1 | P23141 | Probucol |
| 72 | CFTR | P13569 | Bumetanide |
| 73 | CHAT | P28329 | Nicotine |
| 74 | CHRM1 | P11229 | Nicardipine |
| 75 | CHRM2 | P08172 | Nicardipine |
| 76 | CHRM3 | P20309 | Nicardipine |
| 77 | CHRM4 | P08173 | Nicardipine |
| 78 | CHRM5 | P08912 | Nicardipine |
| 79 | CHRNA10 | Q9GZZ6 | Nicotine |
| 80 | CHRNA2 | Q15822 | Nicotine |
| 81 | CHRNA3 | P32297 | Nicotine |
| 82 | CHRNA4 | P43681 | Nicotine |
| 83 | CHRNA5 | P30532 | Nicotine |
| 84 | CHRNA6 | Q15825 | Nicotine |
| 85 | CHRNA7 | P36544 | Nicotine |
| 86 | CHRNA9 | Q9UGM1 | Nicotine |
| 87 | CHRNB2 | P17787 | Nicotine |
| 88 | CHRNB3 | Q05901 | Nicotine |
| 89 | CHRNB4 | P30926 | Nicotine |
| 90 | CLEC3B | P05452 | Tenecteplase |
| 91 | CPT1A | P50416 | Perhexiline |
| 92 | CPT2 | P23786 | Perhexiline |
| 93 | CRYZ | Q08257 | Dicoumarol |
| 94 | CTRB1 | P17538 | Aprotinin |
| 95 | CXCL12 | P48061 | Tinzaparin |
| 96 | CYP11B2 | P19099 | Spironolactone |
| 97 | CYP19A1 | P11511 | Nicotine |
| 98 | DPP4 | P27487 | Atorvastatin |
| 99 | E | P00720 | Timolol |
| 100 | ELANE | P08246 | Alpha-1-proteinase inhibitor |
| 101 | F10 | P00742 | Apixaban, Coagulation factor VIIa, Enoxaparin, Fondaparinux, Fondaparinux sodium, Heparin, Rivaroxaban |
| 102 | F2 | P00734 | Argatroban, ART-123, Bivalirudin, Dabigatran etexilate, Desirudin, Lepirudin, Melagatran, Ximelagatran |
| 103 | F2R | P25116 | Streptokinase |
| 104 | F3 | P13726 | Coagulation factor VIIa |
| 105 | F5 | P12259 | ART-123 |
| 106 | F7 | P08709 | Coagulation factor VIIa |
| 107 | FADS1 | O60427 | Icosapent |
| 108 | FCGR1A | P12314 | Abciximab |
| 109 | FCGR2A | P12318 | Abciximab |
| 110 | FCGR2B | P31994 | Abciximab |
| 111 | FCGR2C | P31995 | Abciximab |
| 112 | FCGR3A | P08637 | Abciximab |
| 113 | FCGR3B | O75015 | Abciximab |
| 114 | FFAR1 | O14842 | Icosapent |
| 115 | FGA | P02671 | Alteplase, Ancrod, Anistreplase, Reteplase, Tenecteplase |
| 116 | FOS | P01100 | Nadroparin |
| 117 | FXYD2 | P54710 | Cyclothiazide |
| 118 | GGCX | P38435 | Coagulation factor VIIa |
| 119 | GJA1 | P17302 | Carvedilol |
| 120 | GUCY1A2 | P33402 | Isosorbide mononitrate |
| 121 | HCAR2 | Q8TDS4 | Nicotinic acid |
| 122 | HCAR3 | P49019 | Nicotinic acid |
| 123 | HDAC2 | Q92769 | Lovastatin |
| 124 | HIF1A | Q16665 | Carvedilol |
| 125 | HMGCR | P04035 | Atorvastatin, Fluvastatin, Lovastatin, Pitavastatin, Pravastatin, Rosuvastatin, Simvastatin |
| 126 | HPN | P05981 | Coagulation factor VIIa |
| 127 | HRH1 | P35367 | Loratadine |
| 128 | HTR1A | P08908 | Alprenolol, Penbutolol, Pindolol, Propranolol |
| 129 | HTR1B | P28222 | Penbutolol, Pindolol, Propranolol |
| 130 | ITGA2B | P08514 | Abciximab, Eptifibatide, Tirofiban |
| 131 | ITGA4 | P13612 | Tinzaparin |
| 132 | ITGAL | P20701 | Lovastatin |
| 133 | ITGB2 | P05107 | Simvastatin |
| 134 | ITGB3 | P05106 | Abciximab, Eptifibatide, Tirofiban |
| 135 | JUN | P05412 | Irbesartan |
| 136 | KCNA1 | Q09470 | Nifedipine |
| 137 | KCNA10 | Q16322 | Verapamil |
| 138 | KCNA3 | P22001 | Verapamil |
| 139 | KCNA7 | Q96RP8 | Verapamil |
| 140 | KCNC2 | Q96PR1 | Verapamil |
| 141 | KCNE1 | P15382 | Indapamide |
| 142 | KCNH2 | Q12809 | Carvedilol, Sotalol, Verapamil |
| 143 | KCNH4 | Q12809 | Sotalol |
| 144 | KCNJ11 | Q14654 | Verapamil |
| 145 | KCNJ6 | P48051 | Verapamil |
| 146 | KCNMA1 | Q12791 | Bendroflumethiazide, Hydrochlorothiazide, Hydroflumethiazide |
| 147 | KCNQ1 | P51787 | Bepridil, Indapamide |
| 148 | KLK1 | P06870 | Aprotinin |
| 149 | KRT8 | P05787 | Tenecteplase |
| 150 | LRP1 | Q07954 | Tenecteplase |
| 151 | LRP2 | P98164 | Bepridil, Felodipine, Nicardipine, Urokinase |
| 152 | LTA4H | P09960 | Captopril |
| 153 | MMP2 | P08253 | Captopril |
| 154 | mmp20 | O43923 | Fenofibrate |
| 155 | MMP9 | P14780 | Captopril |
| 156 | MTTP | P55157 | Lomitapide |
| 157 | MYC | P01106 | Nadroparin |
| 158 | NDUFC2 | O95298 | Carvedilol |
| 159 | NFKB1 | P19838 | Triflusal |
| 160 | NID1 | P14543 | Urokinase |
| 161 | NNMT | P40261 | Nicotinic acid |
| 162 | NOS2 | P35228 | Triflusal |
| 163 | NPC1L1 | Q9UHC9 | Ezetimibe |
| 164 | NPPB | P16860 | Carvedilol |
| 165 | NPR1 | P16066 | Amyl Nitrite, Eritrityl tetranitrate, Erythrityl Tetranitrate, Isosorbide dinitrate, Nesiritide, Nitroglycerin |
| 166 | NPR2 | P20594 | Eritrityl tetranitrate, Erythrityl Tetranitrate, Nesiritide |
| 167 | NPR3 | P17342 | Nesiritide |
| 168 | NQO1 | P15559 | Dicoumarol, Ethyl biscoumacetate, Warfarin |
| 169 | NR3C1 | P04150 | Spironolactone |
| 170 | NR3C2 | P08235 | Canrenone, Eplerenone, Felodipine, Nimodipine, Potassium canrenoate, Spironolactone |
| 171 | P2RY12 | Q9H244 | Clopidogrel, Epoprostenol, Prasugrel, Ticagrelor, Ticlopidine, Treprostinil |
| 172 | PDE10A | Q9Y233 | Dipyridamole, Triflusal |
| 173 | PDE1A | P54750 | Bepridil, Felodipine, Nicardipine |
| 174 | PDE3A | Q14432 | Cilostazol |
| 175 | PDE4A | P27815 | Dipyridamole |
| 176 | PDE4B | Q07343 | Theobromine |
| 177 | PDE5A | O76074 | Dipyridamole |
| 178 | PDXK | O00764 | Pyridoxal |
| 179 | PGR | P06401 | Spironolactone |
| 180 | PLAT | P00750 | Urokinase |
| 181 | PLAU | P00749 | Amiloride, Saruplase, Urokinase |
| 182 | PLAUR | Q03405 | Alteplase, Anistreplase, Reteplase, Tenecteplase, Urokinase |
| 183 | PLG | P00747 | Alteplase, Anistreplase, Aprotinin, Reteplase, Streptokinase, Tenecteplase, Urokinase |
| 184 | PPARA | Q07869 | Aluminium clofibrate, Bezafibrate, Choline fenofibrate, Ciprofibrate, Clofibrate, Etofibrate, Fenofibrate, Gemfibrozil, Simfibrate |
| 185 | PPARD | Q03181 | Bezafibrate, Icosapent, Treprostinil |
| 186 | PPARG | P37231 | Bezafibrate, Icosapent, Telmisartan |
| 187 | PROC | P04070 | Drotrecogin alfa(activated) |
| 188 | PRSS1 | P07477 | Aprotinin |
| 189 | PTGIR | P43119 | Beraprost, Epoprostenol, Iloprost, Treprostinil |
| 190 | PTGIS | Q16647 | Epoprostenol |
| 191 | PTGS1 | P23219 | Acetaminophen, Acetylsalicylic acid, Aloxiprin, Icosapent, Indobufen, Triflusal |
| 192 | PTGS2 | P35354 | Acetaminophen, Acetylsalicylic acid, Aloxiprin, Icosapent, Indobufen |
| 193 | QPRT | Q15274 | Nicotinic acid |
| 194 | REN | P00797 | Aliskiren, Remikiren |
| 195 | SCN1A | P35498 | Amiloride |
| 196 | SCN5A | Q14524 | Ranolazine, Verapamil |
| 197 | SCN9A | Q15858 | Ranolazine |
| 198 | SCNN1A | P37088 | Amiloride, Triamterene |
| 199 | SCNN1B | P51168 | Amiloride, Triamterene |
| 200 | SCNN1D | P51172 | Amiloride, Triamterene |
| 201 | SCNN1G | P51170 | Amiloride, Triamterene |
| 202 | SELE | P16581 | Carvedilol |
| 203 | SELP | P16109 | Dalteparin, Heparin, Nadroparin |
| 204 | SERPINA5 | P05154 | Urokinase |
| 205 | SERPINB2 | P05120 | Tenecteplase, Urokinase |
| 206 | SERPINC1 | P01008 | Ardeparin, Bemiparin, Dalteparin, Danaparoid sodium, Enoxaparin, Fondaparinux sodium, Heparin, Nadroparin, Parnaparin, Reviparin, Sulodexide, Tinzaparin |
| 207 | SERPIND1 | P05546 | Ardeparin, Sulodexide |
| 208 | SERPINE1 | P05121 | Alteplase, Anistreplase, Reteplase, Tenecteplase, Urokinase |
| 209 | SHBG | P04278 | Spironolactone |
| 210 | SLC12A1 | Q13621 | Bumetanide, Chlorthalidone, Etacrynic acid, Ethacrynic acid, Furosemide, Hydroflumethiazide, Methyclothiazide, Muzolimine, Piretanide, Quinethazone, Torasemide, Trichlormethiazide |
| 211 | SLC12A2 | P55011 | Bumetanide, Etacrynic acid, Furosemide, Muzolimine, Piretanide, Quinethazone, Torasemide |
| 212 | SLC12A3 | P55017 | Bendroflumethiazide, Chlorothiazide, Chlortalidone, Hydrochlorothiazide, Hydroflumethiazide, Indapamide, Mefruside, Methyclothiazide, Meticrane, Metolazone, Polythiazide, Quinethazone, Trichlormethiazide |
| 213 | SLC12A4 | Q9UP95 | Bumetanide |
| 214 | SLC12A5 | Q9H2X9 | Bumetanide |
| 215 | SLC18A2 | Q05940 | Ephedrine |
| 216 | SLC29A1 | Q99808 | Dipyridamole |
| 217 | SLC6A2 | P23975 | Ephedrine |
| 218 | SLC6A4 | P31645 | Verapamil |
| 219 | SLC8A1 | P32418 | Icosapent |
| 220 | SLC9A1 | P19634 | Amiloride |
| 221 | SMPD1 | P17405 | Amlodipine |
| 222 | SOAT1 | P35610 | Ezetimibe |
| 223 | SRD5A1 | P18405 | Spironolactone |
| 224 | SRD5A2 | P31213 | Spironolactone |
| 225 | SRD5A3 | Q9H8P0 | Spironolactone |
| 226 | ST14 | Q9Y5Y6 | Urokinase |
| 227 | TBXA2R | P21731 | Picotamide, Ridogrel |
| 228 | TBXAS1 | P24557 | Ridogrel |
| 229 | TF | P02787 | Aluminium, Coagulation factor VIIa, Dalteparin |
| 230 | TFPI | P10646 | Coagulation factor VIIa, Dalteparin |
| 231 | THRA | P10827 | Dextrothyroxine |
| 232 | THRB | P10828 | Dextrothyroxine |
| 233 | TNNC1 | P63316 | Bepridil, Felodipine |
| 234 | TNNC2 | P02585 | Felodipine |
| 235 | TPO | P07202 | Dextrothyroxine |
| 236 | TRPV1 | Q8NER1 | Icosapent |
| 237 | VCAM1 | P19320 | Carvedilol |
| 238 | VEGFA | P15692 | Carvedilol, Dalteparin |
| 239 | VKORC1 | Q9BQB6 | Acenocoumarol, Clorindione, Dicoumarol, Diphenadione, Ethyl biscoumacetate, Fluindione, Phenindione, Phenprocoumon, Tioclomarol, Warfarin |
| 240 | VTN | P04004 | Abciximab |
