# Supplementary material for: Natural formulas and the nature of formulas: Exploring potential therapeutic targets based on traditional Chinese herbal formulas
Source: PLoS One. 2017 Feb 9;12(2):e0171628. doi: 10.1371/journal.pone.0171628 (PMC5300118; doi:10.1371/journal.pone.0171628)
Supplement: S7 Table — (DOCX) [file pone.0171628.s007.docx]

S7 Table. Common proteins of XZD and GXBD

| ID | Proteins | Anti-CAD drug targets | ID | Proteins | Anti-CAD drug targets |
| --- | --- | --- | --- | --- | --- |
| 1 | NOS2 | Yes | 106 | SRC | No |
| 2 | PTGS1 | Yes | 107 | CD86 | No |
| 3 | DRD1 | No | 108 | CD80 | No |
| 4 | INSR | No | 109 | DPP4 | Yes |
| 5 | CHRM3 | Yes | 111 | PLAU | Yes |
| 6 | F2 | Yes | 113 | PYGM | No |
| 8 | NOS1 | No | 114 | IL6 | No |
| 9 | KCNA4 | No | 115 | MMP1 | No |
| 10 | CHRM1 | Yes | 116 | MAPK1 | No |
| 12 | ESR1 | No | 117 | ADORA2A | Yes |
| 13 | AR | Yes | 118 | PON1 | No |
| 14 | ALDH2 | No | 121 | CTSD | No |
| 15 | ALDH5A1 | No | 122 | IFNG | No |
| 16 | ADRB1 | Yes | 123 | FASN | No |
| 19 | SCN5A | Yes | 124 | CTNNB1 | No |
| 21 | PLG | Yes | 126 | LTF | No |
| 22 | SLC7A1 | No | 131 | MAPK14 | No |
| 23 | PPARG | Yes | 132 | TRPV1 | Yes |
| 24 | F10 | Yes | 135 | JUN | Yes |
| 26 | LPL | No | 137 | CCL2 | No |
| 27 | BCL2 | No | 138 | IL1B | No |
| 28 | CHRM5 | Yes | 139 | MAPK3 | No |
| 29 | ALOX5 | No | 140 | GSK3B | No |
| 30 | SLC6A1 | No | 141 | SELE | Yes |
| 31 | PNP | No | 142 | MPO | No |
| 32 | ABAT | No | 143 | CDK1 | No |
| 33 | PTGS2 | Yes | 144 | PLAT | Yes |
| 34 | NOS3 | No | 145 | GJA1 | Yes |
| 36 | REN | Yes | 147 | VCAM1 | Yes |
| 37 | ADRA2A | Yes | 148 | MMP3 | No |
| 38 | SHMT2 | No | 149 | HSP90AA1 | No |
| 39 | PRKDC | No | 150 | THBD | No |
| 40 | HTR3A | No | 152 | SELP | Yes |
| 41 | CA2 | Yes | 153 | F3 | Yes |
| 42 | F7 | Yes | 154 | MMP8 | No |
| 44 | ADRA2C | Yes | 155 | NQO1 | Yes |
| 49 | GABRA2 | No | 156 | MMP12 | No |
| 51 | KYNU | No | 157 | RHO | No |
| 52 | ODC1 | No | 158 | CDK2 | No |
| 53 | CHRM4 | Yes | 159 | BTK | No |
| 54 | RXRA | No | 160 | PIK3CG | No |
| 56 | OPRD1 | No | 161 | ampC | No |
| 57 | OAT | No | 164 | LTA4H | Yes |
| 58 | ACHE | Yes | 165 | CDC25B | No |
| 59 | GLRA1 | No | 167 | TXNRD1 | No |
| 60 | PDE3A | Yes | 168 | BCHE | No |
| 61 | HTR2A | No | 169 | MAOB | No |
| 62 | GABRA5 | No | 170 | MAOA | No |
| 63 | SLC6A2 | Yes | 171 | XDH | No |
| 64 | LDLR | No | 173 | ADA | Yes |
| 65 | ADRA1A | Yes | 175 | ENPEP | No |
| 67 | GABRA3 | No | 176 | CHRNA7 | Yes |
| 68 | GOT1 | No | 177 | PTGER3 | No |
| 69 | HTR2C | No | 178 | SOD1 | No |
| 71 | PGR | Yes | 179 | JAK2 | No |
| 72 | CHRM2 | Yes | 181 | CYP3A4 | No |
| 73 | ADRA2B | Yes | 182 | AMY2A | No |
| 74 | ADRA1B | Yes | 184 | TP53 | No |
| 77 | PTPN1 | No | 185 | CHEK1 | No |
| 78 | ACACA | No | 192 | CELA1 | No |
| 80 | GRIA1 | No | 193 | PRKACA | No |
| 81 | MMP2 | Yes | 196 | PLA2G1B | No |
| 82 | SLC6A3 | No | 197 | HRV-1A | No |
| 83 | GSR | No | 198 | HNF4A | No |
| 85 | NR3C2 | Yes | 200 | CYPIA2 | No |
| 86 | ADRB2 | Yes | 202 | ADH1B | No |
| 87 | TNF | No | 203 | ADH1C | No |
| 88 | RXRG | No | 205 | ADH1A | No |
| 89 | ADRA1D | Yes | 206 | ARG1 | No |
| 90 | BCAT2 | No | 207 | COL1A1 | No |
| 91 | CHRNA2 | Yes | 208 | GSTP1 | No |
| 92 | TOP2A | No | 209 | EGF | No |
| 93 | AKR1B1 | No | 210 | CAT | No |
| 94 | SLC6A4 | Yes | 211 | VEGFA | Yes |
| 98 | GRIN2A | No | 213 | POR | No |
| 99 | EGFR | No | 214 | RRM1 | No |
| 100 | OPRM1 | No | 215 | PKLR | No |
| 101 | ABCC1 | No | 216 | DHODH | No |
| 102 | ESR2 | No | 219 | NTRK2 | No |
| 103 | NR3C1 | Yes | 222 | SULT1E1 | No |
| 104 | GABRA1 | No | 226 | RXRB | No |
| 105 | MGAM | No | 228 | pobA | No |

Notes: Totally 164 proteins are listed in S7, while the ID of protein is corresponded to the label of vertex in Fig 1.
